# Supplementary material for: Status of human onchocerciasis transmission in the Adamaoua region of Cameroon after 20 years of ivermectin mass distribution
Source: PLoS Negl Trop Dis. 2025 Mar 4;19(3):e0011511. doi: 10.1371/journal.pntd.0011511 (PMC11925462; doi:10.1371/journal.pntd.0011511)
Supplement: S1 File — (DOCX) [file pntd.0011511.s001.docx]

**S1 File. Data on nodule and mf prevalences collected in 1998-2001, 2010-2013 and 2019-2020**
