# Supplementary material for: Status of human onchocerciasis transmission in the Adamaoua region of Cameroon after 20 years of ivermectin mass distribution
Source: PLoS Negl Trop Dis. 2025 Mar 4;19(3):e0011511. doi: 10.1371/journal.pntd.0011511 (PMC11925462; doi:10.1371/journal.pntd.0011511)
Supplement: S2 File — (DOCX) [file pntd.0011511.s002.docx]

**S2 File. Geographic cordinates and mf prevalences recorded 1998-2001 to 2020 in 24 communities**
