# Supplementary material for: Status of human onchocerciasis transmission in the Adamaoua region of Cameroon after 20 years of ivermectin mass distribution
Source: PLoS Negl Trop Dis. 2025 Mar 4;19(3):e0011511. doi: 10.1371/journal.pntd.0011511 (PMC11925462; doi:10.1371/journal.pntd.0011511)
Supplement: S3 File — (DOCX) [file pntd.0011511.s003.docx]

**S3 File. Therapeutic coverages reported by the NOCP from 2014 to 2019 and surveyed coverages (2020)**
