# Supplementary material for: Status of human onchocerciasis transmission in the Adamaoua region of Cameroon after 20 years of ivermectin mass distribution
Source: PLoS Negl Trop Dis. 2025 Mar 4;19(3):e0011511. doi: 10.1371/journal.pntd.0011511 (PMC11925462; doi:10.1371/journal.pntd.0011511)
Supplement: S5 File — (DOCX) [file pntd.0011511.s005.docx]

**S5 File. Percentage of people treated in the age groups of the study population**
