# Supplementary material for: Status of human onchocerciasis transmission in the Adamaoua region of Cameroon after 20 years of ivermectin mass distribution
Source: PLoS Negl Trop Dis. 2025 Mar 4;19(3):e0011511. doi: 10.1371/journal.pntd.0011511 (PMC11925462; doi:10.1371/journal.pntd.0011511)
Supplement: Attachment — Submitted filename: PNTD-D-23-00854_RN.pdf [file pntd.0011511.s007.pdf]

# PLOS Neglected Tropical Diseases

## Elimination of Onchocerciasis in the Adamaoua region of Cameroon: A big step towards the end.

--Manuscript Draft--

| | |
|---|---|
| **Manuscript Number:** | PNTD-D-23-00854 |
| **Full Title:** | Elimination of Onchocerciasis in the Adamaoua region of Cameroon: A big step towards the end. |
| **Short Title:** | End of onchocerciasis in Adamaoua region of Cameroon |
| **Article Type:** | Research Article |
| **Keywords:** | onchocerciasis; elimination; ivermectin; CDTI; Adamaoua Region; Cameroon |
| **Abstract:** | Introduction<br>~~Although evidences of onchocerciasis elimination are reported in some foci in West and East Africa, in Central Africa the situation does not sound promising, despite two decades of ivermectin treatment in some foci of the sub-region.~~ This paper describes the current situation of onchocerciasis after 20 consecutive years of mass treatment in the Adamaoua Region in Cameroon.<br>Material and methods<br>The study was conducted between August and September 2020 in Adamaoua Region in Cameroon. The onchocerciasis endemicity was assessed through parasitological clinical diagnosis. Microfilarodermia and nodule prevalences assessed in 2020 were compared to the records of1998-2002 and 2010-2013 surveys using ANOVA and Welch's unequal variances t-test.<br>Results<br>A total of 4,814 participants aged between 5 and 108 years, including 50.4% men and 49.6% women were enrolled in the study, with a sex ratio of 1: 1. For the whole region the nodule and microfilaria prevalence were 0.87 (0.64-1.19) % and 0.77 (0.54-1.07) % respectively. At the community level, the mf prevalences ranged from 0.5% to 4.5%. Globally, the community microfilarial loads (CMFL) were < 0.5 mf/ss. The surveyed [illegible]apeutic coverage rates were between 40% and 78%. The adherence rates in [illegible]mectin treatment of all age groups of the population were less than 65%, except for the 40-50 age group where it was ≈70%.<br>Conclusion<br>The results of this study show a drastic decline in onchocerciasis prevalences after 20 consecutive years of CDTI, indicating a significant progress towards the elimination of the disease in the Adamaoua region. However, additional efforts are needed to increase the adherence of all age groups in Ivermectin treatment to achieve the goal of elimination in this Region. These data are useful for the ministry of health in guiding the elimination procedure of onchocerciasis in Cameroon |
| **Additional Information:** | |
| **Question** | **Response** |
| **Government Employee**<br><br>Are you or any of the contributing authors an employee of the United States government?<br><br>Manuscripts authored by one or more US Government employees are not copyrighted, but are licensed under a CC0 Public Domain Dedication, which allows | No - No authors are employees of the U.S. government. |

| | |
|---|---|
| unlimited distribution and reuse of the article for any lawful purpose. This is a legal requirement for US Government employees.<br><br>This will be typeset if the manuscript is accepted for publication. | |
| **Financial Disclosure**<br><br>Enter a financial disclosure statement that describes the sources of funding for the work included in this submission. Review the submission guidelines for detailed requirements. View published research articles from *PLOS Neglected Tropical Diseases* for specific examples.<br><br>This statement is required for submission and **will appear in the published article** if the submission is accepted. Please make sure it is accurate.<br><br>**Funded studies**<br>Enter a statement with the following details:<br>• Initials of the authors who received each award<br>• Grant numbers awarded to each author<br>• The full name of each funder<br>• URL of each funder website<br>• Did the sponsors or funders play any role in the study design, data collection and analysis, decision to publish, or preparation of the manuscript?<br><br>Did you receive funding for this work? | The author(s) received no specific funding for this work. |
| **Competing Interests**<br><br>On behalf of all authors, disclose any competing interests that could be perceived to bias this work.<br><br>This statement will be typeset if the manuscript is accepted for publication. | No competing interests |

| | |
|---|---|
| Review the instructions link below and PLOS NTDs' competing interests policy to determine what information must be disclosed at submission. | |
| **Data Availability**<br><br>Provide a **Data Availability Statement** in the box below. This statement should detail where the data used in this submission can be accessed. This statement will be typeset if the manuscript is accepted for publication.<br><br>Before publication, authors are required to make all data underlying their findings fully available, without restriction. Review our PLOS Data Policy page for detailed information on this policy. Instructions for writing your Data Availability statement can be accessed via the Instructions link below. | Th[illegible]ata we have presented in this paper may be free of access to the general public a[illegible]hould not be restricted. They should be accessible on the Internet without any particular requirements, as they will help to inform the scientific community about the situation of onchocerciasis in the Adamaoua region. |

Manuscript Click here to access/download;Manuscript;Manuscript_Adamaoua_Submitte

# Elimination of Onchocerciasis in the Adamaoua region of Cameroon: A big step towards the end.

Philippe Nwane[*1,2], Hugues Clotaire Nana-Djeunga[1], Narcisse Nzune Toche[1], André Domché[1,2], Fesuh Nono Bertrand[3], Yannick Emalio Niamsi[1], Guy Roger Njitchuang[1], Martine Augusta Flore Tsasse[1], Jean Bopda[1], Steve Bickmen[1], Aubin Balog[1], Alexis Nkwelle[4], Patrice Nkwelle[4†], Clarisse Ebene[5], Honoré Obama[5], Paul Messi[5], Benjamin Biholong[5], Serge Billong[5], Georges Nko'o Ayissi[6] and Joseph Kamgno[1,7]

**Author details**

[1]Higher institute of scientific and medical research (ISM), P.O. Box 5797 Yaoundé, Cameroon ;
[2]Laboratory of General Biology, University of Yaounde I, P.O. Box 337, Yaoundé, Cameroon
[3]National Advanced School of Engineering, University of Yaoundé I, Yaoundé, Cameroon
[4]International eye Foundation, Cameroon ;
[5]National Onchocerciasis Control Program/Ministry of Public Health, Yaounde, Cameroon ;
[6]Departement of Neglected Tropical Diseases Department/Ministry of Public Health, Yaounde, Cameroon ;
[7]Faculty of Medicine and Biomedical Sciences, University of Yaounde I, Yaounde, Cameroon.

† deceased some months after the data analysis.

## Abstract

### Introduction

Although evidences of onchocerciasis elimination are reported in some foci in West and East Africa, in Central Africa the situation does not sound promising, despite two decades of ivermectin treatment in some foci of the sub-region. This paper describes the current situation of onchocerciasis after 20 consecutive years of mass treatment in the Adamaoua Region in Cameroon.

### Material and methods

The study was conducted between August and September 2020 in Adamaoua Region in Cameroon. The onchocerciasis endemicity was assessed through parasitological clinical diagnosis. Microfilarodermia and nodule prevalences assessed in 2020 were compared to the records of1998-2002 and 2010-2013 surveys using ANOVA and Welch's unequal variances t-test.

### Results

A total of 4,814 participants aged between 5 and 108 years, including 50.4% men and 49.6% women were enrolled in the study, with a sex ratio of 1: 1. For the whole region the nodule and microfilaria prevalence were 0.87 (0.64-1.19) % and 0.77 (0.54-1.07) % respectively. At the community level, the mf prevalences ranged from 0.5% to 4.5%. Globally, the community microfilarial loads (CMFL) were < 0.5 mf/ss. The surveyed therapeutic coverage rates were between 40% and 78%. The adherence rates in ivermectin treatment of all age groups of the population were less than 65%, except for the 40-50 age group where it was ≈70%.

**Conclusion**

The results of this study show a drastic decline in onchocerciasis prevalences after 20 consecutive years of CDTI, indicating a significant progress towards the elimination of the disease in the Adamaoua region. However, additional efforts are needed to increase the adherence of all age groups in Ivermectin treatment to achieve the goal of elimination in this Region. These data are useful for the ministry of health in guiding the elimination procedure of onchocerciasis in Cameroon.

**Keywords**: Onchocerciasis - Elimination - Ivermectin - CDTI - Adamaoua Region - Cameroon.

**Author summary**

Onchocerciasis control relies on mass treatment of populations with an annual dose of ivermectin. It was originally assumed that an onchocerciasis-endemic community treated using the community-directed ivermectin (CDTI) approach should become free of the disease after 15 years, a period corresponding to the maximum lifespan of the adult worm in humans. Using this approach, the disease has been eliminated in a few foci in Mali, Niger, Senegal and Nigeria in West Africa, and in Uganda in East Africa. In Central Africa, elimination of onchocerciasis remains uncertain after 20 years of mass treatment conducted in certain foci. The data presented in this study are very promising, showing that elimination of the disease using the CDTI approach is possible in foci of Adamaoua region in Cameroon. Achieving the goal elimination in this part of the country in the near future requires little efforts of the population in adhering to ivermectin treatment. This will involve raising awareness among men and women of all age groups eligible for ivermectin treatment.

## 1. Rationale and background

Also known as “River Blindness”; onchocerciasis is caused by a parasitic worm *Onchocerca volvulus* transmitted from person to person through bites of blackflies belonging to the genus *Simulium.* Over the course of few months to one year, the transmitted $L_3$ infective stage of the parasite molt twice and develop into sexually mature adult. Female worms live in fibrous nodules of the skin where it remains sessile, while male individual migrates between the nodules to mate with settled females. Both female and male adult worms live between 2 and 15 years with an average life expectancy of 10 years [1,2]**.** During this period patent females release between 700 and 1,500 microfilariae (mf) per day [1,3]**.** which predominantly live in the skin for 3 to 5 years causing lesions including intense itching, thickening, depigmentation, and loss of skin elasticity. Other clinical manifestations such as hanging

groin, skin atrophy, papular onchodermatitis and chronic papular onchodermatitis, lichenified onchodermatitis are commonly noted [4]. The microfilaria (mf) can migrate throughout the intercellular fluid and reach the eye causing ocular manifestations which can lead to irreversible blindness [5]. Although the physiopathology is not well known, several studies have shown associations between onchocerciasis, epilepsy and nodding syndrome [3,6,7,8,9,10].

Onchocerciasis is distributed in the world with most of cases recorded in Sub-Saharan Africa (SSA) [11,12,13,14]. Historical data from SSA show a bleak picture of this disease in several endemic villages where prevalences of infected individuals sometimes exceeded 80%, and the rates of onchocercal blindness around 10% [15,16,17]. In regard with the gravity of this severe infectious disease, private, public, national and international health authorities/agencies have set the objective to reduce the prevalence and transmission to a point where it should no longer be of public health or socio-economic concern in affected countries [18,19]. Since then, the control of the disease has been thus fare based on 2 major large-scale strategies including (1) vector control targeting *Simulium* blackflies and (2) parasite control targeting *O. volvulus* in human. These two strategies have been implemented at large scale in SSA to alleviate the burden of the disease in human populations. The first was implemented in West Africa between 1974–2002 as a core activity of the Onchocerciasis Control Programme (OCP) [15]. The second strategy targeting parasite control was proposed with the discovery of a safe and effective microfilaricide, ivermectin known under the brand name Mectizan® [ 20]. The distribution of this drug since 1987 freely donated by the pharmaceutical company Merck & Co. [21], has prolonged the control of onchocerciasis in West Africa with extension in endemic countries of Central and East Africa under the management of the African Program for Onchocerciasis Control (APOC) [22,23]. The APOC adopted Community-Directed Treatment with Ivermectin (CDTI) as a core strategy with the objective to establish a mechanism for sustained delivery of an annual dose of ivermectin to the entire eligible populations in meso- and hyperendemic communities [ 24,25]. Based on the lifespan of adult worms in humans, the expectation of eliminating onchocerciasis after 15 consecutive years was a convincing idea for testing vector control in West Africa and implementing CDTI in endemic countries in SSA [26]. In countries where the impact of Vector control and CDTI has been remarkable, the duration of the implementation was above 15 years, corresponding to the maximum life expectancy of adult worms in humans [ 27].

Evidences of onchocerciasis elimination through CDTI approach in some foci of Latin America and SSA [28,29,30,31,32,33,34,35] have supported the idea of shifting from the control to elimination of the disease [36]. Besides, the evidences, it is increasingly recognized that onchocerciasis is no longer a public health or socio-economic concern after 15 to 20 successive years of CDTI in some foci in West Africa [37] In Central Africa, the such an outcome remains anecdotal after 20 years of CDTI, without any hope or evidence of eliminating the disease.

Epidemiological assessments conducted in Cameroon demonstrated that the transmission of onchocerciasis is still significantly ongoing even in foci where CDTI has been implemented for more than 15 years [35,38,39]. Those conducted by APOC in 2012 in 7 CDTI projects in Cameroon showed that elimination of onchocerciasis was feasible in the short term in the Adamaoua II project, according to the decline in microfilarial prevalences noted in the region [37]. Since then, the Adamaaou region, like all the others in Cameroon, has been receiving mass treatments of ivermectin every year. This paper reports the situation of onchocerciasis prevalence in the Adamaoua Region after 20 consecutive years of mass treatment of populations with ivermectin.

## 2. Materials and methods

### 2.1 Study area

This study was conducted in the Adamaoua Region of Cameroon located between the 6$^{th}$ and 8$^{th}$ degrees of Latitude North and the 10$^{th}$ and 16$^{th}$ degrees of Longitude East. The region covers a surface area of about 62,000 km$^2$, with altitude ranging between 900 and 1500 m. It is situated in the transition zone between the humid equatorial climate of the South and the tropical dry sudano-sahelian climate of the North, influenced by the Adamaoua plateau [40]. The mean annual rainfall varies between 900 mm to 1,500 mm and is distributed in a continuous rainy season from April to October [41]. A dry season occurs from November to March with a mean annual temperature ranging between 23°C and 26°C [42]. Most of the watercourses in Cameroon have their source in the Adamaoua Region, so called the "water tower of Cameroon". The main rivers of the region include, Mayo Deo Mbéré and Vina rivers which are of a great importance to human activities such as agriculture and livestock breeding [43,44]. These rivers exhibit in their watercourse, rapids, cascades and waterfalls favorable to the establishment of *Simulium* blackfly populations.

The health map divides the Region in 9 health districts including Ngaoundéré Rural, Ngaoundéré Urbain, Bankim, Tibati, Banyo, Ngaoundal, Tignère, Djohong and Meiganga. Onchocerciasis is known to be endemic in the Adamaoua Region for around 10 decades [45]. A total of 29 communities spread across the 9HDs were selected, with priority given to those visited during the 1998-2002 Rapid Epidemiological Mapping of Onchocerciasis (REMO) conducted in the region.

### 2.2.2 Study design / Data collection

A cross-sectional survey was conducted between August and September 2020. Data collection was carried out in several screening points or local health facilities of the region. Participation in the study was supported by permanent status as a community member and having at least 5 years. A flow of community members participating in the study was defined in three work stations including (1) a registration station, (2) a skin biopsy collection station, and (3) a medical consultation station (**figure 1**). At the registration station, all adult participants provided written informed consent. For participants aged below 15 years, a parental or legal guardian consent was required for their enrolment. Personal information of each participant (gender, age, function, permanent residence,…) was recorded and a barcode number was assigned to each eligible participant to keep track of the data. At the skin biopsy collection station, 2 superficial skin biopsies were collected from each participant at the left and right posterior iliac crests using a sharp and sterilized 2-mm Holthtype corneoscleral punch. Immediately after collection, each skin biopsy was individually placed into a well of a round-bottom 96-well microtiter plate containing approximately 40μL of physiological saline per well. Plate containing skin biopsies were incubated for 24 hours at room temperature to induce microfilaria emergence [46]. At the end of this period, plates were transferred to the local field laboratory for skin biopsy sample examination.
In the laboratory, the physiological saline was pipetted out and placed on a slide then examined microscopically 40x magnification for the search of microfilaria and counting when they were identified.
At the medical consultation station, the participant was subjected to onchocerciasis nodule palpation examination which consists of searching subcutaneous nodules over the body and the skin manifestations of the disease. The participant was then interviewed about his ivermectin treatment history. The answers provided following the interview were recorded on his registration form, submitted to the laboratory for archiving result of his skin biopsies examination. In the framework of this study, the methods described by Coffeng and

colleagues [47] were used to convert nodule prevalences of the 1999-2001 survey (typically in samples of adult males aged ≥ 20 years) into microfilarial prevalences (in the population aged ≥ 5 years).

### 2.3 Data analysis

The information provided by each participant at the three workstations and the results of microscopic analysis of its two skin biopsies were recorded in a specially prepared Excel file. The main indicators of onchocerciasis including microfilaria (mf) and nodule prevalences were expressed as a percentage i.e number of persons positive divided by the number of persons examined × 100. The arithmetic mean of the mf from the two skin biopsies from each participant was calculated and used as a measure of intensity of infection. The Community microfilarial load (CMFL) was calculated as anti-log $\{(\sum \log(x+1))/n\}$ -1, with **x** being the mean of mf/mg of skin and n the number of individuals examined [48]. The chi-square test was used to compare: (1) the microfilaria and nodule prevalences obtained in this study with those obtained in 1998-2002 (baseline) and 2010-2013 (APOC evaluation), (2) and the adherence rate of men to ivermectin treatment in 2019 in each health district. A two-tailed p-value lower than 0.05 was considered statistically significant.

## 4.Results

### 4.1 Demographic data

A total of 4,814 participants from 29 communities selected in the 9 Health Districts of the Adamaoua region were enrolled in this survey. Men represented 50.4% (N = 2,424) and women 49.6% (N =2,390) with a sex ratio of 1 :1. Participants were aged between 5 and 108 years old (median…). The distribution of participants according to the study population by age group and gender is shown in **figure 2**. Participants aged between 5 and 30 years represent more than half of the study population (58.45%).

### 4.2 Status of *O. volvulus* infection in surveyed communities and health districts

Of the 29 communities surveyed during this study, ~~11 (≈38%)~~ unevenly distributed in 7 HDs were infected by *O. volvulus* microfilariae **(table 1)**. Of these, four were from Meiganga HD, 2 from Ngaoundal HD and 1 from each in the following HDs: Bankim, Djohong, Ngaoundéré Rural, Ngaoundéré Urbain and Tibati. No infected community was identified in the Banyo

and Tignere HDs. The mf prevalence among infected communities ranged from 0.50% [95%CI: 0.02 - 3.23] in Mbakaou of Tibati HD to 4.34% [95%CI: 1.77-9.63] in Nandeke of Meiganga HD. These mf prevalences were significantly lower ($p < 0.05\%$) compared to that recorded in the 1998-2002 baseline survey where prevalences varied between 36.7% (from Alhamissa in Tibati HD) and 87.7% (from Djamtari in Tignere HD). Of the 29 communities surveyed in the 2020 follow-up survey, 22 were previously prospected in the 1998-2002 baseline survey. The variation trend in mf prevalences of these 22 communities between the 1998-2002 baseline survey and the 2020 follow-up is shown in **figure 3**. Of these 22 communities, the 2020 survey revealed that 13 (59%) were found free of *O.volvulus* infection and 9 (41%) showed mf prevalences below 5% (**table 1**). In general, the intensity of *O. volvulus* infection characterized by the community microfilarial load (CMFL) was very low (< 0.5 mf/ss) in each of the surveyed communities. The CMFLs assessed in the 2020 survey range from 0.006 mf/ss in Wakwa (Ngaoundere Urbain HD) to 0.129 mf/ss in Nandeke (Meiganga HD). When assessed for participants aged more than 20 years, the CMFLs varied from 0 mf/ss in Mbakaou (Tibati HD) to 0.0716 mf/ss in Zouzami (Meiganga HD) (**table 1**). The mf and nodule prevalences were estimated at a broader geographical scale level of each health district by grouping together communities belonging to the same HD. Table 3 summarizes the prevalences obtained in the 2020 survey, and Figure 3 shows the trend in their evolution in relation to those recorded in 1998-2002, 2010-2013. At the level of HDs, the prevalences ranged from 0% in Meiganga HD to 2.36% in Tibati HD. Those recorded during the 1998-2002 baseline survey ranged between 33.5% in Ngaoundal HD and 66.6% in Tignère HD. At the regional level, the nodule prevalence recorded was 0.87% (95% CI: 0.64-1.19) % (**table 2**). Overall, the nodule prevalences of this 2020 survey were significantly lower ($p < 0.05$) compared to that recorded in the 1998-2002 baseline survey (**figure 4 A**). Concerning microfilaria, the prevalences recorded during the 2020 survey from 0% in Banyo and Tignère HDs to 3.4% in Meiganga HD. At the regional level the mf prevalence recorded was 0.77% (95% CI: 0.54-1.07). Those recorded during the 2010-2013 survey varied between 0.6% in Banyo HD to 2.6% in Bankim HD, while those of the 1998-2002 baseline survey ranged from 51% in Ngaoundal HD to 72% in Ngaoundére rural HD (**figure 4 B**). Globally, the mf prevalences of the 2020 survey were significantly lower ($p < 0.05$) than those of the 1998-2002 baseline survey, but not significantly different from those of the 2010-2013 survey in Bankim and Meiganga HDs ($p > 0.05$), with a non-significant

increase in mf prevalence in Meiganga HD and a decrease in Bankim HD noted during the 2020 survey (**figure 4 B**)

### 4.3 Trend in ivermectin treatment

Of the 4,814 participants interviewed regarding ivermectin treatment, ≈28% (N=1,351) said they had never been treated, while ≈72% (N=3,463) declared having received the treatment at least once in the past 6 years (2014-2019). Among those who were treated, ≈86% (N=2,977) received the last treatment in 2019, about 9.7% (N=336) in 2018 and 4% (N=141) in 2017, 2016 2015 and 2014. It should be mentioned that the prevalence of microfilaria in those who did not comply with ivermectin treatment (p =0.7%) was almost equivalent to that observed in those who accepted the treatment (p=0.8). The HD coverage rates in ivermectin treatment reported by the PNLO over the past 6 years from 2014 to 2019) and those obtained from participant statements during the 2020 survey are shown in **figure 5**. Overall, all communities in the 9 HDs of Adamaoua Region are under ivermectin mass treatment, resulting in a 100% geographical coverage. The TCRs reported by the NOCP for the 9 HDs over the past six years (2014-2019) preceding the 2020 survey varied between 71.9% in Tibati HD in 2016 and 87.6% in Ngaoundere Rural HD in 2017. The number of HDs achieving at least 80% a therapeutic coverage rate increased significantly from 2014 to 2019. As for the STCRs, they ranged from 40% in Meiganga HD to 77.5% in Tibati HD, with six HDs namely Banyo, Bankim, Djohong, Ngaoundéré Urbain, Ngaoundal and Meiganga showing STCRs below 65%. The remaining 3 HDs including Ngaoundéré rural, Tibati and Tignere showed STCRs above 65% and below 80%.

In this study, adherence rates of population to ivermectin treatment (i.e the percentage of people who took ivermectin treatment among the eligible population in the community or HD) by gender and age group were also investigated. The population adherence to ivermectin treatment by sex in the HDs is shown in **figure 6**. At the regional level, men and women showed comparable adherence rates during the 2019 ivermectin mass distribution campaign i.e 48.1% (N=1,433) and 51.9% (N=1,544) respectively ($p > 0.05\%$). Within the HDs, women's adherence was significantly higher in Bankim and Meiganga HDs, while that of men was higher in Ngaoundéré Rural, Tibati and Tignere HDs. In the other 4 HDs including Banyo, Djohong, Ngaoundal and Ngaoundéré Urbain both men and women showed similar adherence with however a noticeable increase but not significant in women adherence in Banyo HD and men adherence in Djohong, Ngaoundal and Ngaoundéré

Urbain HDs. The population adherence rates in ivermectin treatment by age group are shown in **figure 7**. Overall, all age groups showed adherence rates below 65%, with however an exception in the 40-50 age group where the rate was ≈ 70%. Except for the 70+ age group, the 20-30 age group showed a significantly adherence rate than all other age groups ($p < 0.05$).

## 5. Discussion

The objective of this study was to assess the progress made towards the elimination of onchocerciasis in the Adamaoua Region after two decades of mass treatment with ivermectin. Overall, the nodule and microfilaria prevalences recorded during this 2020 epidemiological study in the surveyed communities and HDs of the region were below 5% with most of them exhibiting 0 mf prevalence. Based on the criteria applied for delineating onchocerciasis foci into endemicity levels, all these communities and HDs may be currently classified as sporadic for onchocerciasis [49] or hypo-endemic [50,51]. All the CMFLs assessed in 11 over 29 communities surveyed for residents aged 20 years and above according to Remme and Colleagues [52] showed less than 1 mf/sb (skin biopsy). Even taking into account all members eligible to ivermectin treatment and belonging to the same community, the CMFLs assessed were found below 1mf/sb. The mf prevalences and the CMFLs recorded in this study are fare below of that reported in the same communities during the 1998-2002 REMO data collection in Cameroon [53,54]. This decline in onchocerciasis intensity has previously been reported in Adamaoua II CDTI zone during the 2013 epidemiological assessments conducted in Cameroon as part of the impact evaluation of the CDTI strategy in countries of the African Programme for Onchocerciasis Control [37]. The low prevalences of the disease recorded in the surveyed communities is attributed to the efforts put in place in controlling the disease through an effective involvement of communities in ivermectin mass distribution during the past 20 consecutive years. In the context of achieving the elimination of onchocerciasis by 2030, data presented in this study are promising compared to that recently reported in other regions of Cameroon [55,56,57].

The slightly increase in mf prevalence noted in Meiganga HD during this 2020 study compared to that of the 2013 may be attributed to the slackening of the populations in ivermectin treatment. This reflects the low ivermectin treatment adherence observed across all age groups of the eligible population in the whole region, and particularly the remarkable low adhesion of men (≈ 43%) noted in this HD. Also, the existence in the region particularly

in Meiganga, Ngaoundal and Ngaounderé urbain HDs of systematic non-compliers to ivermectin representing around 28% of the study total population and accounting for half of the half of the total infected individuals identified may favour the observed increase in disease prevalence, therefore traducing its recrudescence in certain HDs.

Indeed, the non-compliers to ivermectin treatment microfilaria reservoirs for the population, and this may induce active transmission in this environment colonized by the blackflies. Infectious forms of microfilariaare spread in the community by *Simulium* blackflies during their blood meals from non-complier individuals. In savannah zones such as the Adamaoua region, the spread of these parasitic forms is easier, with the passive movement of blackflies that can reach locations situated tens or even hundreds of kilometres. *Simulium damnosum* s.s, *S. sirbanum* and *S. squamosum* are known as vectors of onchocerciasis in the Adamaoua region [58] (Lamizana, 1981). Although the presence of these vectors in the Adamaoua region needs to be confirmed through recent taxonomic data, we might highlight that these blackfly species exhibit the phenomenon of “limitation” known as one of the factors favoring the transmission of the disease [59,60]. The phenomenon is described as the situation where vectors are efficient even at very low parasite densities [61].

The low intensity of the disease expressed by CMFLs (< 1mf/ss) and the limited number of infected individuals reported in this study do not however suggest active transmission of the parasite in this region Although the decrease in the prevalence of onchocerciasis is noted in the whole region, it should be mentioned that, the local *O. volvulus* parasite population has not yet been reduced to a level below the transmission breakpoint**.** The data from Meiganga of 2013 and 2020 reflect this situation and suggest that the transmission of infection in this HD as in others may evolve in a piecemeal fashion if no special attention is given in delivering ivermectin in the region.

Although the geographical coverage of the CDTI is 100% in the whole Adamaoua region, the surveyed therapeutic coverage rates reveal 6/9 of the region HDs are far below the minimum rate of 65% recommended by the former APOC in the framework of onchocerciasis control as a public health problem. Overall, the surveyed coverage rates studied are lower than those reported by the NOCP over the past 6 years. Those surveyed may be more informative and reflect the status in ivermectin mass treatment within the framework of onchocerciasis control in the Adamaoua region.

Indeed, the therapeutic coverage rates reported by NOCP would be for the most part overestimated at different levels of the recording system used to document ivermectin treatment data for multiple reasons including (1) the justification in the ability to complete the task, (2) the renewal of the confidence of the hierarchy and (3) the hope of obtaining a consequent incentive.  The low surveyed therapeutic coverage rates traducing the low adherence of all age groups of the eligible population, especially men in some HDs and women in others, are key elements to consider for the elimination of the onchocerciasis in this region. This situation calls for additional efforts in delivering ivermectin in the region through increased sensitization of local population and combination with CDTI approach of complementary strategies recently suggested to accelerate the elimination of onchocerciasis in endemic areas [62].

Data reported in this study are close to the threshold of the elimination previously recommended by the former APOC i.e [63] (1) less than 5% prevalence in all surveyed communities and (2) less than 1% in 90% of surveyed communities.  All the 29 communities surveyed had mf prevalence below 5% and 70% had mf prevalence below 1%.

The current profile of the disease suggests low mf prevalences (< 40%) and CMFLs (< 5mf/ss) across the surveyed communities of the Adamaoua region. These data are in agreement with WHO guidelines [64], confirming that onchocerciasis is no longer a public health problem in this region. To better characterize the profile of the disease in the region, it would be advisable to conduct additional surveys in a larger number of communities, with a larger sample size, and above all to carry out OV16 tests to demonstrate children's exposure to the infection, as recommended by the WHO. Onchocerciasis is endemic in all regions of Cameroon and its elimination should start in one region and spread to others. The data reported in this study suggest that the Adamaoua region may be the first region where onchocerciasis elimination should start and be effective in Cameroon. Indeed, the Adamaoua region is known as the water tower of Cameroon, where many rivers especially those infested by blackflies, have their source. Starting the elimination of the disease in this region and extending it to others would be one approach to rid the country of *O. volvulus* infection. The Adamaoua region is geographically the transition zone between the southern and northern regions of Cameroon i.e., the southern forest and the northern savannah areas. The fight against *Onchocerca volvulus* infection in Adamaoua-North and Adamaoua-South directions could be an effective operational intervention plan to be adopted for the future elimination of onchocerciasis in Cameroon.

## 6. Conclusion

The results of this study show a drastic decline in the prevalence of onchocerciasis in the Adamaoua region after 20 consecutive years of CDTI. The disease has reached relatively low levels of endemicity suggesting that the interruption of its transmission is possible in the near future in this part of the country. However, further additional efforts are expected in the population adherence in ivermectin mass treatment to move towards elimination. This goal may be achieved in a very short term between 2025 and 2030 by improving the annual treatment of populations with ivermectin at 85% therapeutic coverage rates. Furthermore, complementary strategies including more in-depth sensitization of community members targeting non-compliers to ivermectin treatment or physical destruction of *Simulium* breeding sites through the community-directed approach (Slash and Clear) would accelerate the elimination of the disease in this part of the country. These data are of great importance to the Ministry of Public Health, in particularly its National Onchocerciasis Control Program, and could serve as a compass for the elimination of onchocerciasis in Cameroon and Central Africa

## 7. References

1. Karam M, Schulz-Key H, Remme J. Population dynamics of Onchocerca volvulus after 7 to 8 years of vector control in West Africa. Acta Trop. 1987; 44:445–57.

2. Habbema JDF, Plaisier AP, van Oortmarssen GJ, Remme J. Prospective evaluation of onchocerciasis control strategies. Acta Leiden 1990; 59:387–398

3. Gumisiriza N, Mubiru F, Siewe Fodjo JN, Mbonye Kayitale M, Hotterbeekx A, Idro R, Makumbi I et al. Prevalence and incidence of nodding syndrome and other forms of epilepsy in onchocerciasis-endemic areas in northern Uganda after the implementation of onchocerciasis control measures. Infect. Dis. Poverty. 2020; 9, 12.

4. Murdoch ME, Hay RJ, Mackenzie CD, Williams JF, Ghalib HW, Cousens S et al. A clinical classification and grading system of the cutaneous changes in onchocerciasis. Br J Dermatol. 1993 129: 260–269

5. Buck AA. Onchocerciasis: Symptomatology, Pathology, Diagnosis. Geneva, World Health Organization, 1974

6. Druet-Cabanac M, Bertocchi I, Preux PM. Onchocerciasis and epilepsy in the north west region of Central African Republic. Afr J Neurol Sci. 1998 ; 17 :37–8

7. Boussinesq M, Pion SDS, Ngangue D, Kamgno J. Relationship between onchocerciasis and epilepsy: a matched case-control study in the Mbam Valley, Republic of Cameroon. Trans R Soc Trop Med Hyg. 2002 ;96 :537–41.

8. Pion SDS, Kaiser C, Boutros-Toni F, Cournil A, Taylor MM, Meredith SEO, et al. Epilepsy in onchocerciasis endemic areas : systematic review and meta-analysis of population-based surveys. PLoS Negl Trop Dis. 2009 ; 3 : e461

9. Korevaar DA, Visser BJ. Reviewing the evidence on nodding syndrome, a mysterious tropical disorder. Int J Infect Dis. 2013 ;17(3) : e149–52.

10. Kaiser C, Pion SD, Boussinesq M. Case-control studies on the relationship between onchocerciasis and epilepsy: systematic review and meta-analysis. PLoS Negl Trop Dis. 2013 ;7(3) : e2147.

11. World Health Organization: Onchocerciasis and its Control. Report of a WHO Expert Committee on Onchocerciasis Control. Geneva: World Health Organization; 1995:1–104.

12. Crosskey RW, Howard TM.  A revised taxonomic and geographical inventory of world blackfl ies (Diptera: Simuliidae), London, The Natural History Museum; 2004.

13. Noma M, Zoure H, Tekle AH, Enyong P, Nwoke BEB, Remme JHF. The geographic distribution of onchocerciasis in the 20 participating countries of the African Programme for Onchocerciasis Control: (1) priority areas for ivermectin treatment. Parasit Vectors 2014 ; 7 :325.

14. Zoure H, Noma M, Tekle AH, Diggle PJ, Giorgi E, Remme JH. The geographic distribution of onchocerciasis in the participating countries of the African Programme for Onchocerciasis Control: (2) pre-control endemicity levels and estimated number infected. Parasit Vectors, 2014; 7:326

15. Boatin B, Molyneux DH, Hougard JM, Christensen OW, Alley ES, Yameogo et al. Patterns of epidemiology and control of onchocerciasis in west Africa. J Helminthol. 1997 Jun ;71(2) :91-101. doi: 10.1017/s0022149x00015741. PMID : 9192715.

16. Kamgno J, Bouchite B, Baldet T, Folefack G, Godin C, Boussinesq M. Contribution à l'étude de la répartition des filarioses humaines dans la Province de l'Ouest du Cameroun. Bull Soc Pathol Exot. 1997 ; 90(5) :327–30.

17. Hougard JM, Alley ES, Yameogo L, Dadzie KY & Boatin BA () Eliminating onchocerciasis after 14 years of vector control : a proved strategy. Journal of Infectious Diseases 2001 ; 184, 497–503.

18. Eckholm, E. Conquering an ancient scourge ; river blindness. N.Y. Times Mag. 1989; Jan 8, 20–

19. Bump JB, Benton B, Seketeli A, Liese, BH. West Africa: river blindness- success in scaling up and lessons learned. scaling up poverty reduction: A Global Learning Process and Conference Shanghai, 2004; May 25–27,

20. Cump & Omura. Ivermectin, 'Wonder drug' from Japan: the human use perspective. Proc. Jpn. Acad., 2011; Ser. B 87.

21. Thylefors B. The Mectizan Donation Program (MDP). Ann Trop Med Parasitol. 2008;102 Suppl 1:39-44.

22. Amazigo U. The African Programme for Onchocerciasis Control (APOC). Ann Trop Med Parasitol. 2008;102 Suppl 1:19-22.

23. Crump A, Morel CM, Omura S The Onchocerciasis Chronicle: From the Beginning to the End? *Trends in Parasitology*, 2012 ; 28 : 9 10.1016/j.pt.2011.10.001

24. Molyneux and Davies. Onchocerciasis control : moving towards the millennium. *Parasitol Today.* 1997 ; 13 : 418-425

25. World Health Organization. African Programme for Onchocerciasis Control (WHO/APOC)15 years of APOC, African Programme for Onchocerciasis Control. Ouagadougou, Burkina Faso. 2011 ; 1995-2010.

26. Plaisier AP, Van Oortmarssen J, Remme J, Habbema JDF. The reproductive lifespan of onchocerca volvulus in West African savanna. Acta Tropica 1991; 48:271–84.

26. Plaisier AP, van Oortmarssen GJ, Remme J, Habbema JDF. The reproductive lifespan of Onchocerca volvulus in West African savanna. Acta Trop. 1991 ; 48, 271–284

27. Lindblade KA, Arana B, Zea-Flores G, Rizzo N, Porter CH, Dominguez A, et al. Elimination of Onchocerca volvulus transmission in the Santa Rosa Focus of Guatemala. Am J Trop Med Hyg. 2007; 77:334–41.

28. Rodríguez-Pérez MA, Fernández-Santos NA, Orozco-Algarra ME, RodríguezAtanacio JA, Domínguez-Vázquez A, Rodríguez-Morales KB, et al. Elimination of onchocerciasis from Mexico. PLoS Negl Trop Dis. 2015 ; 9(7) : e0003922.

29. World Health Organization/Department of Control of Neglected Tropical Diseases. Guidelines for stopping mass drug administration and verifying elimination of human onchocerciasis. Criteria and procedures. 2016; WHO/HTM/NTD/PCT/2016.1.

30. Diawara L, Traore MO, Badji A, Bissan Y, Doumbia K, Goita SF et al. Feasibility of onchocerciasis elimination with ivermectin treatment in endemic foci in Africa: first evidence from studies in mali and senegal. PLoS Negl Trop Dis. 2009; 3(7): e497. 24.

31. Tekle AH, Elhassan E, Isiyaku S, Amazigo UV, Bush S, Noma M et al. Impact of long-term treatment of onchocerciasis with ivermectin in Kaduna State, Nigeria: first

evidence of the potential for elimination in the operational area of the African Programme for Onchocerciasis Control. Parasit Vectors. 2012; 5:28.

32. Traore MO, Sarr MD, Badji A, Bissan Y, Diawara L, Doumbia K et al. Proof-ofprinciple of onchocerciasis elimination with ivermectin treatment in endemic foci in Africa: final results of a study in Mali and Senegal. PLoS Negl Trop Dis. 2012; 6(9): e1825.

33. Higazi TB, Zarroug IM, Mohamed HA, Elmubark WA, Deran TC, Aziz N, et al. Interruption of Onchocerca volvulus transmission in the Abu Hamed focus, Sudan. Am J Trop Med Hyg. 2013 Jul ;89(1):51-7. doi: 10.4269/ajtmh.13-0112. Epub 2013 May 20. PMID : 23690554 ; PMCID : PMC3748488.

34. Katabarwa M, Lakwo T, Habomugisha P, Agunyo S, Byamukama E, Oguttu D,et al. Transmission of Onchocerca volvulus by Simulium neavei in Mount Elgon focus of Eastern Uganda has been interrupted. Am J Trop Med Hyg. 2014 Jun ;90(6) :1159-66. doi: 10.4269/ajtmh.13-0501. Epub 2014 Mar 31. PMID : 24686740 ; PMCID : PMC4047747.

35. Lakwo TL, Garms R, Rubaale T, Katabarwa M, Walsh F, Habomugisha P et al. The disappearance of onchocerciasis from the Itwara focus, Western Uganda after elimination of the vector Simulium neavei and 19 years of annual ivermectin treatments. Acta Trop, 2013; 126: 218–221. doi: 10.1016/j.actatropica .02. 016 PMID: 23458325

36. Mackenzie CD, Homeida MM, Hopkins AD and Lawrence JC. Elimination of onchocerciasis from Africa: possible? Trends in Parasitology Jan 2012, Vol. 28, No. 1 doi :10.1016/j.pt.2011.10.003

37. Tekle AH, Zouré HGM, Noma M, et al. Progress towards onchocerciasis elimination in the participating countries of the African program for onchocerciasis control : epidemiological evaluation results. *Infectious Diseases of Poverty*. 2016 ;5 :66

38. Katabarwa MN, Eyamba A, Nwane P, Enyong P, Yaya S, Baldiagaï J et al. Seventeen years of annual distribution of ivermectin has not interrupted onchocerciasis transmission in North Region, Cameroon. Am J Trop Med Hyg. 2011 Dec; 85(6) :1041-9. doi: 10.4269/ajtmh.2011.11-0333. PMID : 22144441 ; PMCID : PMC3225149.

39. Kamga GR, Dissak-Delon FN, Nana-Djeunga HC, Biholong BD, Ghogomu SM, Souopgui J et al. Important progress towards elimination of onchocerciasis in the West Region of Cameroon. Parasit Vectors. 2017 Aug 3 ;10(1) :373. doi: 10.1186/s13071-017-2301-7. PMID : 28774318 ; PMCID : PMC5543544.

40. Génieux M. Climatologie Du Cameroun, in : Atlas Du Cameroun. ORSTOM, 1958 ; France.

41. Olivry JC. Fleuves et rivières du Cameroun. Monographie hydrologique. ORSTOM, 1986 ; Paris, p. 733.

42. Suchel JB. Les climats du Cameroun. Ph.D Thesis University of Saint-Etienne, 1998; France.

43. Letouzey R. Observation phytogéographique concernant le plateau africain de l'Adamaoua. Adansonia, sect. B, 1969 ; 29 (3) : 321-337.

44. Letouzey R. Notice de la carte phytogéographique du Cameroun à 1/500 000. Vol. IS-S : Domaine sahélien et soudanien. IRA, Cameroun, lnstitut de la Carte Internationale de la Végétation, 1985 (Toulouse/France), 26 p.

45. Paris Frédéric. Chronique d'une endémie opportuniste. Développement rural et onchocercose au Nord Cameroun (Foyer du Faro). In : Espace, populations, sociétés, 2000-2. Maladies émergentes et reviviscentes. 1944 ; pp. 241-252 ; doi: https://doi.org/10.3406/espos.2000.1944

46. Collins RC, Brandling-Bennett AD, Hollman RB, Campbell CC, Darsie RF. Parasitological diagnosis of onchocerciasis: comparison of incubation media and incubation times for skin snips. Am J Trop Med Hyg. 1980; 29(1):35–41.

47. Coffeng LE, Pion SDS, O'Hanlon S, Cousens S, Abiose AO, Fischer PU et al. Onchocerciasis: The Pre-control Association between Prevalence of Palpable Nodules and Skin Microfilariae. PLoS Negl Trop Dis, 2013 ; 7(4): e2168. doi: 10.1371/journal.pntd.0002168

48. Hoerauf A, Mand S, Adjei O, Fleischer B, Büttner DW. Depletion of *Wolbachia* endobacteria in *Onchocerca volvulus* by doxycycline and microfilaridermia after ivermectin treatment. *Lancet.* 2001 ; 357 :1415–1416. doi: 10.1016/S0140-6736(00)04581-5.

49. McMahon JE, Sowa SIC, Maude GH, Kirkwood BR. Onchocerciasis in Sierra Leone 2: a comparison of forest and savanna villages. Transactions of the Royal Society of Tropical Medicine and Hygiene, 1988 ; 82 (4) 595-600.

50. Prost A, Hervouet JP, Thylefors B. Epidemiologic status of onchocerciasis. Bull World Health Organ. 1979; 57: 655–62

51. Walker M, Stolk WA, Dixon MA, Bottomley C, Diawara L, Traoré MO et al. Modelling the elimination of river blindness using long-term epidemiological and programmatic data from Mali and Senegal. Epidemics. 2017 Mar ;18 :4-15. doi : 10.1016/j.epidem.2017.02.005. PMID : 28279455 ; PMCID : PMC5340858.

52. Remme J, Ba O, Dadzie KY, Karam M. A force-of-infection model for onchocerciasis and its applications in the epidemiological evaluation of the Onchocerciasis Control Programme in the Volta River basin area. Bull World Health Organ. 1986; 64:667–81.

53. Kamgno J, Boussinesq M. Hyperendémicité de la loase dans la plaine Tikar, région de savane arbustive du Cameroun. *Bulletin de la Société de Pathologie Exotique*, 2001, 94 (4), p. 342-346. ISSN 0037-9085.
*Identifiant IRD* :

54. Noma M, Nwoke BEB, Nutall I, Tambala PA, Enyong P, Namsenmo A. Rapid epidemiological mapping of onchocerciasis (REMO): its application by the African Programme for Onchocerciasis Control (APOC), Annals of Tropical Medicine & Parasitology, 2002; 96:sup1, S29-S39, DOI: 10.1179/000349802125000637

55. Tsapi EM, Togjom FG, Gamago G-A, Pone JW, Teukeng FFD. Prevalence of onchocerciasis after seven years of continuous community-directed treatment with ivermectin in the Ntui health district Centre region Cameroon. Pan Afr Med J. 2020 ;1 :180.

56. Aza'ah RA, Sumo L, Ntonifor NH, Bopda J, Bamou RH, Nana-Djeunga HC. Point prevalence mapping reveals hotspot for onchocerciasis transmission in the Ndikinimeki Health District, Centre Region, Cameroon. Parasit Vectors. 2020 Oct 16 ;13(1) :519. doi: 10.1186/s13071-020-04387-6. PMID : 33066826 ; PMCID : PMC7565768.

57. Forrer A, Wanji S, Obie ED, Nji TM, Hamill L, Ozano K et al. Why onchocerciasis transmission persists after 15 annual ivermectin mass drug administrations in South-West Cameroon. BMJ Glob Health. 2021 Jan ;6(1) : e003248. doi: 10.1136/bmjgh-2020-003248. PMID : 33431378 ; PMCID : PMC7802695.

58. Traore-Lamizana, Monographie sur la cartographie des gîtes larvaires des vecteurs d'onchocercose au Cameroun, p. 1, Carte montrant la distribution des gîtes larvaires de Simulium sp. Document de l'IRD, 1981.

59. Philippon B. Etude de la transmission d'Onchocerca volvulus (Leuckart, 1893) (Nematoda, Onchocercidae) par Sirnuliurn dainriosuin Theobald, 1903 (Diptera, Simuliidae) en Afrique tropicale. Travaux et Documents de l'O.R.S.T.O.M. No. 63. Paris : Office de la Recherche Scientifique et Technique d'Outre-Mer. ORSTOM, France ans; 1977.

60. Demanou M, Enyong P, Pion SD, Basanez MG, Boussinesq M. Experimental studies on the transmission of Onchocerca volvulus by its vector in the sanaga valley (Cameroon): simulium squamosum B. Intake of microfilariae and their migration to the haemocoel of the vector. Ann Trop Med Parasitol. 2003;97(4):381–402.

61. Amuzu H, Wilson MD, Boakye DA. Studies of Anopheles gambiae sl (Diptera: Culicidae) exhibiting different vectorial capacities in lymphatic filariasis transmission in the Gomoa district, Ghana. Parasit Vectors. 2010 ;3 :85.

62. Boussinesq M, Fobi G and Kuesel AC, 2017. Alternative treatment strategies to accelerate the elimination of onchocerciasis. Int Health 2018; 10 : i40–i48 doi:10.1093/inthealth/ihx054

63. African Program for Onchocerciasis Control/World Health Organization. Conceptual and operational framework of onchocerciasis elimination with

ivermectin treatment. World Health Organization/African Program for Onchocerciasis Control, 2010. WHO/APOC/MG/10.1, JAF 16.6 II.

64. World Health Organization. Report of the external mid-term evaluation of the African Programme for Onchocerciasis Control; JAF 16.8. 2010. http://who.int/apoc/MidtermEvaluation_29Oct2010_final_printed.pdf. Accessed 21 Mar 2018.

## Declaration

### Ethics approval and consent to participate

The study protocol was approved by the Cameroon National Ethics Committee (N◦2020/08/1286/CE/CNERSH/SP), on 21 August 2020. Each study participant aged 18 and over personally affixed both thumbprints to the informed consent form presented to them to confirm their participation in the study. Parental consent was obtained for children aged 5 to

### Competing interests

The authors declare that they have no competing interests.

### Funding

This study was funded by International Eye Foundation (IEF), a Non-Governmental Development Organisation (NGDO) which supports Ministry of Health in the fight against onchocerciasis in the Adamaoua Region.

### Authors' contribution

AD, HCND, MB, FN, SP and JK designed the study protocol. AD, PBN, GRN collected data on the field. AD, HCND and GRN provided and monitored data collect and management. HCND and PBN reviewed the results and contributed to the interpretation of data. AD and HCND conducted data analysis and prepared the first draft of the manuscript. All authors participated in the interpretation of results and revisions of the manuscript. All authors read

and approved the final manuscript.

## Authors' contribution

PN, HND and JK designed the study protocol. PN, NZT, AD, GRN, MT, JB, SB, AB, AN, HN, PM, GM collected data on the field. PN, HND and YEN supervised and monitored data collection and management. PN, HND and JK reviewed the results and contributed to their interpretation. PN, FNB and YEN conducted data analysis, PN a prepared the first draft of the manuscript. All authors participated in the interpretation of results and revisions of the manuscript. All authors read and approved the final manuscript.

## Acknowledgments

The authors are grateful to the Regional Health Delegate of Adamaoua region, all health staff at District and health area levels, the traditional chiefs and their respective populations for their active involvement for data collection in the field.

## Authors' information

PN, Email: philino07@yahoo.fr, Medical Entomologist, Research Scientist at the High Institute of Scientific and Medical Research (ISM) Yaoundé Cameroon.

HND, Email: nanadjeunga@crfilmt.org, Molecular Parasitologist, at the High Institute of Scientific and Medical Research (ISM) Yaoundé Cameroon

AD, Email: domche_andre@yahoo.fr, Medical Entomologist, Ph.D student at the University of Yaoundé I/Research assistant at the High Institute of Scientific and Medical Research (ISM) Yaoundé Cameroon.

GRN, Email: njitchouang@yahoo.fr, Biochemist, at the High Institute of Scientific and Medical Research (ISM) Yaoundé Cameroon.

JK, Email: kamgno@crfilmt.org, Epidemiologist, Director of the High Institute of Scientific and Medical Research (ISM) Yaoundé Cameroon

YEN: Email: emalio2002@yahoo.fr, Statistician at the High Institute of Scientific and Medical Research (ISM) Yaoundé Cameroon

NZT: Email: narcissenzunetoche@yahoo.fr, Biochemist, Ph.D student at the University of Yaoundé I/Research assistant at the High Institute of Scientific and Medical Research (ISM) Yaoundé Cameroon.

MT: Email: augustaflore@yahoo.fr, Ph.D student at the University of Buea I/Research assistant at the High Institute of Scientific and Medical Research (ISM) Yaoundé Cameroon.

JB: f_bopda@yahoo.fr, laboratory technician at the High Institute of Scientific and Medical Research (ISM) Yaoundé Cameroon.

FNB: Email: fesuhbe@yahoo.co.uk,

SB: Email: mbickmensteve@yahoo.fr; laboratory technician at the High Institute of Scientific and Medical Research (ISM) Yaoundé Cameroon

AB: Email: aubinsantos1988@yahoo.fr; laboratory technician at the High Institute of Scientific and Medical Research (ISM) Yaoundé Cameroon

AN: Email: nkouelle@yahoo.fr; Project Manager at International Eye Foundation

HO: Email: obamahonore@yahoo.fr, Data manager at the National Onchocerciasis Control Program/ Ministry of Public Health

PM: Email: mepafel2001@yahoo.fr, Data manager at the National Onchocerciasis Control Program/ Ministry of Public Health

GNA: Email gb_nko@yahoo.fr, Head of Department of Neglected Tropical Diseases/ Ministry of Public Health

BDB: biholong_di@yahoo.fr; Permanent secretary of National Onchocercisasis Control Program

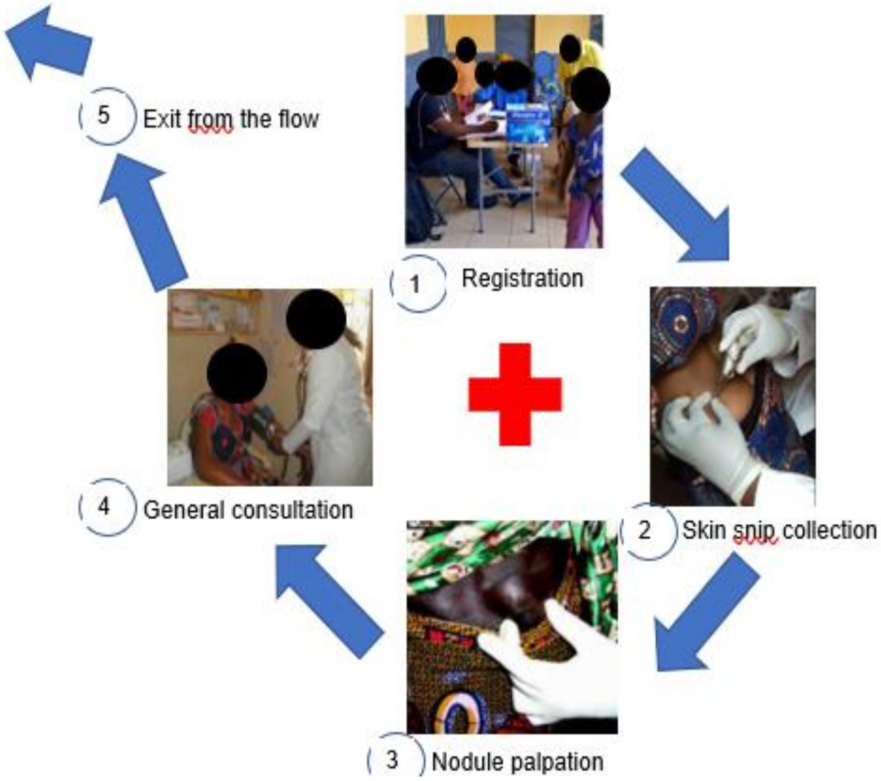


**Figure 1**. Participant progress flow during the data collection procedure

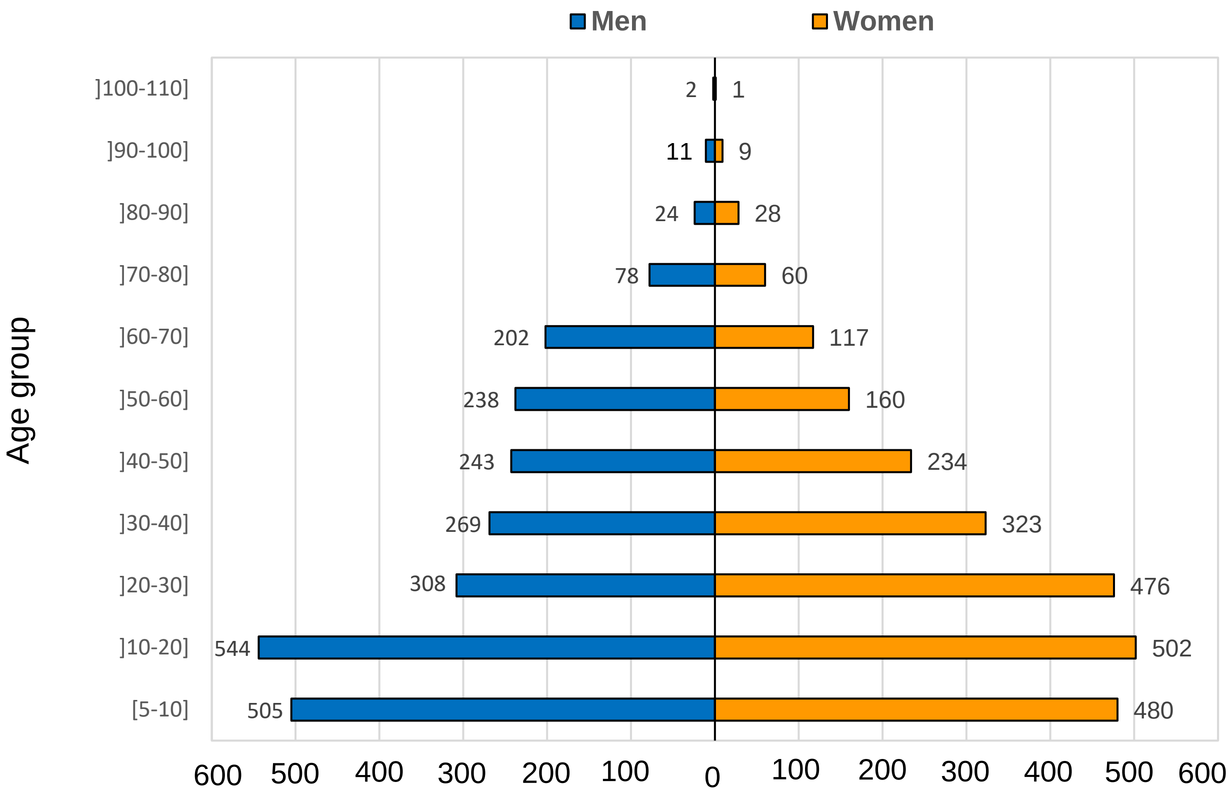


**Figure 2** : Distribution of the study population according to gender and age groups

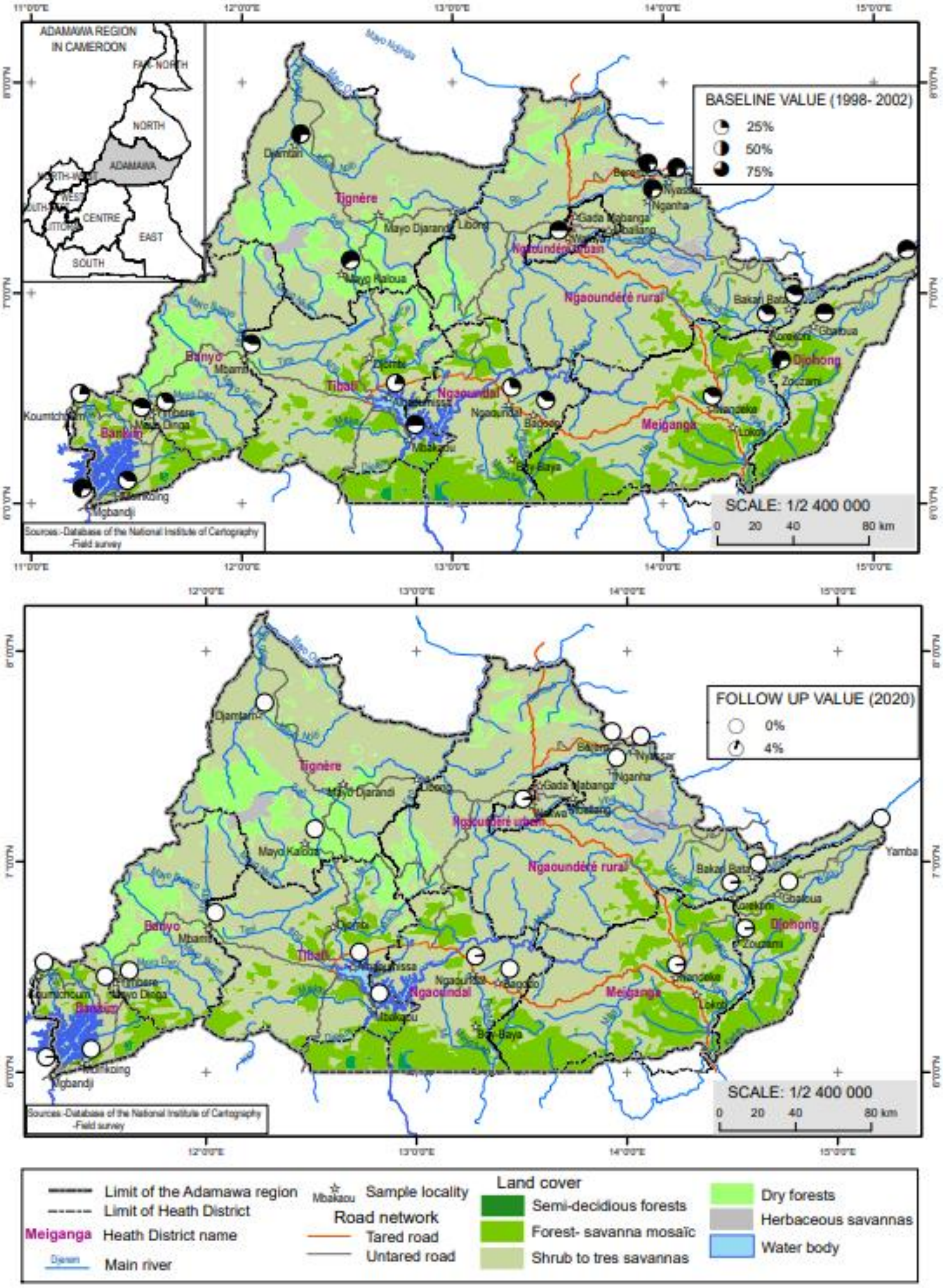


**Figure 3 :** Trend in microfilaria prevalence in 22 communities surveyed in the 1998-2002 baseline and the 2020 follow-up surveys in the Adamaoua region

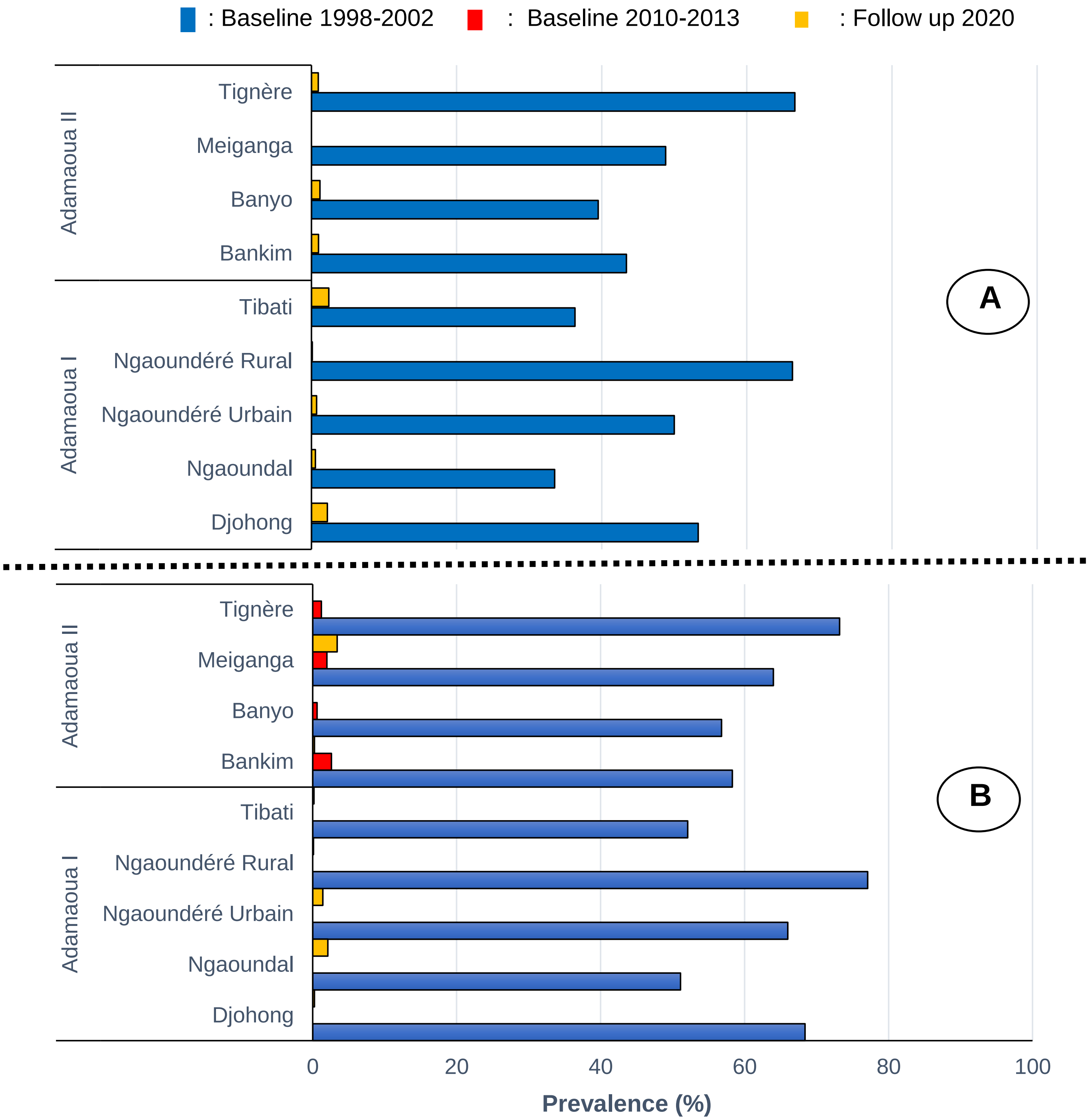


**Figure 4** : Nodule (**A**) and microfilaria (**B**) prevalences recorded in surveyed Health Districts

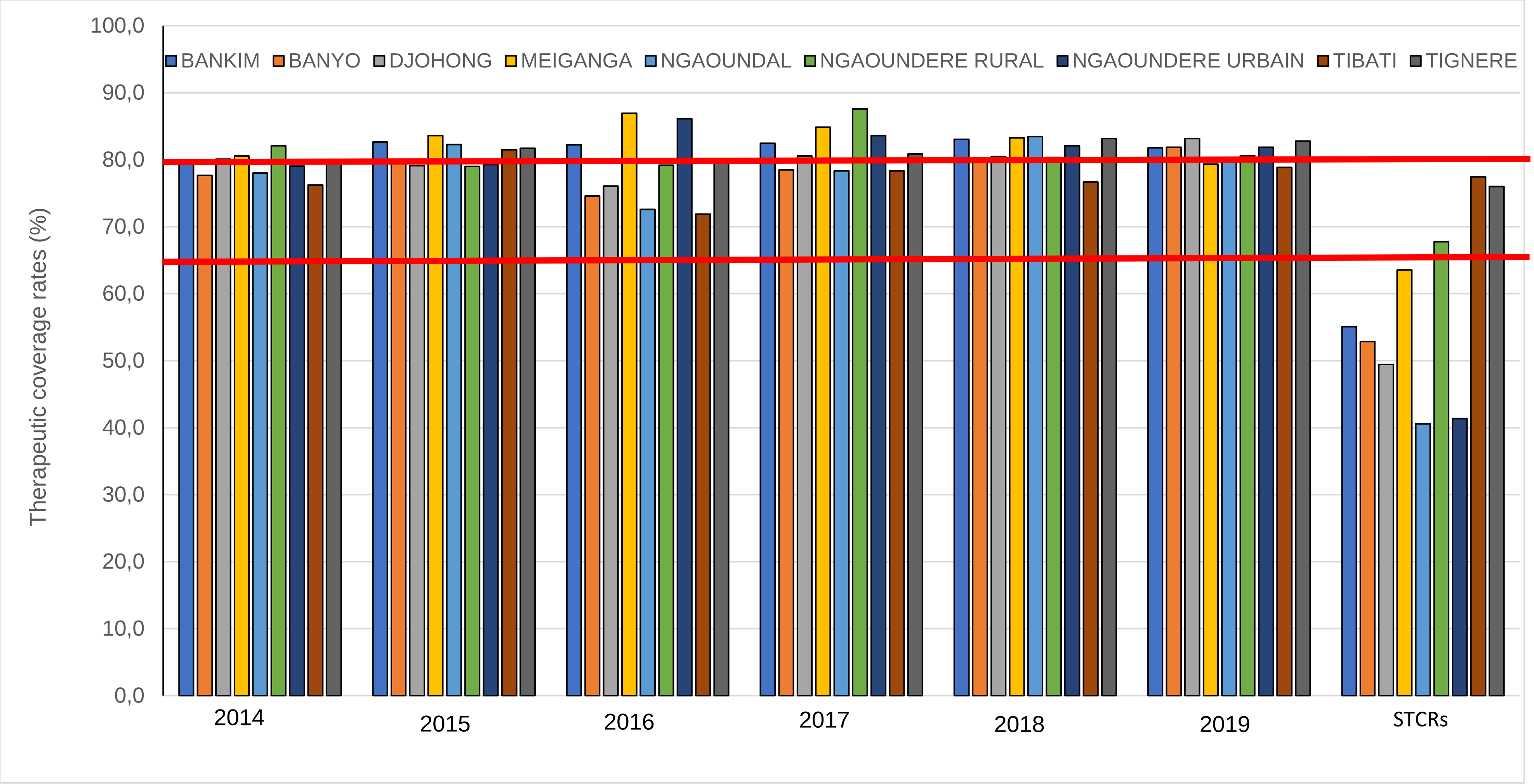


**Figure 5** : Ivermectin therapeutic coverage rates reported and surveyed between 2014 and 2019 in the nine Health Districts of Adamaoua Region

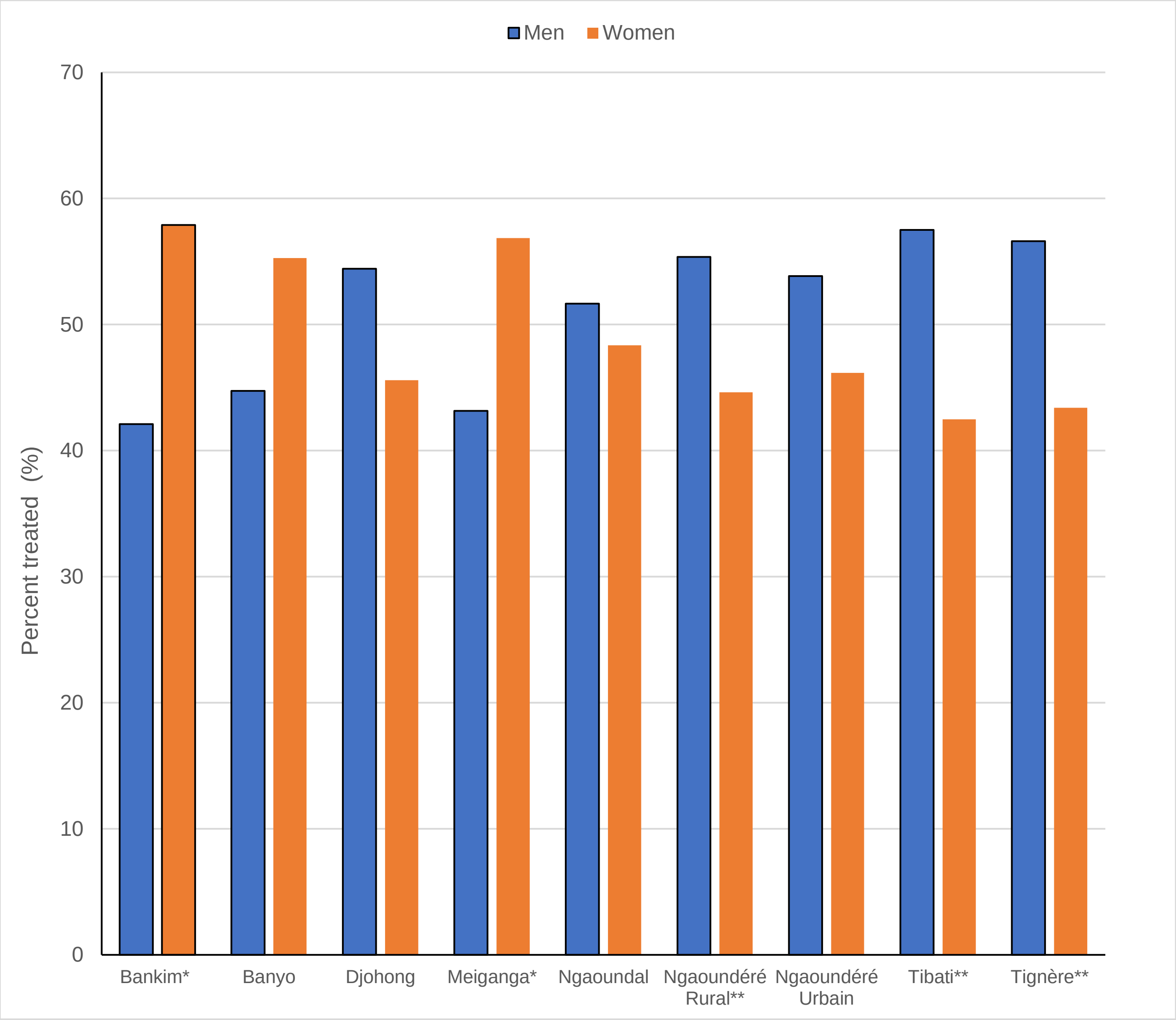


**Figure 6** : Surveyed participation rate according to gender in the 2019 mass ivermectin treatment in the 9 health districts of the Adamaoua region

* significantly higher adherence among women than men
** significantly higher uptake by men than by women

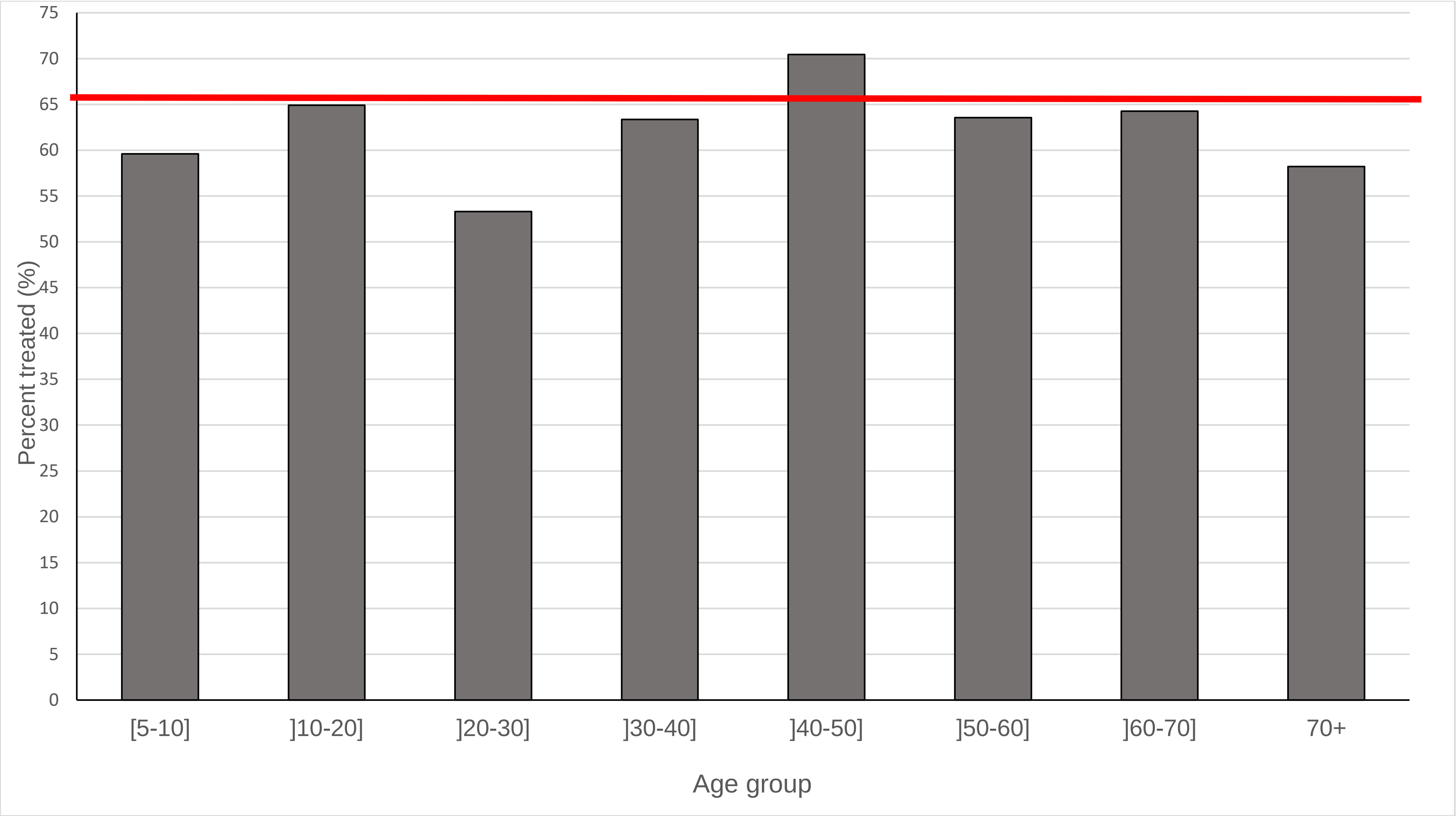


**Figure 7** : Surveyed participation rate according to age groups in the 2019 mass ivermectin treatment in the 9 health districts of the Adamaoua region

 Click here to access/download;Table;Tables_Adamaoua.pdf

**Table 1** : Mf prevalences in communities prospected during the 1998-2002 and 2020 epidemiological surveys conducted in Adamaoua region.

| | | Geographic coordinates | | Baseline survey (1998-2002) | | Follow up survey (2020) | | | |
|---|---|---|---|---|---|---|---|---|---|
| **Health District** | **Community** | **Latitude** | **Longitude** | **# of persons examined** | **Mf prevalence** | **# of persons examined** | **Mf preva-lence [95%IC]** | **CMFL (All)** | **CMFL (+20yrs)** |
| Bankim | Mgbandji* | 5°59'22.5'' | 11°16'36.5'' | 30 | 80.2 | 87 | 1.14 [0.06 - 7.13] | 0.023 | 0.035 |
| | Koumtchoum* | 6°26'81.8'' | 11°12'14.8'' | 30 | 38.9 | 227 | 0 | - | - |
| | Moinkoing* | 6°02'58.3'' | 11°24'40.2'' | 32 | 55.7 | 100 | 0 | - | - |
| Banyo | Mayo Dinga* | 6°25'18.2'' | 11°34'15.0'' | 30 | 60.3 | 173 | 0 | - | - |
| | Mbamti* | 6°41'02.7'' | 12°04'00.0'' | 32 | 59.3 | 180 | 0 | - | - |
| | Yimbere* | 6°27'35.3'' | 11°34'269'' | 30 | 50.9 | 239 | 0 | - | - |
| Djohong | Gbatoua* | 6°50'42.6" | 14°42'56.4" | 33 | 65.7 | 146 | 0.68 [0.03 - 4.32] | 0.008 | 0.013 |
| | Yamba* | 7°06'21.0" | 15°12'20.8" | 30 | 71.0 | 129 | 0 | - | - |
| Meiganga | Korekoni* | 6°08'09.3" | 14°33'48.0" | 33 | 55.4 | 225 | 4.00 [1.96 - 7.70] | 0.127 | 0.051 |
| | Lokoti | 6°22'00.0" | 14°20'00.0" | 43 | - | 89 | 1.12 [0.05 - 6.97] | 0.022 | 0.022 |
| | Zouzami* | 6°37'08.3" | 14°32'20.0" | 30 | 80.3 | 168 | 2.97 [1.10 - 7.17] | 0.049 | 0.071 |
| | Nandeke* | 6°27'09.7" | 14°13'47.1" | 38 | 56.3 | 138 | 4.34 [1.77 - 9.63] | 0.129 | 0.047 |
| Ngaoundal | Boy-Baya | 6°25'26.4" | 13°23'10.7" | 31 | - | 56 | 3.57 [0.62 - 13.38] | 0.048 | 0.048 |
| | Bagodo* | 6°25'26.4" | 13°23'10.7" | 50 | 55.6 | 110 | 0 | - | - |
| | Ngaoundal* | 6°28'06.0" | 13°16'09.4" | 31 | 46.6 | 206 | 2.91 [1.18 - 6.52] | 0.050 | 0.050 |
| Ngaoundere Rural | Nganha* | 7°26'08.8" | 13°55'43.2" | 30 | 80.2 | 169 | 0.59 [0.03 - 3.75] | 0.010 | 0.010 |
| | Bakari Bata* | 6°55'41.9" | 14°35'51.7" | 33 | 59.1 | 213 | 0 | - | - |
| | Berem* | 7°33'03.7'' | 13°55'28.4'' | 30 | 86.7 | 299 | 0 | - | - |
| | Nyassar* | 7°31'40.1'' | 14°01''43.7'' | 30 | 82.5 | 281 | 0 | - | - |
| Ngaoundere Urbain | Wakwa* | 7°16'05.8'' | 13°33'01.6'' | 30 | 66.0 | 95 | 4.21 [1.35 - 11.03] | 0.026 | 0.051 |
| | Gadamabanga | 7°21'37.7" | 13°35'05.5" | 32 | - | 78 | 0 | - | - |
| | Mballang | 7°18'00.8'' | 13°44'30.2'' | 30 | - | 110 | 0 | - | - |
| Tibati | Mbakaou* | 6°18'13.9'' | 12°47'48,0'' | 52 | 67.4 | 197 | 0.50 [0.02 - 3.23] | 0.006 | 0 |
| | Alhamissa* | 6°30'01,9'' | 12°41'58.9'' | 30 | 36.7 | 113 | 0 | - | - |
| | Djombi | 6°41'28.8'' | 12°36'18.4'' | 33 | - | 240 | 0 | - | - |
| Tignere | Libong | 7°23'21.5'' | 13°00'06.4'' | 30 | - | 171 | 0 | - | - |
| | Mayo-Djarandi | 7°22'05.0'' | 12°38'59.3'' | 30 | - | 175 | 0 | - | - |
| | Mayo-Kaloua* | 7°05'13.7'' | 12°28'15.0'' | 23 | 74.1 | 187 | 0 | - | - |
| | Djamtari* | 7°42'03.4'' | 12°15'01.9'' | 36 | 87.7 | 213 | 0 | - | - |

*Communty prospected in 1998-2002 and 2020

**Table 2** : Microfilaria and nodule prevalences recorded in the health districts of the Adamaoua Region

| Health District | N | Prevalence | |
|---|---|---|---|
| | | Nodule | Mf |
| Bankim | 414 | 0.96 [0.31-2.63] | 0.24 [0.01-1.55] |
| Banyo | 592 | 1.18 [0.52-2.53] | 0.00 [0.00-0.80] |
| Djohong | 275 | 2.18 [0.89-4.92] | 0.36 [0.02-2.33] |
| Meiganga | 620 | 0.00 [0.00-0.77] | 3.40 [2.16-5.23] |
| Ngaoundal | 372 | 0.53 [0.09-2.14] | 2.15 [1.00-4.36] |
| Ngaoundere rural | 962 | 0.10 [0.00-0.67] | 0.10 [0.00-0.67] |
| Ngaoundere urbain | 283 | 0.71 [0.12-2.81] | 1.41 [0.45-3.82] |
| Tibati | 550 | 2.36 [1.32-4.11] | 0.18 [0.01-1.17] |
| Tignere | 764 | 0.94 [0.41-2.01] | 0.00 [0.00-0.64) |
| **TOTAL** | **4,814** | **0.87 [0.64-1.19]** | **0.77 [0.54-1.07**] |
