# Supplementary material for: Status of human onchocerciasis transmission in the Adamaoua region of Cameroon after 20 years of ivermectin mass distribution
Source: PLoS Negl Trop Dis. 2025 Mar 4;19(3):e0011511. doi: 10.1371/journal.pntd.0011511 (PMC11925462; doi:10.1371/journal.pntd.0011511)
Supplement: Attachment — Submitted filename: PNTD-D-23-00854_R1_reviewer_RN.pdf [file pntd.0011511.s009.pdf]

PLOS Neglected Tropical Diseases

Status of human oncherciasis transmission in the Adamaoua region of Cameroon after 20 years of ivermectin mass distribution

--Manuscript Draft--

**Manuscript Number:**	PNTD-D-23-00854R1
**Full Title:**	Status of human oncherciasis transmission in the Adamaoua region of Cameroon after 20 years of ivermectin mass distribution
**Short Title:**	Current status of onchocerciasis in Adamaoua Region of Cameroon
**Article Type:**	Research Article
**Keywords:**	Onchocerciasis - Ivermectin - CDTI - Elimination - Adamaoua Region - Cameroon.
**Abstract:**	Introduction<br>~~Although evidences of onchocerciasis elimination through community directed treatment with ivermectin (CDTI) are reported in some foci in West and East Africa, in Central Africa the situation does not sound promising, despite two decades treatment in some foci of the sub-region.~~ This paper describes the current situation of onchocerciasis in Adamaoua Region of Cameroon after 20 consecutive years of ivermectin mass treatment.<br>Material and methods The study was conducted between August and September 2020 in Adamaoua Region of Cameroon. Onchocerciasis endemicity was assessed through parasitological and clinical diagnosis. Microfilarodermia and nodule prevalences assessed in 2020 were compared to those of 1998-2002 and 2010-2013 surveys ~~using ANOVA and Welch's unequal variances t-test.~~<br>Results<br><br>A total of 4,814 participants aged between 5 and 108 years, including 50.4% men and 49.6% women ~~with a sex ratio of 1:1.~~ For ~~the whole region~~ the nodule and microfilaria prevalence were 0.87 [0.64 - 1.19] % and 0.77 [0.54 - 1.07] % respectively. At the community level, the mf prevalences ranged from 0.5% to 4.5%. Globally, the community microfilarial loads (CMFL) ~~were~~ < 0.5 mf/ss. The survey~~ed~~ therapeutic coverage rates were between 40% and 78% ~~but~~ lower than those reported (79% - 83%). The adherence rates in ivermectin treatment of all age groups of the population were below 65%, except for the 40-50 age group where it was ≈70%.<br>Conclusion The results of this study show a drastic decline in onchocerciasis prevalences after 20 consecutive years of CDTI, indicating a significant progress towards the elimination of the disease in Adamaoua Region. However, additional efforts are needed to increase the population adherence in ivermectin treatment to achieve the elimination of the disease in this Region. ~~These data are useful for the Ministry of health in guiding the elimination procedure of onchocerciasis in Cameroon.~~
**Additional Information:**	
**Question**	**Response**
**Government Employee**<br><br>Are you or any of the contributing authors an employee of the United States government?<br><br>Manuscripts authored by one or more US Government employees are not copyrighted, but are licensed under a CC0 Public Domain Dedication, which allows	No - No authors are employees of the U.S. government.

Powered by Editorial Manager® and ProduXion Manager® from Aries Systems Corporation

unlimited distribution and reuse of the article for any lawful purpose. This is a legal requirement for US Government employees.<br><br>This will be typeset if the manuscript is accepted for publication.	
**Financial Disclosure**<br><br>Enter a financial disclosure statement that describes the sources of funding for the work included in this submission. Review the submission guidelines for detailed requirements. View published research articles from *PLOS Neglected Tropical Diseases* for specific examples.<br><br>This statement is required for submission and **will appear in the published article** if the submission is accepted. Please make sure it is accurate.<br><br>**Funded studies**<br>Enter a statement with the following details:<br>• Initials of the authors who received each award<br>• Grant numbers awarded to each author<br>• The full name of each funder<br>• URL of each funder website<br>• Did the sponsors or funders play any role in the study design, data collection and analysis, decision to publish, or preparation of the manuscript?<br><br>Did you receive funding for this work?	The author(s) received no specific funding for this work.
**Competing Interests**<br><br>On behalf of all authors, disclose any competing interests that could be perceived to bias this work.<br><br>This statement will be typeset if the manuscript is accepted for publication.	No competing interests

Review the instructions link below and PLOS NTDs' competing interests policy to determine what information must be disclosed at submission.	
**Data Availability**<br><br>Provide a **Data Availability Statement** in the box below. This statement should detail where the data used in this submission can be accessed. This statement will be typeset if the manuscript is accepted for publication.<br><br>Before publication, authors are required to make all data underlying their findings fully available, without restriction. Review our PLOS Data Policy page for detailed information on this policy. Instructions for writing your Data Availability statement can be accessed via the Instructions link below.	~~The data we have presented in this paper may be free of access to the general public and should not be restricted. They should be accessible on the Internet without any particular requirements, as they will help to inform the scientific community about the situation of onchocerciasis in the Adamaoua region.~~ All data are in the manuscript and/or supporting information files

Powered by Editorial Manager® and ProduXion Manager® from Aries Systems Corporation

Manuscript

Click here to access/download;Manuscript;Manuscript_2024_Rev.docx

Status of human oncherciasis transmission in the Adamaoua region of Cameroon after 20 years of ivermectin mass distribution

Philippe Nwane[*1,2], Hugues Clotaire Nana-Djeunga[1], Narcisse Nzune Toche[1], André Domché[1,2], Fesuh Nono Bertrand[3], Yannick Emalio Niamsi[1], Guy Roger Njitchuang[1], Martine Augusta Flore Tsasse[1], Jean Bopda[1], Steve Bickmen[1], Aubin Balog[1], Alexis Nkwelle[4], Patrice Nkwelle[4†], Clarisse Ebene[5], Honoré Obama[5], Paul Messi[5], Benjamin Biholong[5], Serge Billong[5], Georges Nko'o Ayissi[6] and Joseph Kamgno[1,7]

Author details

[1]Higher institute of scientific and medical research (ISM), P.O. Box 5797 Yaoundé, Cameroon ;
[2]Laboratory of General Biology, University of Yaounde I, P.O. Box 337, Yaoundé, Cameroon
[3]National Advanced School of Engineering, University of Yaoundé I, Yaoundé, Cameroon
[4]International eye Foundation, Cameroon ;
[5]National Onchocerciasis Control Program/Ministry of Public Health, Yaounde, Cameroon ;
[6]Departement of Neglected Tropical Diseases Department/Ministry of Public Health, Yaounde, Cameroon ;
[7]Faculty of Medicine and Biomedical Sciences, University of Yaounde I, Yaounde, Cameroon.

† Deceased some months after the data analysis.

ABSTRACT

Introduction
Although evidences of onchocerciasis elimination through community directed treatment with ivermectin (CDTI) are reported in some foci in West and East Africa, in Central Africa the situation does not sound promising, despite two decades treatment in some foci of the sub-region. This paper describes the current situation of onchocerciasis in Adamaoua Region of Cameroon after 20 consecutive years of ivermectin mass treatment.

Material and methods
The study was conducted between August and September 2020 in Adamaoua Region of Cameroon. Onchocerciasis endemicity was assessed through parasitological and clinical diagnosis. Microfilarodermia and nodule prevalences assessed in 2020 were compared to those of 1998-2002 and 2010-2013 surveys using ANOVA and Welch's unequal variances t-test.

Results

A total of 4,814 participants aged between 5 and 108 years, including 50.4% men and 49.6% women with a sex ratio of 1:1. For the whole region the nodule and microfilaria prevalence were 0.87 [0.64 - 1.19] % and 0.77 [0.54 - 1.07] % respectively. At the community level, the mf prevalences ranged from 0.5% to 4.5%. Globally, the community microfilarial loads (CMFL) were < 0.5 mf/ss. The surveyed therapeutic coverage rates were between 40% and 78% but lower than those reported (79% - 83%). The adherence rates in ivermectin treatment of all age groups of the population were below 65%, except for the 40-50 age group where it was ≈70%.

Conclusion
The results of this study show a drastic decline in onchocerciasis prevalences after 20 consecutive years of CDTI, indicating a significant progress towards the elimination of the disease in Adamaoua Region. However, additional efforts are needed to increase the population adherence in ivermectin treatment to achieve the elimination of the disease in this Region. These data are useful for the Ministry of health in guiding the elimination procedure of onchocerciasis in Cameroon.

Keywords: Onchocerciasis - Ivermectin - CDTI - Elimination - Adamaoua Region - Cameroon.

AUTHOR SUMMARY

Onchocerciasis control relies on mass treatment of populations with an annual dose of ivermectin. It was originally assumed that an onchocerciasis-endemic community using the community-directed treatment with ivermectin (CDTI) approach should become free of the disease after 15 years, a period corresponding to the maximum lifespan of the adult worm in humans. This approach has contributed to eliminate onchocerciasis in few foci in West Africa (e.g : Mali, Niger, Senegal and Nigeria), and East Africa (e.g Uganda). In Central Africa, elimination of this disease remains uncertain after 20 consecutive years of mass treatment in certain foci. The data presented in this study are very promising, showing that elimination of the disease through the CDTI approach may be possible in Adamaoua Region of Cameroon. Achieving the goal of elimination of onchocerciasis in this part of the country in the near future requires little efforts of the population in adhering to ivermectin treatment. This will involve raising awareness among men and women of all eligible age groups for ivermectin treatment.

1. INTRODUCTION

Also known as "*River Blindness*"; onchocerciasis is caused by a parasitic worm *Onchocerca volvulus* transmitted from person to person through bites of blackflies belonging to the genus *Simulium*. Over the course of few months to one year, the transmitted L_3 infective stages of the parasite molt twice and develop into sexually mature adult. Female worms live in fibrous nodules of the skin where they remain sessile, while males migrate between the nodules to mate with settled females. Both female and male adult worms live between 2 and 15 years with an average life expectancy of 10 years [1, 2]. During this period, ~~patent~~ females release between 700 and 1,500 microfilariae (mf) per day [1,3] which predominantly live in the skin for 3 to 5 years causing lesions including intense itching, thickening, skin depigmentation,

and loss of its elasticity. Other clinical manifestations such as hanging groin, skin atrophy, papular onchodermatitis and chronic papular onchodermatitis, lichenified onchodermatitis are commonly reported [4]. The microfilaria (mf) can migrate throughout the intercellular fluid and reach the eyes causing ocular manifestations which can lead to irreversible blindness [5]. Although the physiopathology is not well known, several studies have mentioned links between onchocerciasis, epilepsy and nodding syndrome [3, 6, 7, 8, 9, 10].

Onchocerciasis is distributed in the world with most of cases recorded in Sub-Saharan Africa (SSA) [11,12,13,14]. Historical data from SSA show a bleak picture of this disease in several endemic villages where prevalences of infected individuals sometimes exceeded 80%, and the rates of onchocercal blindness around 10% [15, 16, 17]. In regard with the gravity of this severe infectious disease, private, public, national and international health authorities/agencies have decided to reduce its prevalence and transmission to a point where it should no longer be a public health or a socio-economic concern in affected countries [18, 19]. Since then, the control of the disease has been thusfare based on 2 major large-scale strategies including (1) vector control targeting *Simulium* blackflies and (2) parasite control targeting *O. volvulus* in human. These two strategies have been implemented at large scale in SSA to alleviate the burden of the disease in human populations. The first was implemented in West Africa between 1974 - 2002 as a core activity of Onchocerciasis Control Programme (OCP) [15]. The second strategy targeting parasite control was proposed with the discovery of a safe and effective microfilaricide, ivermectin known under the brand name Mectizan [20]. Since 1987 this drug is freely provided by the pharmaceutical company Merck & Co. [21]. Its availability in sufficient quantities has enabled to continue the control against onchocerciasis in West Africa and its extension in endemic countries of Central and East Africa under the management of African Programme for Onchocerciasis Control (APOC) [22, 23]. The APOC adopted Community-Directed Treatment with Ivermectin (CDTI) as a core strategy with the objective to establish a mechanism for sustained delivery of an annual dose of ivermectin to the entire eligible populations in meso- and hyperendemic communities [24, 25]. Based on the lifespan of adult worms in humans, the expectation of eliminating onchocerciasis after 15 consecutive years was a convincing idea for testing vector control in West Africa and implementing CDTI in all endemic countries in SSA [26]. However, in countries where the impact of vector control and CDTI has been remarkable, the duration of the implementation was above 15 years, corresponding to the maximum life expectancy of adult worms in humans [27].

Evidences of onchocerciasis elimination through CDTI approach in some foci of Latin America and SSA [28, 29, 30, 31, 32, 33, 34, 35] have supported the idea of shifting from the control to elimination of the disease [36]. Besides, the evidences, it is increasingly recognized that onchocerciasis is no longer a public health or socio-economic concern after 15 to 20 successive years of CDTI in some foci in West Africa [37]. In Central Africa, such a statement remains anecdotal after 20 years of CDTI, without any hope or evidence of eliminating the disease.

Epidemiological assessments conducted in Cameroon demonstrated that the transmission of onchocerciasis is still significantly ongoing even in foci where CDTI has been implemented for more than 15 years [35, 38, 39]. Those conducted by APOC in 2012 in 7 CDTI projects in Cameroon showed that elimination of onchocerciasis was feasible in the short term in the Adamaoua II project based on the decline in microfilarial prevalences noted in the region [37]. Since then, the Adamaoua Region, like all the other regions in Cameroon, has been receiving mass treatments of ivermectin every year. This paper reports the situation of onchocerciasis prevalence in the Adamaoua Region after 20 consecutive years of mass treatment of populations with ivermectin.

2. Materials and methods

2.1 Study area

This study was conducted in the Adamaoua Region of Cameroon located between the 6th and 8th degrees of Latitude North and the 10th and 16th degrees of Longitude East. The region covers a surface area of about 62,000 km^2, with altitude ranging between 900 m and 1500 m. The region is situated in the transition zone between the humid equatorial climate of the South and the tropical dry sudano-sahelian climate of the North, influenced by the Adamaoua Plateau [40]. The mean annual rainfall varies between 900 mm to 1,500 mm and is distributed in a continuous rainy season from April to October [41]. A dry season occurs from November to March with a mean annual temperature ranging between 23°C and 26°C [42]. Most of the watercourses in Cameroon have their source in the Adamaoua Region, so called the "water tower of Cameroon". The main rivers of the region include, Mayo Deo, Mbéré and Vina rivers which are of a great importance for human activities such as agriculture and livestock breeding [43, 44]. These rivers exhibit in their watercourse, rapids, cascades and waterfalls favorable to the establishment of *Simulium* blackfly populations.

The health map divides the region in 9 health districts (HDs) including Ngaoundéré Rural, Ngaoundéré Urbain, Bankim, Tibati, Banyo, Ngaoundal, Tignère, Djohong and Meiganga. Onchocerciasis is known to be endemic in the Adamaoua Region for around 10 decades [45]. A total of 29 communities spread across the 9 HDs were selected, with priority given to those visited during the 1998-2002 Rapid Epidemiological Mapping of Onchocerciasis (REMO) conducted in the region.

2.2 Study design and data collection

A cross-sectional survey was conducted between August and September 2020. Data were collected from people living in several communities in the HDs of the Adamaoua Region. In these communities, the collection of biological samples was performed in public places i.e in local health facilities or to a lesser extent in schools for communities without health facilities. In each HD, 2 to 4 communities were prospected in the framework of this study. The selection of the surveyed communities was based on (1) the existence for the community a baseline parasitological data on onchocerciasis from the 1998-2002 REMO studies (2) the high density of human population in certain communities and (3) the location of communities near watercourses with potential *Simulium* breeding sites. The participation in this study by community members was voluntary. However, the inclusion criteria were as follows: (1) be at least 5 years old, (2) reside permanently in the community, (3) for adults, sign an informed consent form during the registration. For children aged 5 to 17 years, a written consent has been signed by a parent or legal guardian. A flow of community members participating in the study was observed in three work stations including (1) a registration station, (2) a skin biopsy collection station, and (3) a medical consultation station (**Figure 1**). At the registration station, all adult participants provided written informed consent. ~~For participants aged below 18 years, a parental or legal guardian consent was required for their enrolment.~~ Personal information of each participant (gender, age, function, permanent residence,) was recorded and a barcode number was assigned to each eligible participant to keep track of the data. At the skin biopsy collection station, 2 superficial skin biopsies were collected from each participant at the left and right posterior iliac crests using a sharp and sterilized 2-mm Holthtype corneoscleral punch. To avoid transmission of potential pathogens, one corneoscleral punch was used per participant for the skin biopsy collection. After use, the corneoscleral punches were subjected to a complete sterilization procedure including 2 stages: (1) immersion for 10 to 15 minutes in bleach solutions of

decreasing concentrations (6, 4 and 2 drops in 100 ml of distilled water) and (2) heating at 120°C for 30 minutes in a portable steam sterilization.

Immediately after collection, each skin biopsy was individually placed into a well of a round-bottom 96-well microtiter plate containing approximately 40µL of physiological saline per well. Plates containing skin biopsies were incubated for 24 hours at room temperature to induce microfilaria emergence [46]. At the end of this period, they were transferred to the local field laboratory for skin biopsy sample examination. In the laboratory, the physiological saline contained in the well was pipetted out and placed on a slide for microscopic examination at 40x magnification for the search of microfilaria and counting when they were identified.

At the medical consultation station, the participant was subjected to onchocerciasis nodule palpation which consists of searching subcutaneous nodules over the body and the skin manifestations of the disease. The participant was then interviewed about his ivermectin treatment history. The answers provided following the interview were recorded on his registration form which finally deposited to the laboratory for archiving result of his skin biopsy examination.

2.3 Data analysis

Personal information, nodule palpation and skin snip biopsy data for each participant were recorded in a specially prepared Excel file. The main indicators of onchocerciasis including microfilaria (mf) and nodule prevalences were expressed as a percentage i.e number of persons positive divided by the number of persons examined × 100. The arithmetic mean of the mf from the two skin biopsies from each participant was calculated and used as a measure of intensity of infection. The Community microfilarial load (CMFL) was calculated as anti-log $\{(\sum \log(x+1))/n\}$ -1, with **x** being the mean of mf/mg of skin and **n** the number of individuals examined [47]. In the framework of this study, the methods described by Coffeng and colleagues [48] were used to convert nodule prevalences of the 1999-2001 survey (typically in samples of adult males aged ≥ 20 years) into microfilarial prevalences (in the population aged ≥ 5 years).

The chi-square test was used to compare: (1) the microfilaria and nodule prevalences reported in this study with those of 1998-2002 (baseline) and 2010-2013 (APOC evaluation), (2) and the reported and surveyed coverage rates recorded in 2019 in the HDs of Adamaoua Region. A two-tailed p-value lower than 0.05 was considered statistically significant.

3.Results

3.1 Demographic data

A total of 4,814 participants from 29 communities selected in the 9 HDs of the Adamaoua region were enrolled in this survey. Men represented 50.4% (N = 2,424) and women 49.6% (N =2,390) with a sex ratio of 1 :1. Participants were aged between 5 and 108 years old (median…). The distribution of participants according to the study population by age group and gender is shown in **Figure 2**. Participants aged between 5 and 30 years represent more than half of the study population (58.45%).

3.2 Status of *O. volvulus* infection in the surveyed communities and health districts

Of the 29 communities surveyed during this study, 11 (≈ 38%) unevenly distributed in 7 HDs were infected by *O. volvulus* microfilariae (**Table 1**). Of these, four were from Meiganga HD, 2 from Ngaoundal HD and 1 from each of the HDs including Bankim, Djohong, Ngaoundéré Rural, Ngaoundéré Urbain and Tibati. No infected community was found in the Banyo and Tignere HDs. The mf prevalence among infected communities ranged from 0.50% [0.02 - 3.23] in Mbakaou (Tibati HD) to 4.34% [1.77-9.63] in Nandeke (Meiganga HD). These mf prevalences were significantly lower (p < 0.05%) compared to that recorded in the 1998-2002 baseline survey where prevalences varied from 36.7% in Alhamissa (Tibati HD) to 87.7% in Djamtari (Tignere HD). Of the 29 communities visited in the 2020 follow-up survey, 22 were previously prospected in the 1998-2002 baseline survey. The variation trend in mf prevalences in these 22 communities from the 1998-2002 baseline survey to the 2020 follow-up (after almost 20 years) is shown in **Figure 3.** Of these 22 communities, 13 (59%) were found free of *O. volvulus* infection and 9 (41%) showed mf prevalences below 5% (**Table 1**) in the 2020 study. In general, the intensity of *O. volvulus* infection characterized by the community microfilarial load (CMFL) was very low (< 0.5 mf/ss) in each of the surveyed communities. The CMFLs assessed in the 2020 survey range from 0.006 mf/ss in Wakwa (Ngaoundere Urbain HD) to 0.129 mf/ss in Nandeke (Meiganga HD). When assessed for participants aged more than 20 years, the CMFLs varied from 0 mf/ss in Mbakaou (Tibati HD) to 0.0716 mf/ss in Zouzami (Meiganga HD) (**Table 1**).

At the level of the HD, the mf and nodule prevalences were estimated by grouping together the communities surveyed in the same HD. **Table 2** summarizes the mf and nodule prevalences recorded in of the HDs during the 2020 survey. Within the HDs of the study

region, nodules prevalences varied from 0.00 [0.00 - 0.77] in Meiganga HD to 2.36 [1.32 - 4.11] in Tibati HD. Overall, the nodule prevalences reported in this 2020 survey were significantly lower ($p < 0.05$) compared to that recorded in the 1998-2002 baseline survey (**Figure 4A**). The mf prevalences varied from 0.00 [0.00 - 0.80] % in Banyo and Tignere HDs to 3.40 [2.16 - 5.23] in Meiganga HD. These prevalences were significantly lower ($p < 0.05$) compared to those recorded in the 1998-2002 and 2010-2013 surveys, with however an exception in Meiganga HD where the mf prevalences of the 2010-2013 and 2020 studies were comparable ($p > 0.05$) (**Figure 4B**). Although not significant, it is important to note that a slight increase in mf prevalence was observed in Meiganga HD during the 2020 study compared to that reported during the 2010-2013 survey. When HDs were grouped together, the nodule and mf prevalences recorded for the whole region were 0.87[0.64 - 1.19] % and 0.77 [0.54 - 1.07] % respectively (**Table 2**).

3.3 Trend in ivermectin treatment

Of the 4,814 participants interviewed for ivermectin treatment, ≈ 28% (N = 1,351) representing almost one third of the studied population said they had never treated with ivermectin, while ≈ 72% (N = 3,463) declared having received ivermectin treatment at least once in the past 6 years (2014-2019). Among those who were treated, ≈ 86% (N = 2,977) received the last treatment in 2019, about 9.7% (N = 336) in 2018 and 4% (N = 141) in 2017, 2016 2015 and 2014. It is worth mentioning that the prevalence of microfilariae in people who did not comply with ivermectin treatment was 0.7%, statistically comparable to that of people who agreed to be treated ($p = 0.8$).

Overall, all communities in the 9 HDs of Adamaoua Region are under ivermectin mass treatment, resulting in 100% geographic coverage for the whole region. The therapeutic coverage rates (TCRs) reported and those surveyed in 2019 in the HDs of Adamaoua Region are shown in **Figure 5**. The TCRs reported by the National Onchocerciasis Control Programme (NOCP) ranged from 78.8% (Tibati HD) to 83.1% (Djohong HD), while those surveyed ranged from 41.3% (Ngaoundere Urbain HD) to 77.5% (Tibati HD). Overall, the TCRs reported by the NOCP were significantly higher than those surveyed, with the exception in Tibati HD, where the reported coverage rate was comparable to that surveyed (**Figure 5**).

In this study, adherence rates of population to ivermectin treatment (i.e the percentage of people who took ivermectin among the eligible population in the community or HD) by gender and age group were also investigated. The population adherence to ivermectin treatment in 2019 by gender in the 9 HDs is shown in **Figure 6**. At the regional level, men and women showed comparable adherence rates during the 2019 ivermectin mass distribution campaign i.e 48.1% (N = 1,433) and 51.9% (N = 1,544) respectively ($p > 0.05\%$).

This equality in adherence to treatment was also noted at HD level, with the exception in the Ngaoundéré Rural HD where adherence of men was significantly higher than that of women (**Figure 6**). The adherence rates of population including men and women were below 65% in Bankim, Banyo, Ngaoundal, Meiganga, Ngaoundére urbain HDs. In Ngaoundere HD, only women adherence was below 65%. In the two other HDs namely Tibati and Tignere the adherence rates of populations were above 65% (**Figure 6**). When HDs were grouped together, the population adherence rate in ivermectin treatment by age group was shown in **Figure 7**. Overall, all age groups showed adherence rates ≤ 65%, with however an exception in the 40-50 age group where the adherence rate was ≈ 70%.

4. Discussion

The objective of this study was to assess the status of onchocerciasis transmission in the Adamaoua Region after two decades of mass treatment with ivermectin. Overall, the nodule and microfilaria prevalences recorded during this 2020 epidemiological study in the surveyed communities and HDs of the region were below 5% with most of them exhibiting zero mf prevalence. Based on the criteria applied for delineating onchocerciasis foci into endemicity levels, all these communities and HDs may be currently classified as sporadic for onchocerciasis [**49**] or hypo-endemic [**50, 51**] for the disease. The CMFLs assessed according to Remme and Colleagues [**52**] showed less than 1 mf/sb indicating low load parasitic loads in human population. The parasitic loads assessed in this study were significantly very low compared to those reported in the same communities during the 1998-2002 REMO data collection conducted in Cameroon [**53, 54**]. This decline in onchocerciasis intensity has previously been reported in Adamaoua II project zone during the 2013 epidemiological assessments conducted in Cameroon as part of the impact evaluation of the CDTI strategy in countries of the African Programme for Onchocerciasis Control [**37**]. The low prevalences of the disease recorded in the surveyed communities is attributed to efforts put in place in controlling the disease through an effective involvement of

communities in ivermectin mass treatment during the past 20 consecutive years. In the context of achieving the elimination of onchocerciasis by 2030, data presented in this study are promising compared to the findings recently reported in other regions of Cameroon [55, 56, 57]. The slightly increase in mf prevalence noted in Meiganga HD during this 2020 study compared to that of the 2013 may be attributed to a decline in the population's adherence to ivermectin treatment. This confirms the low adherence (< 65%) of population in ivermectin treatment noted in most of the surveyed HDs and all age groups of the eligible population. Also, the existence in the region particularly in Meiganga, Ngaoundal and Ngaounderé urbain HDs of systematic non-compliers to ivermectin representing ≈ 28% of the study population and accounting for half of the total infected individuals identified may favour the observed increase in disease prevalence, therefore traducing its recrudescence in some HDs.

Indeed, the non-compliers to ivermectin treatment represent microfilaria reservoirs for the human population, and this may induce active transmission in this environment where microfilaria parasites and blackflies co-exist. Indeed, infectious forms of these microfilaria are spread in the community by *Simulium* blackflies during their blood meals from these non-compliers to individuals free of onchocerciasis. In the savannah zones such as the Adamaoua Region, the spread of these parasitic forms is easier, with the passive movement of blackflies that can reach locations situated tens or even hundreds of kilometres away. Regarding parasite transmission, *Simulium damnosum* s.s, *S. sirbanum* and *S. squamosum* are known as major vectors of onchocerciasis in the Adamaoua Region [58]. Although the presence of these vectors in the region needs to be confirmed through an updated taxonomic study on *Simulium* blackflies, we might highlight that these blackfly species exhibit the phenomenon of "limitation" known as one of the factors favoring the transmission of the disease [59, 60]. The phenomenon is described as the situation where vectors are efficient even at very low parasite densities [61]. The low intensity of the disease expressed by CMFLs (< 1mf/ss) and the limited number of infected individuals reported in this study do not however suggest active transmission of the parasite in this region. Although the decrease in the prevalence of onchocerciasis is noted in the surveyed communities of the region, it should be mentioned that, the local *O. volvulus* parasite population has not yet been reduced to a level below the transmission breakpoint. Data collected during 2010-2013 and 2020 studies from Meiganga reflect this situation and suggest that the transmission of infection in this HD as in others may evolve in a piecemeal fashion, indicating that if no

special attention is given in delivering ivermectin to population, a recrudescence of the disease may occur rapidly in this region.

Although the geographic coverage in ivermectin mass treatment is 100% in the whole region, the surveyed therapeutic coverage rates in 2019 reveal that 5/9 of the HDs are far below the minimum rate of 65% recommended by the former APOC in the framework of onchocerciasis control as a public health problem. Overall, the surveyed coverage rates recorded are lower than those reported by the NOCP in 2019. Although resulting from oral statements of participants, these surveyed coverage rates may be considered as more informative and reflect the status in ivermectin mass treatment in the Adamaoua Region. For the whole region, the surveyed and reported therapeutic coverage rates were comparable only in Tibati HD, suggesting that the implementation of CDTI in this HD has been validated according to WHO recommendations [62]. Indeed, in the other HDs, the therapeutic coverage rates reported by NOCP in 2019 would have been for the most part overestimated at different levels of the recording system used in documenting ivermectin treatment. The reasons for overestimating adherence rates of populations to ivermectin treatment include : (1) the justification of the ability to carry out the task, (2) the renewal of the confidence of the hierarchy and (3) the hope of obtaining a consequent financial or material compensation. The low surveyed therapeutic coverage rates reported in 2019 confirms the poor adherence of the eligible population in ivermectin treatment. This observation is a key information to consider for the elimination of the onchocerciasis in Adamaoua Region. Such a situation calls for additional efforts in delivering ivermectin in the region through increased sensitization of local populations. However, combining the ivermectin treatment with complementary strategies such vector control, enhanced CDTI, community-directed treatment (CDT) with drug combinations or new drugs, and test-and-treat (TNT) [63], may accelerate the elimination of onchocerciasis in this region.

Data reported in this study are close to the threshold of the elimination previously recommended by the former APOC i.e [64] (1) less than 5% prevalence in all surveyed communities and (2) less than 1% in 90% of surveyed communities. All the 29 communities surveyed had mf prevalence below 5% and 70% had mf prevalence below 1%. The current profile of the disease suggests low mf prevalences (< 40%) and CMFLs (< 5mf/ss) across the surveyed communities of the Adamaoua Region. These data are in agreement with WHO guidelines [65], confirming that onchocerciasis is no longer a public health problem in

this region. However, to better characterize the profile of the disease in Adamaoua region, it would be advisable to (1) carry out investigations in several other communities not included in this study, (2) increase the size of the study population and (3) and above all to carry out OV16 tests to demonstrate children's exposure to the infection, as recommended by the WHO. Onchocerciasis is endemic in all regions of Cameroon and its elimination should start in one region and spread to others. The data reported in this study suggest that Adamaoua may be the first region where onchocerciasis elimination should start and be effective in the whole country. Indeed, the Adamaoua Region is known as the water tower of Cameroon, where many rivers especially those infested by blackflies, have their source. Starting the elimination of the disease in this region and extending it to others would be a step towards ridding the country of *O. volvulus* infection. The Adamaoua Region is geographically the transition zone between the southern and northern regions of Cameroon i.e., the southern forest and the northern savannah areas. The fight against *Onchocerca volvulus* infection in Adamaoua-North and Adamaoua-South directions could be an effective operational intervention plan to be adopted for the future elimination of onchocerciasis in Cameroon.

5. Conclusion

The results of this study show a drastic decline in the prevalence of onchocerciasis in the Adamaoua Region after 20 consecutive years of CDTI. The mf prevalences recorded indicate that, the disease has reached relatively low levels of endemicity in the surveyed communities suggesting that the interruption of its transmission may be possible in the near future in this part of the country. However, further additional efforts are expected in the population adherence in ivermectin mass treatment to move towards elimination. This goal may be achieved in a very short term between 2025 and 2030 by improving the annual treatment of populations with ivermectin at 85% therapeutic coverage rates. Furthermore, complementary strategies including more in-depth sensitization of community members targeting non-compliers to ivermectin treatment or physical destruction of *Simulium* breeding sites through the community-directed approach (Slash and Clear) would accelerate the elimination of the disease in this part of the country. These data are of great importance to the Ministry of Public Health, in particularly its NOCP, and could serve as a compass for the elimination of onchocerciasis in Cameroon and Central Africa

6. References

1. Karam M, Schulz-Key H, Remme J. Population dynamics of Onchocerca volvulus after 7 to 8 years of vector control in West Africa. Acta Trop. 1987; 44:445–57.

2. Habbema JDF, Plaisier AP, van Oortmarssen GJ, Remme J. Prospective evaluation of onchocerciasis control strategies. Acta Leiden 1990; 59:387–398

3. Gumisiriza N, Mubiru F, Siewe Fodjo JN, Mbonye Kayitale M, Hotterbeekx A, Idro R, Makumbi I et al. Prevalence and incidence of nodding syndrome and other forms of epilepsy in onchocerciasis-endemic areas in northern Uganda after the implementation of onchocerciasis control measures. Infect. Dis. Poverty. 2020; 9, 12.

4. Murdoch ME, Hay RJ, Mackenzie CD, Williams JF, Ghalib HW, Cousens S et al. A clinical classification and grading system of the cutaneous changes in onchocerciasis. Br J Dermatol. 1993 129: 260–269

5. Buck AA. Onchocerciasis: Symptomatology, Pathology, Diagnosis. Geneva, World Health Organization, 1974

6. Druet-Cabanac M, Bertocchi I, Preux PM. Onchocerciasis and epilepsy in the north west region of Central African Republic. Afr J Neurol Sci. 1998 ; 17 :37–8

7. Boussinesq M, Pion SDS, Ngangue D, Kamgno J. Relationship between onchocerciasis and epilepsy: a matched case-control study in the Mbam Valley, Republic of Cameroon. Trans R Soc Trop Med Hyg. 2002 ;96 :537–41.

8. Pion SDS, Kaiser C, Boutros-Toni F, Cournil A, Taylor MM, Meredith SEO, et al. Epilepsy in onchocerciasis endemic areas : systematic review and meta-analysis of population-based surveys. PLoS Negl Trop Dis. 2009 ; 3 : e461

9. Korevaar DA, Visser BJ. Reviewing the evidence on nodding syndrome, a mysterious tropical disorder. Int J Infect Dis. 2013 ;17(3) : e149–52.

10. Kaiser C, Pion SD, Boussinesq M. Case-control studies on the relationship between onchocerciasis and epilepsy: systematic review and meta-analysis. PLoS Negl Trop Dis. 2013 ;7(3) : e2147.

11. World Health Organization: Onchocerciasis and its Control. Report of a WHO Expert Committee on Onchocerciasis Control. Geneva: World Health Organization; 1995:1–104.

12. Crosskey RW, Howard TM. A revised taxonomic and geographical inventory of world blackfl ies (Diptera: Simuliidae), London, The Natural History Museum; 2004.

13. Noma M, Zoure H, Tekle AH, Enyong P, Nwoke BEB, Remme JHF. The geographic distribution of onchocerciasis in the 20 participating countries of the African Programme for Onchocerciasis Control: (1) priority areas for ivermectin treatment. Parasit Vectors 2014 ; 7 :325.

14. Zoure H, Noma M, Tekle AH, Diggle PJ, Giorgi E, Remme JH. The geographic distribution of onchocerciasis in the participating countries of the African Programme for

Onchocerciasis Control: (2) pre-control endemicity levels and estimated number infected. Parasit Vectors, 2014; 7:326

15. Boatin B, Molyneux DH, Hougard JM, Christensen OW, Alley ES, Yameogo et al. Patterns of epidemiology and control of onchocerciasis in west Africa. J Helminthol. 1997 Jun ;71(2) :91-101. doi: 10.1017/s0022149x00015741. PMID : 9192715.

16. Kamgno J, Bouchite B, Baldet T, Folefack G, Godin C, Boussinesq M. Contribution à l'étude de la répartition des filarioses humaines dans la Province de l'Ouest du Cameroun. Bull Soc Pathol Exot. 1997 ; 90(5) :327–30.

17. Hougard JM, Alley ES, Yameogo L, Dadzie KY & Boatin BA () Eliminating onchocerciasis after 14 years of vector control : a proved strategy. Journal of Infectious Diseases 2001 ; 184, 497–503.

18. Eckholm, E. Conquering an ancient scourge ; river blindness. N.Y. Times Mag. 1989; Jan 8, 20–

19. Bump JB, Benton B, Seketeli A, Liese, BH. West Africa: river blindness- success in scaling up and lessons learned. scaling up poverty reduction: A Global Learning Process and Conference Shanghai, 2004; May 25–27,

20. Cump & Omura. Ivermectin, 'Wonder drug' from Japan: the human use perspective. Proc. Jpn. Acad., 2011; Ser. B 87.

21. Thylefors B. The Mectizan Donation Program (MDP). Ann Trop Med Parasitol. 2008;102 Suppl 1:39-44.

22. Amazigo U. The African Programme for Onchocerciasis Control (APOC). Ann Trop Med Parasitol. 2008;102 Suppl 1:19-22.

23. Crump A, Morel CM, Omura S The Onchocerciasis Chronicle: From the Beginning to the End? *Trends in Parasitology*, 2012 ; 28 : 9 10.1016/j.pt.2011.10.001

24. Molyneux and Davies. Onchocerciasis control : moving towards the millennium. *Parasitol Today.* 1997 ; 13 : 418-425

25. World Health Organization. African Programme for Onchocerciasis Control (WHO/APOC)15 years of APOC, African Programme for Onchocerciasis Control. Ouagadougou, Burkina Faso. 2011 ; 1995-2010.

26. Plaisier AP, van Oortmarssen GJ, Remme J, Habbema JDF. The reproductive lifespan of Onchocerca volvulus in West African savanna. Acta Trop. 1991 ; 48, 271–284

27. Lindblade KA, Arana B, Zea-Flores G, Rizzo N, Porter CH, Dominguez A, et al. Elimination of Onchocerca volvulus transmission in the Santa Rosa Focus of Guatemala. Am J Trop Med Hyg. 2007; 77:334–41.

28. Rodríguez-Pérez MA, Fernández-Santos NA, Orozco-Algarra ME, RodríguezAtanacio JA, Domínguez-Vázquez A, Rodríguez-Morales KB, et al. Elimination of onchocerciasis from Mexico. PLoS Negl Trop Dis. 2015 ; 9(7) : e0003922.

29. World Health Organization/Department of Control of Neglected Tropical Diseases. Guidelines for stopping mass drug administration and verifying elimination of human onchocerciasis. Criteria and procedures. 2016; WHO/HTM/NTD/PCT/2016.1.

30. Diawara L, Traore MO, Badji A, Bissan Y, Doumbia K, Goita SF et al. Feasibility of onchocerciasis elimination with ivermectin treatment in endemic foci in Africa: first evidence from studies in mali and senegal. PLoS Negl Trop Dis. 2009; 3(7): e497. 24.

31. Tekle AH, Elhassan E, Isiyaku S, Amazigo UV, Bush S, Noma M et al. Impact of long-term treatment of onchocerciasis with ivermectin in Kaduna State, Nigeria: first evidence of the potential for elimination in the operational area of the African Programme for Onchocerciasis Control. Parasit Vectors. 2012; 5:28.

32. Traore MO, Sarr MD, Badji A, Bissan Y, Diawara L, Doumbia K et al. Proof-ofprinciple of onchocerciasis elimination with ivermectin treatment in endemic foci in Africa: final results of a study in Mali and Senegal. PLoS Negl Trop Dis. 2012; 6(9): e1825.

33. Higazi TB, Zarroug IM, Mohamed HA, Elmubark WA, Deran TC, Aziz N, et al. Interruption of Onchocerca volvulus transmission in the Abu Hamed focus, Sudan. Am J Trop Med Hyg. 2013 Jul ;89(1):51-7. doi: 10.4269/ajtmh.13-0112. Epub 2013 May 20. PMID : 23690554 ; PMCID : PMC3748488.

34. Katabarwa M, Lakwo T, Habomugisha P, Agunyo S, Byamukama E, Oguttu D,et al. Transmission of Onchocerca volvulus by Simulium neavei in Mount Elgon focus of Eastern Uganda has been interrupted. Am J Trop Med Hyg. 2014 Jun ;90(6) :1159-66. doi: 10.4269/ajtmh.13-0501. Epub 2014 Mar 31. PMID : 24686740 ; PMCID : PMC4047747.

35. Lakwo TL, Garms R, Rubaale T, Katabarwa M, Walsh F, Habomugisha P et al. The disappearance of onchocerciasis from the Itwara focus, Western Uganda after elimination of the vector Simulium neavei and 19 years of annual ivermectin treatments. Acta Trop, 2013; 126: 218–221. doi: 10.1016/j.actatropica .02. 016 PMID: 23458325

36. Mackenzie CD, Homeida MM, Hopkins AD and Lawrence JC. Elimination of onchocerciasis from Africa: possible? Trends in Parasitology Jan 2012, Vol. 28, No. 1 doi :10.1016/j.pt.2011.10.003

37. Tekle AH, Zouré HGM, Noma M, et al. Progress towards onchocerciasis elimination in the participating countries of the African program for onchocerciasis control : epidemiological evaluation results. *Infectious Diseases of Poverty*. 2016 ;5 :66

38. Katabarwa MN, Eyamba A, Nwane P, Enyong P, Yaya S, Baldiagaï J et al. Seventeen years of annual distribution of ivermectin has not interrupted onchocerciasis

transmission in North Region, Cameroon. Am J Trop Med Hyg. 2011 Dec; 85(6) :1041-9. doi: 10.4269/ajtmh.2011.11-0333. PMID : 22144441 ; PMCID : PMC3225149.

39. Kamga GR, Dissak-Delon FN, Nana-Djeunga HC, Biholong BD, Ghogomu SM, Souopgui J et al. Important progress towards elimination of onchocerciasis in the West Region of Cameroon. Parasit Vectors. 2017 Aug 3 ;10(1) :373. doi: 10.1186/s13071-017-2301-7. PMID : 28774318 ; PMCID : PMC5543544.

40. Génieux M. Climatologie Du Cameroun, in : Atlas Du Cameroun. ORSTOM, 1958 ; France.

41. Olivry JC. Fleuves et rivières du Cameroun. Monographie hydrologique. ORSTOM, 1986 ; Paris, p. 733.

42. Suchel JB. Les climats du Cameroun. Ph.D Thesis University of Saint-Etienne, 1998; France.

43. Letouzey R. Observation phytogéographique concernant le plateau africain de l'Adamaoua. Adansonia, sect. B, 1969 ; 29 (3) : 321-337.

44. Letouzey R. Notice de la carte phytogéographique du Cameroun à 1/500 000. Vol. IS-S : Domaine sahélien et soudanien. IRA, Cameroun, Institut de la Carte Internationale de la Végétation, 1985 (Toulouse/France), 26 p.

45. Paris Frédéric. Chronique d'une endémie opportuniste. Développement rural et onchocercose au Nord Cameroun (Foyer du Faro). In : Espace, populations, sociétés, 2000-2. Maladies émergentes et reviviscentes. 1944 ; pp. 241-252 ; doi: https://doi.org/10.3406/espos.2000.1944

46. Collins RC, Brandling-Bennett AD, Hollman RB, Campbell CC, Darsie RF. Parasitological diagnosis of onchocerciasis: comparison of incubation media and incubation times for skin snips. Am J Trop Med Hyg. 1980; 29(1):35–41.

47. Hoerauf A, Mand S, Adjei O, Fleischer B, Büttner DW. Depletion of *Wolbachia* endobacteria in *Onchocerca volvulus* by doxycycline and microfilaridermia after ivermectin treatment. *Lancet.* 2001 ; 357 :1415–1416. doi: 10.1016/S0140-6736(00)04581-5.

48. Coffeng LE, Pion SDS, O'Hanlon S, Cousens S, Abiose AO, Fischer PU et al. Onchocerciasis: The Pre-control Association between Prevalence of Palpable Nodules and Skin Microfilariae. PLoS Negl Trop Dis, 2013 ; 7(4): e2168. doi: 10.1371/journal.pntd.0002168

49. McMahon JE, Sowa SIC, Maude GH, Kirkwood BR. Onchocerciasis in Sierra Leone 2: a comparison of forest and savanna villages. Transactions of the Royal Society of Tropical Medicine and Hygiene, 1988 ; 82 (4) 595-600.

50. Prost A, Hervouet JP, Thylefors B. Epidemiologic status of onchocerciasis. Bull World Health Organ. 1979; 57: 655–62

51. Walker M, Stolk WA, Dixon MA, Bottomley C, Diawara L, Traoré MO et al. Modelling the elimination of river blindness using long-term epidemiological and programmatic data from Mali and Senegal. Epidemics. 2017 Mar ;18 :4-15. doi : 10.1016/j.epidem.2017.02.005. PMID : 28279455 ; PMCID : PMC5340858.

52. Remme J, Ba O, Dadzie KY, Karam M. A force-of-infection model for onchocerciasis and its applications in the epidemiological evaluation of the Onchocerciasis Control Programme in the Volta River basin area. Bull World Health Organ. 1986; 64:667–81.

53. Kamgno J, Boussinesq M. Hyperendémicité de la loase dans la plaine Tikar, région de savane arbustive du Cameroun. *Bulletin de la Société de Pathologie Exotique*, 2001, 94 (4), p. 342-346. ISSN 0037-9085.
Identifiant IRD :

54. Noma M, Nwoke BEB, Nutall I, Tambala PA, Enyong P, Namsenmo A. Rapid epidemiological mapping of onchocerciasis (REMO): its application by the African Programme for Onchocerciasis Control (APOC), Annals of Tropical Medicine & Parasitology, 2002; 96:sup1, S29-S39, DOI: 10.1179/000349802125000637

55. Tsapi EM, Togjom FG, Gamago G-A, Pone JW, Teukeng FFD. Prevalence of onchocerciasis after seven years of continuous community-directed treatment with ivermectin in the Ntui health district Centre region Cameroon. Pan Afr Med J. 2020 ;1 :180.

56. Aza'ah RA, Sumo L, Ntonifor NH, Bopda J, Bamou RH, Nana-Djeunga HC. Point prevalence mapping reveals hotspot for onchocerciasis transmission in the Ndikinimeki Health District, Centre Region, Cameroon. Parasit Vectors. 2020 Oct 16 ;13(1) :519. doi: 10.1186/s13071-020-04387-6. PMID : 33066826 ; PMCID : PMC7565768.

57. Forrer A, Wanji S, Obie ED, Nji TM, Hamill L, Ozano K et al. Why onchocerciasis transmission persists after 15 annual ivermectin mass drug administrations in South-West Cameroon. BMJ Glob Health. 2021 Jan ;6(1) : e003248. doi: 10.1136/bmjgh-2020-003248. PMID : 33431378 ; PMCID : PMC7802695.

58. Traore-Lamizana, Monographie sur la cartographie des gîtes larvaires des vecteurs d'onchocercose au Cameroun, p. 1, Carte montrant la distribution des gîtes larvaires de Simulium sp. Document de l'IRD, 1981.

59. Philippon B. Etude de la transmission d'Onchocerca volvulus (Leuckart, 1893) (Nematoda, Onchocercidae) par Sirnuliurn dainriosuin Theobald, 1903 (Diptera, Simuliidae) en Afrique tropicale. Travaux et Documents de l'O.R.S.T.O.M. No. 63. Paris : Office de la Recherche Scientifique et Technique d'Outre-Mer. ORSTOM, France ans; 1977.

60. Demanou M, Enyong P, Pion SD, Basanez MG, Boussinesq M. Experimental studies on the transmission of Onchocerca volvulus by its vector in the sanaga valley

(Cameroon): simulium squamosum B. Intake of microfilariae and their migration to the haemocoel of the vector. Ann Trop Med Parasitol. 2003;97(4):381–402.

61. Amuzu H, Wilson MD, Boakye DA. Studies of Anopheles gambiae sl (Diptera: Culicidae) exhibiting different vectorial capacities in lymphatic filariasis transmission in the Gomoa district, Ghana. Parasit Vectors. 2010 ;3 :85.

62. WHO. 2016. Coverage evaluation surveys for preventive chemotherapy: *field guide for implementation.* World Health Organization, November 2016.

63. Boussinesq M, Fobi G and Kuesel AC, 2017. Alternative treatment strategies to accelerate the elimination of onchocerciasis. Int Health 2018; 10 : i40–i48 doi:10.1093/inthealth/ihx054

64. African Program for Onchocerciasis Control/World Health Organization. Conceptual and operational framework of onchocerciasis elimination with ivermectin treatment. World Health Organization/African Program for Onchocerciasis Control, 2010. WHO/APOC/MG/10.1, JAF 16.6 II.

65. World Health Organization. Report of the external mid-term evaluation of the African Programme for Onchocerciasis Control; JAF 16.8. 2010. http://who.int/ apoc/MidtermEvaluation_29Oct2010_final_printed.pdf. Accessed 21 Mar 2018.

Declaration

Ethics approval and consent to participate

The study protocol was approved by the Cameroon National Ethics Committee (N◦2020/08/1286/CE/CNERSH/SP), on 21 August 2020. Each study participant aged 18 and above personally affixed both thumbprints to the informed consent form presented to them to confirm their participation in the study. Parental consent was obtained for participants aged 5 to 17 years

COMPETING INTERESTS

The authors declare that they have no competing interests.

FUNDING

This study was funded by kwF, through the project for Control of Neglected Tropical Diseases in Central Africa (MTN/OCEAC) Internal BMZ N° 2015. 69.227+ 2016. 68797.

AUTHORS' CONTRIBUTION

PN, HND and JK designed the study protocol. PN, NZT, AD, GRN, MT, JB, SB, AB, AN, HN, PM, GM collected data on the field. PN, HND and YEN supervised and monitored data collection and management. PN, HND and JK reviewed the results and contributed to their interpretation. PN, FNB and YEN conducted data analysis, PN a prepared the first draft of

the manuscript. All authors participated in the interpretation of results and revisions of the manuscript. All authors read and approved the final manuscript.

AVAILABILITY OF DATA AND MATERIALS

The data we have presented in this paper may be free of access to the general public and should not be restricted. They should be accessible on the Internet without any particular requirements, as they will help to inform the scientific community about the situation of onchocerciasis in the Adamaoua region. All data are in the manuscript and/or supporting information files.

ACKNOWLEDGMENTS

The authors are grateful to the Regional Health Delegate of Adamaoua Region, all health staff at the levels of the health districts and health areas, the traditional chiefs and their respective populations for their active involvement for data collection.

AUTHORS' INFORMATION

PN, Email: philino07@yahoo.fr, Medical Entomologist, Research Scientist at the High Institute of Scientific and Medical Research (ISM) Yaoundé Cameroon.

HND, Email: nanadjeunga@crfilmt.org, Molecular Parasitologist, at the High Institute of Scientific and Medical Research (ISM) Yaoundé Cameroon

AD, Email: domche_andre@yahoo.fr, Medical Entomologist, Ph.D student at the University of Yaoundé I/Research assistant at the High Institute of Scientific and Medical Research (ISM) Yaoundé Cameroon.

GRN, Email: njitchouang@yahoo.fr, Biochemist, at the High Institute of Scientific and Medical Research (ISM) Yaoundé Cameroon.

JK, Email: kamgno@crfilmt.org, Epidemiologist, Director of the High Institute of Scientific and Medical Research (ISM) Yaoundé Cameroon

YEN: Email: emalio2002@yahoo.fr, Statistician at the High Institute of Scientific and Medical Research (ISM) Yaoundé Cameroon

NZT: Email: narcissenzunetoche@yahoo.fr, Biochemist, Ph.D student at the University of Yaoundé I/Research assistant at the High Institute of Scientific and Medical Research (ISM) Yaoundé Cameroon.

MT: Email: augustaflore@yahoo.fr, Ph.D student at the University of Buea I/Research assistant at the High Institute of Scientific and Medical Research (ISM) Yaoundé Cameroon.

JB: f_bopda@yahoo.fr, laboratory technician at the High Institute of Scientific and Medical Research (ISM) Yaoundé Cameroon.

FNB: Email: fesuhbe@yahoo.co.uk,

SB: Email: mbickmensteve@yahoo.fr; laboratory technician at the High Institute of Scientific and Medical Research (ISM) Yaoundé Cameroon

AB: Email: aubinsantos1988@yahoo.fr; laboratory technician at the High Institute of Scientific and Medical Research (ISM) Yaoundé Cameroon

AN: Email: nkouelle@yahoo.fr; Project Manager at International Eye Foundation

HO: Email: obamahonore@yahoo.fr, Data manager at the National Onchocerciasis Control Program/ Ministry of Public Health

PM: Email: mepafel2001@yahoo.fr, Data manager at the National Onchocerciasis Control Program/ Ministry of Public Health

GNA: Email gb_nko@yahoo.fr, Head of Department of Neglected Tropical Diseases/ Ministry of Public Health

BDB: biholong_di@yahoo.fr; Permanent secretary of National Onchocercisasis Control Program

Table 1 : Mf prevalences in communities prospected during the 1998-2002 and 2020 epidemiological surveys conducted in Adamaoua Region.

**Health District**	**Community**	**Geographic coordinates**		**Baseline survey (1998-2002)**		**Follow up survey (2020)**			
		**Latitude**	**Longitude**	**# of persons examined**	**Mf prevalence (1998-2002)**	**# of persons examined**	**Mf preva-lence [95%IC] (2020)**	**CMFL (All)**	**CMFL (+20yrs)**
Bankim	Mgbandji*	5°59'22.5''	11°16'36.5''	30	80.2	87	1.14 [0.06 - 7.13]	0.023	0.035
	Koumtchoum*	6°26'81.8''	11°12'14.8''	30	38.9	227	0	-	-
	Moinkoing*	6°02'58.3''	11°24'40.2''	32	55.7	100	0	-	-
Banyo	Mayo Dinga*	6°25'18.2''	11°34'15.0''	30	60.3	173	0	-	-
	Mbamti*	6°41'02.7''	12°04'00.0''	32	59.3	180	0	-	-
	Yimbere*	6°27'35.3''	11°34'269''	30	50.9	239	0	-	-
Djohong	Gbatoua*	6°50'42.6"	14°42'56.4"	33	65.7	146	0.68 [0.03 - 4.32]	0.008	0.013
	Yamba*	7°06'21.0"	15°12'20.8"	30	71.0	129	0	-	-
Meiganga	Korekoni*	6°08'09.3"	14°33'48.0"	33	55.4	225	4.00 [1.96 - 7.70]	0.127	0.051
	Lokoti	6°22'00.0"	14°20'00.0"	43	-	89	1.12 [0.05 - 6.97]	0.022	0.022
	Zouzami*	6°37'08.3"	14°32'20.0"	30	80.3	168	2.97 [1.10 - 7.17]	0.049	0.071
	Nandeke*	6°27'09.7"	14°13'47.1"	38	56.3	138	4.34 [1.77 - 9.63]	0.129	0.047
Ngaoundal	Boy-Baya	6°25'26.4"	13°23'10.7"	31	-	56	3.57 [0.62 - 13.38]	0.048	0.048
	Bagodo*	6°25'26.4"	13°23'10.7"	50	55.6	110	0	-	-
	Ngaoundal*	6°28'06.0"	13°16'09.4"	31	46.6	206	2.91 [1.18 - 6.52]	0.050	0.050
Ngaoundere	Nganha*	7°26'08.8"	13°55'43.2"	30	80.2	169	0.59 [0.03 - 3.75]	0.010	0.010
Rural	Bakari Bata*	6°55'41.9''	14°35'51.7"	33	59.1	213	0	-	-
	Berem*	7°33'03.7''	13°55'28.4''	30	86.7	299	0	-	-
	Nyassar*	7°31'40.1''	14°01''43.7''	30	82.5	281	0	-	-
Ngaoundere	Wakwa*	7°16'05.8''	13°33'01.6''	30	66.0	95	4.21 [1.35 - 11.03]	0.026	0.051
Urbain	Gadamabanga	7°21'37.7"	13°35'05.5"	32	-	78	0	-	-
	Mballang	7°18'00.8''	13°44'30.2''	30	-	110	0	-	-
Tibati	Mbakaou*	6°18'13.9''	12°47'48,0''	52	67.4	197	0.50 [0.02 - 3.23]	0.006	0
	Alhamissa*	6°30'01,9''	12°41'58.9''	30	36.7	113	0	-	-
	Djombi	6°41'28.8''	12°36'18.4''	33	-	240	0	-	-
Tignere	Libong	7°23'21.5''	13°00'06.4''	30	-	171	0	-	-
	Mayo-Djarandi	7°22'05.0''	12°38'59.3''	30	-	175	0	-	-
	Mayo-Kaloua*	7°05'13.7''	12°28'15.0''	23	74.1	187	0	-	-
	Djamtari*	7°42'03.4''	12°15'01.9''	36	87.7	213	0	-	-

*Commmunty prospected in 1998-2002 and 2020 ; **#** : number ; **Mf**: microfilaria; **-**: No data available

Table 2 : Microfilaria and nodule prevalences recorded in the health districts of the Adamaoua Region

**Health District**	N	**Prevalence**	
		**Nodule**	**Mf**
Bankim	414	0.96 [0.31- 2.63]	0.24 [0.01 - 1.55]
Banyo	592	1.18 [0.52 - 2.53]	0.00 [0.00 - 0.80]
Djohong	275	2.18 [0.89 - 4.92]	0.36 [0.02 - 2.33]
Meiganga	620	0.00 [0.00 - 0.77]	3.40 [2.16 - 5.23]
Ngaoundal	372	0.53 [0.09 - 2.14]	2.15 [1.00 - 4.36]
Ngaoundere rural	962	0.10 [0.00 - 0.67]	0.10 [0.00 - 0.67]
Ngaoundere urbain	283	0.71 [0.12 - 2.81]	1.41 [0.45 - 3.82]
Tibati	550	2.36 [1.32 - 4.11]	0.18 [0.01 - 1.17]
Tignere	764	0.94 [0.41 - 2.01]	0.00 [0.00 - 0.64]
**TOTAL**	**4,814**	**0.87 [0.64 - 1.19]**	**0.77 [0.54 - 1.07**]

N : number of persons examined ; **Mf**: microfilaria

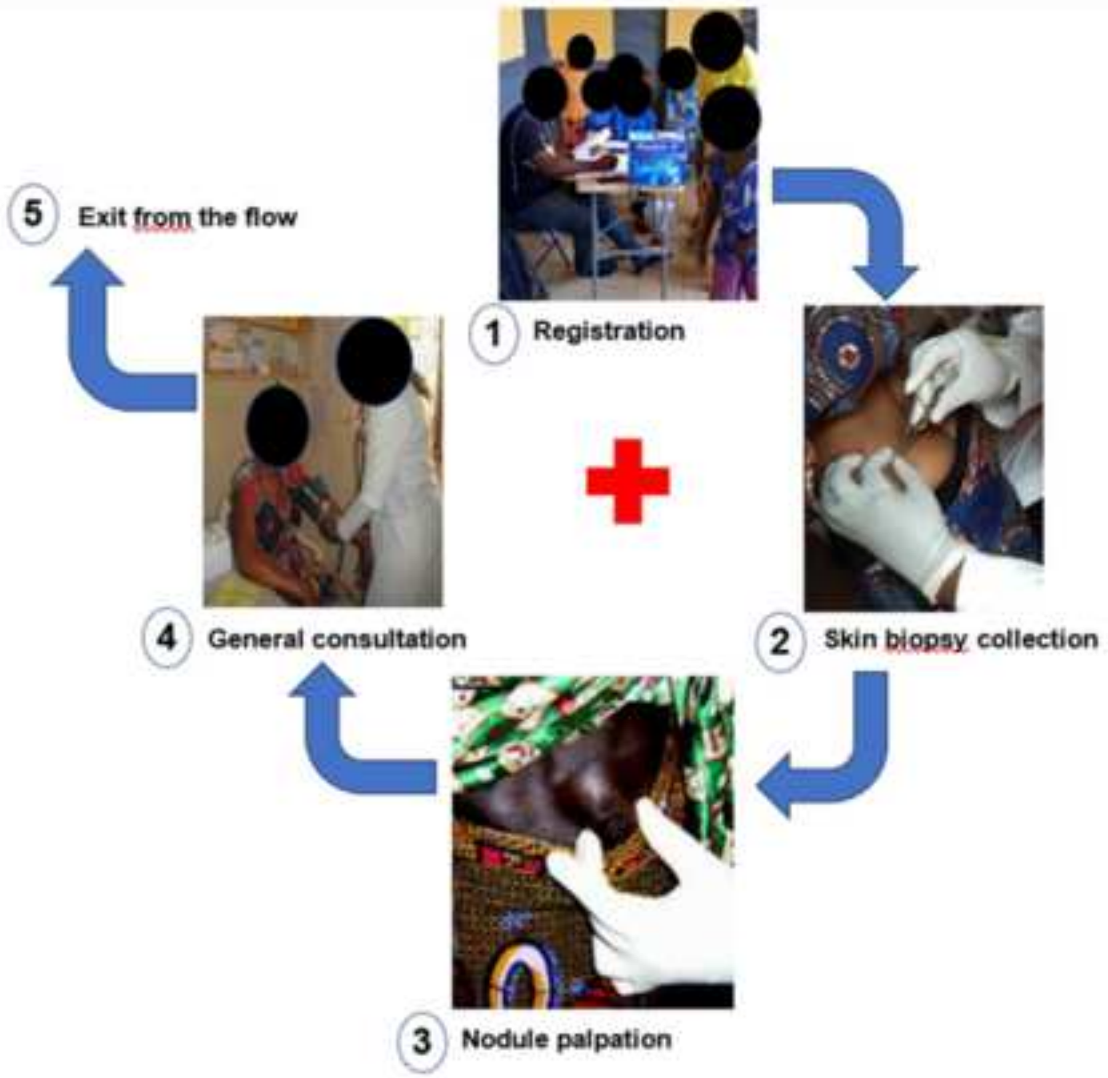

Figure 1. Participant progress flow during data collection procedure

Figure

Click here to access/download;Figure;Figure-2 (1).tiff

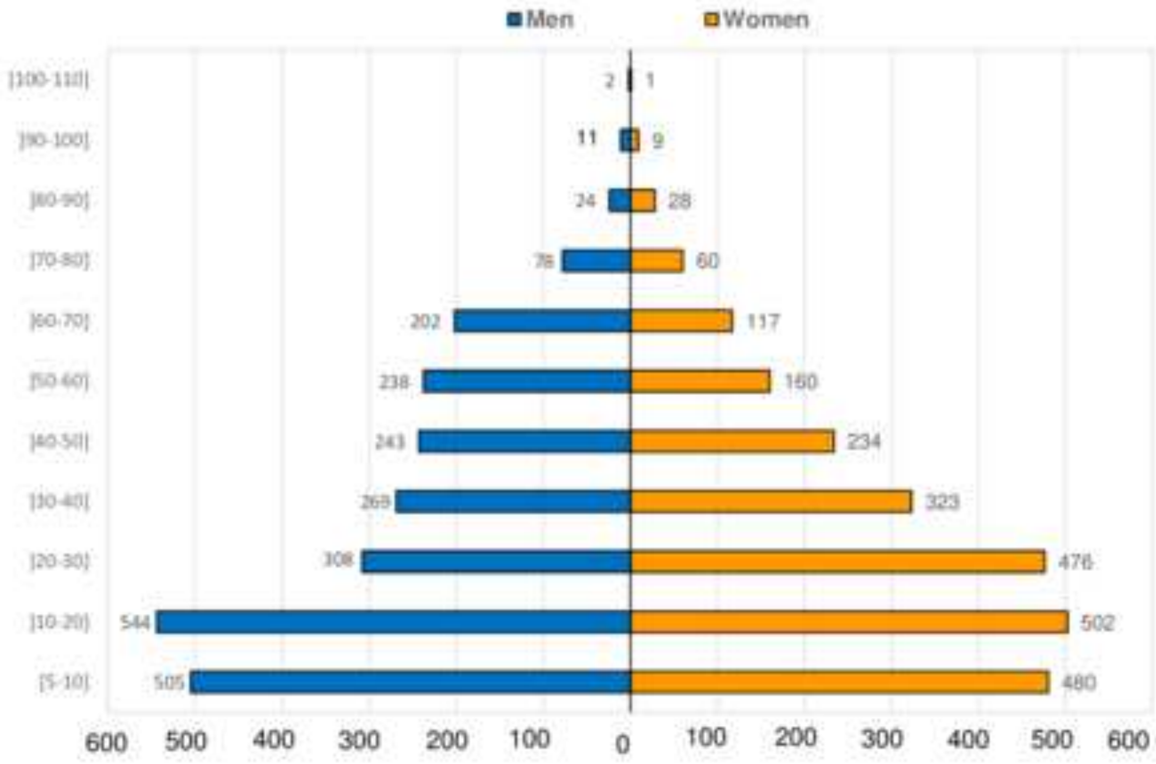

Figure 2 : Distribution of the study population according to gender and age groups

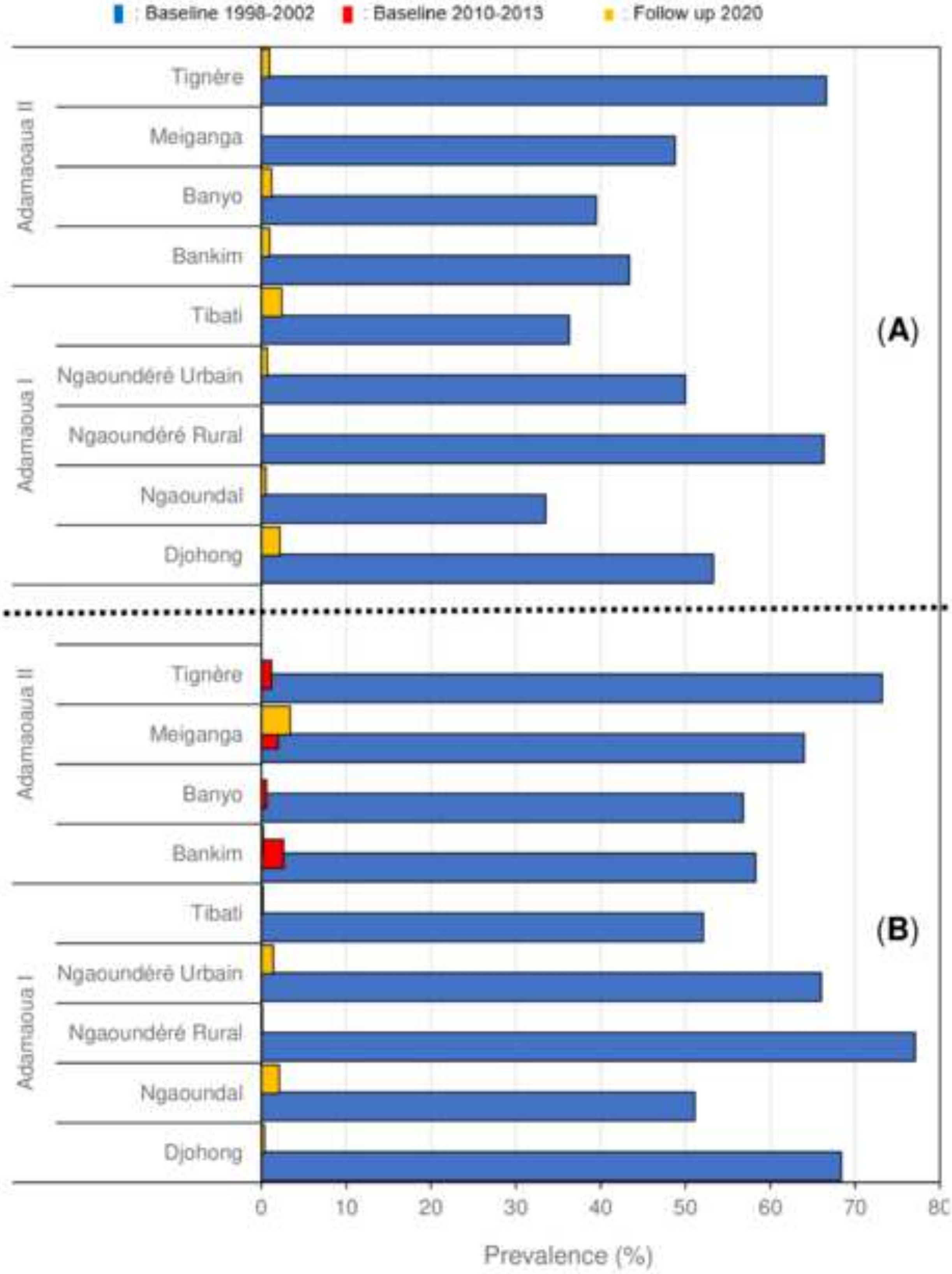

Figure 3 : Nodule (**A**) and microfilaria (**B**) prevalences recorded in surveyed Health Districts

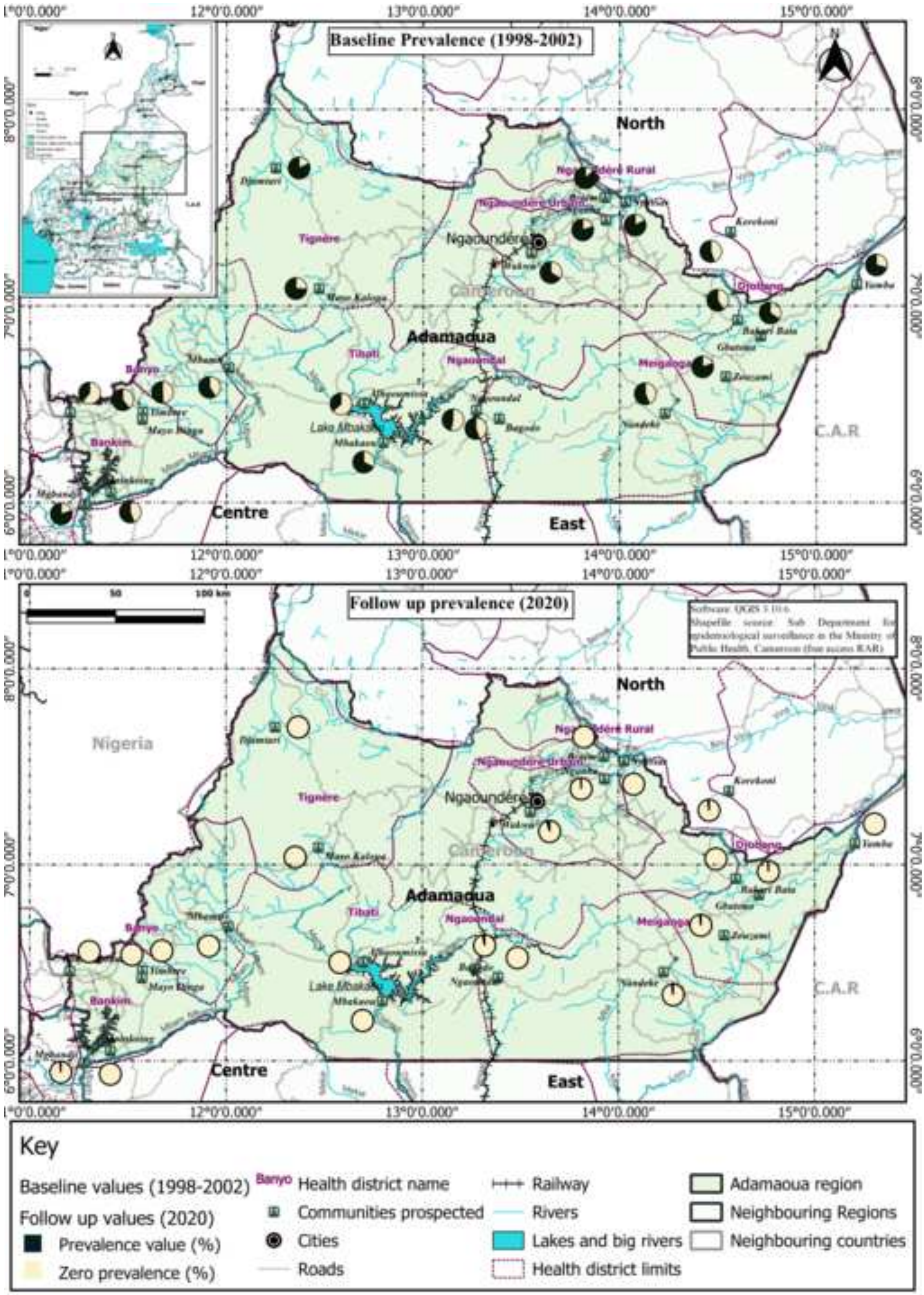
Baseline Prevalence (1998-2002)
Follow up prevalence (2020)
North
Adamaoua
Centre
East
C.A.R
Nigeria
Ngaoundere
Tignère
Tibati
Banyo
Bankim
Meiganga
Djohong
Ngaoundal
Lake Mbakaou
12°0'0.000"
13°0'0.000"
14°0'0.000"
15°0'0.000"
8°0'0.000"
7°0'0.000"
6°0'0.000"
0
50
100 km
Key
Baseline values (1998-2002)
Follow up values (2020)
Prevalence value (%)
Zero prevalence (%)
Banyo
Health district name
Communities prospected
Cities
Roads
Railway
Rivers
Lakes and big rivers
Health district limits
Adamaoua region
Neighbouring Regions
Neighbouring countries

 Click here to access/download;Figure;Figure-5 (1).tiff

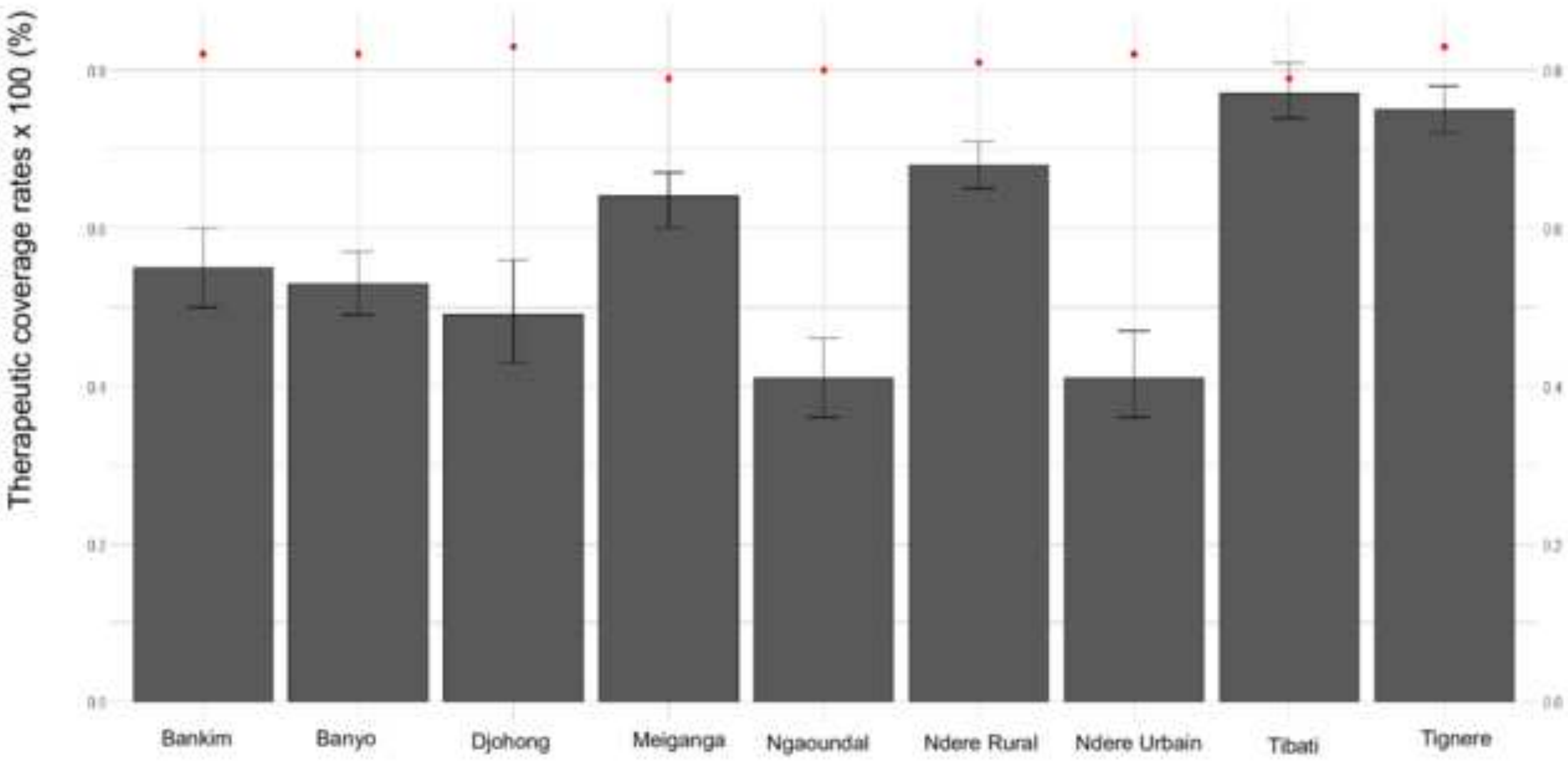

Figure 5 : Therapeutic coverage rates reported (**red dot**) and surveyed (**histrogram bar**) in the health districts of Adamaoua Region in 2019.

Ndere : Ngaoundere

Figure Click here to access/download;Figure;Figure-6 (1).tiff

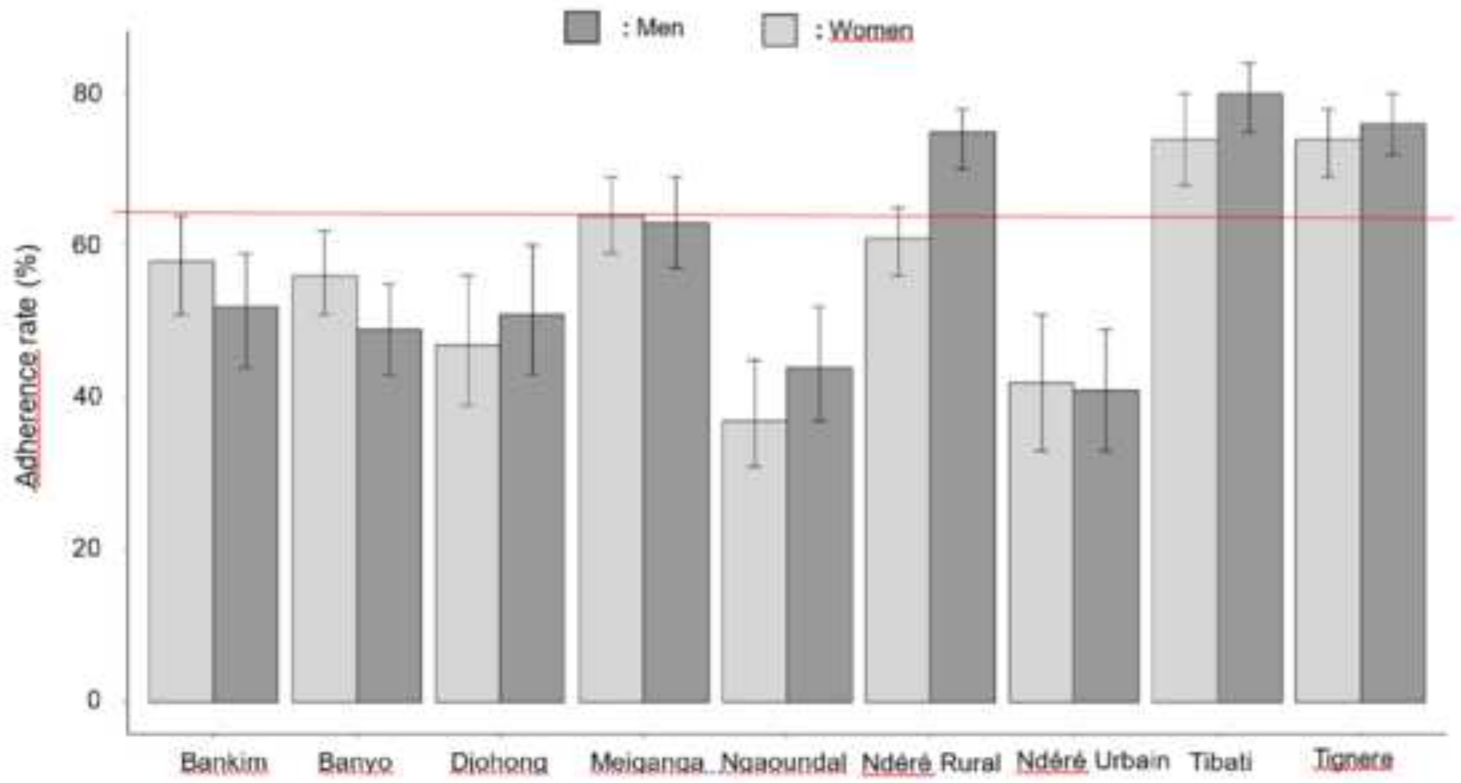

Figure 6 : Population adherence rates by gender to ivermectin treatment during the 2019 mass distribution in the 9 health districts of Adamaoua Region
Ndéré : Ngaoundéré

Click here to access/download;Figure;Figure-7(1).tiff

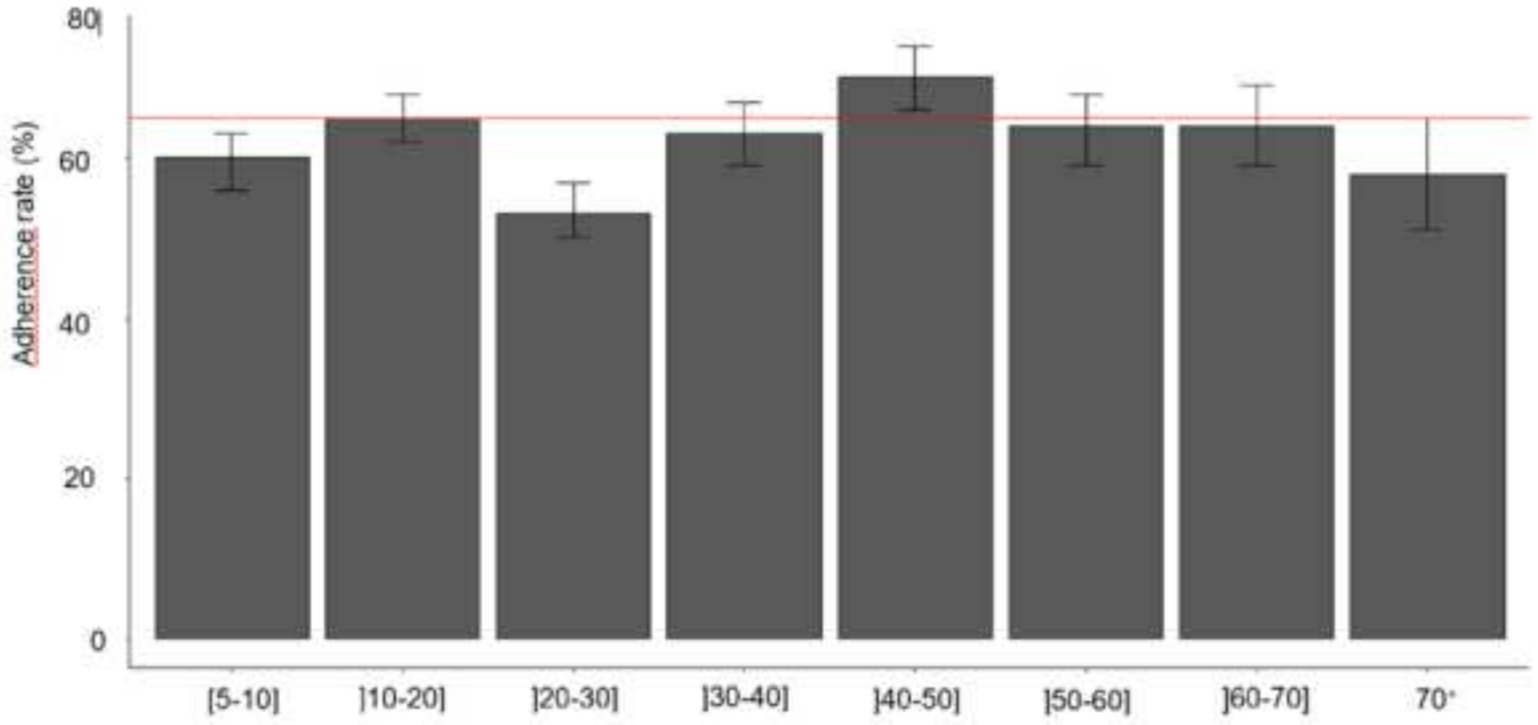

Figure 7 : Population adherence rate according to age groups during the 2018 Ivermectin mass treatment in the 9 health districts of the Adamaoua Region

Answers to questions of Reviewer N°1

Reviewer #1:
The objectives are clearly articular; the study design is appropriate. However, sampling and methodology and limitations at different point are not clearly presented limitations. Also, statistically analysis to make the conclusion appear inadequate

Answer

We have provided details on the collection of biological samples used in this study and have indicated the criteria used to select certain communities for data collection during this study. We have also added to the text the inclusion criteria for participants enrolled in this study

Reviewer #1: Results need to been well articular towards objectives. As mentioned earlier, statistical analysis could be done better

Answer

Our objective was to assess the impact of mass treatments on the evolution of onchocerciasis in the Adamaoua Region. To do this, we assessed the prevalence of nodules and microfilariae at the community and health districts levels. Then we examined the population's adherence to ivermectin treatment accoding to gender and age groups.

We made statistical analyses of data more consistent regarding the adherence of population to ivermectin teatment

Reviewer #1: Conclusion appear a bit premature without indebt consideration of sampling, analysis and diseases epidemiology. Limitation of sampling, analysis and disease epidemiology need to be considered in analysis, discussion and conclusion

Answer

Yes, we agree that this conclusion is premature since we have not surveyed all the communities and all the eligible individuals in the framewoork of this study. We have used careful language in the text so as not to confirm that we are in a position to eliminate onchocerciasis in this region.

Reviewer #1: minor revision

Answer

We have made a number of changes to the spelling of certain words, the comprehension of certain part of the text and revised the titles of tables and figures. We would like to thank you for all these observations

We would like to thank you for your pertinent observations and suggestions to improve the scientific quality of this paper. We remain available to follow your recommendations that may allow the publication of this paper in your journal. Our sincere thanks

Answers to questions of Reviewer N°2

Reviewer #2: The objectives of the study are clearly articulated, with a clear testable hypothesis
- the study design is not appropriate to address the stated objectives.e.g the study is impact of control strategy on onchercerciasis transmission and not on elimination as the title suggests.
- the population is clearly described and very appropriate for the hypothesis being tested
- the sample size is quite adequate to address the hypothesis being tested
- correct statistical methods were adopted for data analysis and support for conclusion
- ethical issues concerning the use of corneo- scleral punch in oncherciasis diagnosis was not addressed.

Answer

We have modified the title of this paper in line with your comments and suggestions. We have discussed the ethical considerations associated with the transmission of pathogens by corneo-scleral punches used during the collection of skin biopsies.

Reviewer #2: The analysis presented were in tandem with analysis plan. The authors did not present the pre.control data of the study communities to enable for proper analysis of the control strategy through CDTI this is another drawback.
The results were clearly but not completely presented
- the figures, tables and images are not sufficient to represent the quantity collected in the study.

Answer

Thank you for this observation. We presented in Table 1 the baseline data on mf prevalences that were obtained from the transformation of nodule prevalences according to the formula of Coffeng et al., 2013. We then compared these baseline data with those obtained in this study to monitor the impact of ivermectin mass treatments on the disease transmission. We have added other indications (year of data collection) to columns 6 and 8 of Table 1 to make it easier for understanding.

Reviewer #2: The conclusion are fairly supported by the data presented
- the limitations of the study are stated in the manuscript.e.g not using ov16 Elisa to test children in the quest for elimination
- the authors stated how the results of this can be utilized for policy formulation in the control and elimination of oncherciasis
- the public health relevance of the study is adequately stated

Answer

Thank you very much for this comment. It has enabled us to express caution in our conclusion and to discuss the results further by mentioning their limitations and envisage the elimination of onchocerciasis in this part of the country.

Reviewer #2: The topic should be changed to read ' status of human oncherciasis transmission in the Adamaoua region of Cameroon after 20 years of ivermectin control ' this is due to the fact that the study is assessing impact of ivermectin control and not assessing elimination

Answer

Thank you very much, we've changed the title of the paper to reflect your comments.

We would like to thank you for your pertinent observations and suggestions to improve the scientific quality of this paper. We remain available to follow your recommendations that may allow the publication of this paper in your journal. Our sincere thanks

Click here to access/download
Revised Article with Changes Highlighted
Manuscript_2024_Major changes highlighted_Rev.docx
