# Supplementary material for: Status of human onchocerciasis transmission in the Adamaoua region of Cameroon after 20 years of ivermectin mass distribution
Source: PLoS Negl Trop Dis. 2025 Mar 4;19(3):e0011511. doi: 10.1371/journal.pntd.0011511 (PMC11925462; doi:10.1371/journal.pntd.0011511)
Supplement: Attachment — Submitted filename: PNTD-D-23-00854_R2_reviewer_3.pdf [file pntd.0011511.s011.pdf]

PLOS Neglected Tropical Diseases

Status of human onchocerciasis transmission in the Adamaoua region of Cameroon after 20 years of ivermectin mass distribution

--Manuscript Draft--

**Manuscript Number:**	PNTD-D-23-00854R2
**Full Title:**	Status of human onchocerciasis transmission in the Adamaoua region of Cameroon after 20 years of ivermectin mass distribution
**Short Title:**	Current status of onchocerciasis in Adamaoua Region of Cameroon
**Article Type:**	Research Article
**Keywords:**	Onchocerciasis - Ivermectin - CDTI - Elimination - Adamaoua Region - Cameroon.
**Abstract:**	Introduction<br>Although evidences of onchocerciasis elimination through community directed treatment with ivermectin (CDTI) are reported in some foci in West and East Africa, in Central Africa the situation does not sound promising, despite two decades treatment in some foci of the sub-region. This paper describes the current situation of onchocerciasis in Adamaoua Region of Cameroon after 20 consecutive years of ivermectin mass treatment.<br>Material and methods The study was conducted between August and September 2020 in Adamaoua Region of Cameroon. Onchocerciasis endemicity was assessed through parasitological and clinical diagnosis. Microfilarodermia and nodule prevalences assessed in 2020 were compared to those of 1998-2002 and 2010-2013 surveys using ANOVA and Welch's unequal variances t-test.<br>Results<br>A total of 4,814 participants aged between 5 and 108 years, including 50.4% men and 49.6% women with a sex ratio of 1:1. For the whole region the nodule and microfilaria prevalence were 0.87 [0.64 - 1.19] % and 0.77 [0.54 - 1.07] % respectively. At the community level, the mf prevalences ranged from 0.5% to 4.5%. Globally, the community microfilarial loads (CMFL) were < 0.5 mf/ss. The surveyed therapeutic coverage rates were between 40% and 78% but lower than those reported (79% - 83%). The adherence rates in ivermectin treatment of all age groups of the population were below 65%, except for the 40-50 age group where it was ≈70%.<br>Conclusion The results of this study show a drastic decline in onchocerciasis prevalences after 20 consecutive years of CDTI, indicating a significant progress towards the elimination of the disease in Adamaoua Region. However, additional efforts are needed to increase the population adherence in ivermectin treatment to achieve the elimination of the disease in this Region. These data are useful for the Ministry of health in guiding the elimination procedure of onchocerciasis in Cameroon.
**Additional Information:**	
**Question**	**Response**
**Government Employee**<br><br>Are you or any of the contributing authors an employee of the United States government?<br><br>Manuscripts authored by one or more US Government employees are not copyrighted, but are licensed under a CC0 Public Domain Dedication, which allows	No - No authors are employees of the U.S. government.

Powered by Editorial Manager® and ProduXion Manager® from Aries Systems Corporation

unlimited distribution and reuse of the article for any lawful purpose. This is a legal requirement for US Government employees.<br><br>This will be typeset if the manuscript is accepted for publication.	
**Financial Disclosure**<br><br>Enter a financial disclosure statement that describes the sources of funding for the work included in this submission. Review the submission guidelines for detailed requirements. View published research articles from *PLOS Neglected Tropical Diseases* for specific examples.<br><br>This statement is required for submission and **will appear in the published article** if the submission is accepted. Please make sure it is accurate.<br><br>**Funded studies**<br>Enter a statement with the following details:<br>• Initials of the authors who received each award<br>• Grant numbers awarded to each author<br>• The full name of each funder<br>• URL of each funder website<br>• Did the sponsors or funders play any role in the study design, data collection and analysis, decision to publish, or preparation of the manuscript?<br><br>Did you receive funding for this work?	Yes
**Competing Interests**<br><br>On behalf of all authors, disclose any competing interests that could be perceived to bias this work.<br><br>This statement will be typeset if the manuscript is accepted for publication.	No competing interests

Review the instructions link below and PLOS NTDs' competing interests policy to determine what information must be disclosed at submission.	
**Data Availability**<br><br>Provide a **Data Availability Statement** in the box below. This statement should detail where the data used in this submission can be accessed. This statement will be typeset if the manuscript is accepted for publication.<br><br>Before publication, authors are required to make all data underlying their findings fully available, without restriction. Review our PLOS Data Policy page for detailed information on this policy. Instructions for writing your Data Availability statement can be accessed via the Instructions link below.	The data we have presented in this paper may be free of access to the general public and should not be restricted. They should be accessible on the Internet without any particular requirements, as they will help to inform the scientific community about the situation of onchocerciasis in the Adamaoua region. All data are in the manuscript and/or supporting information files
Please add funding details.<br>as follow-up to "**Financial Disclosure**<br><br>Enter a financial disclosure statement that describes the sources of funding for the work included in this submission. Review the submission guidelines for detailed requirements. View published research articles from *PLOS Neglected Tropical Diseases* for specific examples.<br><br>This statement is required for submission and **will appear in the published article** if the submission is accepted. Please make sure it is accurate.<br><br>**Funded studies**<br>Enter a statement with the following details:<br>• Initials of the authors who received each award<br>• Grant numbers awarded to each author<br>• The full name of each funder<br>• URL of each funder website<br>• Did the sponsors or funders play any role in the study design, data collection and analysis, decision to publish, or preparation of the manuscript?	This study was funded by kwF, through the Project for Control of Neglected Tropical Diseases in Central Africa (MTN/OCEAC) Internal BMZ N° 2015. 69.227+ 2016. 68797. This funding has been allocated to the NGDO International Eye Foundation, which supports the Ministry of Public Health in the fight against onchocerciasis in the Adamaoua Region. The funders had no role in study design, data collection, data analysis, data interpretation, writing of the report, or the decision to submit for publication

Did you receive funding for this work?"	
Please select the country of your main research funder (please select carefully as in some cases this is used in fee calculation).<br>as follow-up to "**Financial Disclosure**<br><br>Enter a financial disclosure statement that describes the sources of funding for the work included in this submission. Review the submission guidelines for detailed requirements. View published research articles from *PLOS Neglected Tropical Diseases* for specific examples.<br><br>This statement is required for submission and **will appear in the published article** if the submission is accepted. Please make sure it is accurate.<br><br>**Funded studies**<br>Enter a statement with the following details:<br>• Initials of the authors who received each award<br>• Grant numbers awarded to each author<br>• The full name of each funder<br>• URL of each funder website<br>• Did the sponsors or funders play any role in the study design, data collection and analysis, decision to publish, or preparation of the manuscript?<br><br>Did you receive funding for this work?"	GERMANY - DE

Manuscript
Click here to access/download;Manuscript;Manuscript_07_2024docx.docx

Status of human onchocerciasis transmission in the Adamaoua region of Cameroon after 20 years of ivermectin mass distribution

Philippe Nwane[*1,2], Hugues Clotaire Nana-Djeunga[1], Narcisse Nzune Toche[1], André Domché[1,2], Fesuh Nono Bertrand[3], Yannick Emalio Niamsi[1], Guy Roger Njitchuang[1], Martine Augusta Flore Tsasse[1], Jean Bopda[1], Steve Bickmen[1], Aubin Balog[1], Alexis Nkwelle[4], Patrice Nkwelle[4†], Clarisse Ebene[5], Honoré Obama[5], Paul Messi[5], Benjamin Biholong[5], Serge Billong[5], Georges Nko'o Ayissi[6] and Joseph Kamgno[1,7]

Author details

[1]Higher institute of scientific and medical research (ISM), P.O. Box 5797 Yaoundé, Cameroon ;
[2]Laboratory of General Biology, University of Yaounde I, P.O. Box 337, Yaoundé, Cameroon
[3]National Advanced School of Engineering, University of Yaoundé I, Yaoundé, Cameroon
[4]International eye Foundation, Cameroon ;
[5]National Onchocerciasis Control Program/Ministry of Public Health, Yaounde, Cameroon ;
[6]Departement of Neglected Tropical Diseases Department/Ministry of Public Health, Yaounde, Cameroon ;
[7]Faculty of Medicine and Biomedical Sciences, University of Yaounde I, Yaounde, Cameroon.

† Deceased some months after the data analysis.

ABSTRACT

Introduction

Significant progress has been made in onchocerciasis control through mass distribution of ivermectin among affected human populations, fostering optimism for disease elimination. However, despite these considerable advances, the elimination of the disease remains a major challenge in many African foci. This paper describes the current situation of onchocerciasis in Adamaoua Region of Cameroon after 20 consecutive years of ivermectin mass treatment.

Material and methods

The study was conducted between August and September 2020 in Adamaoua Region of Cameroon. Onchocerciasis endemicity was assessed through parasitological and clinical diagnosis. Microfilarodermia and nodule prevalences assessed in 2020 were compared to those of 1998-2002 and 2010-2013 surveys using the chi-square (χ^2) statistic test.

Results

A total of 4,814 participants aged between 5 and 108 years, including 50.4% men and 49.6% women. For the examined sub-sample representing the whole Adamaoua region, the nodule and microfilaria prevalence were 0.87 [0.64 - 1.19] % and 0.77 [0.54 - 1.07] %, respectively. At the community level, the mf prevalences ranged from 0.5% to 4.5%. Globally, the community microfilarial loads (CMFL) were < 0.5 mf/ss. The survey therapeutic coverage rates were between 40% and 78%, lower than those reported (79% - 83%). The coverage rates in ivermectin treatment of all age groups of the population were below 65%, except for the 40-50 age group where it was ≈70%.

Conclusion

The results of this study show a drastic decline in onchocerciasis prevalences after 20 consecutive years of CDTI, indicating a significant progress towards the elimination of the disease in Adamaoua Region. However, additional efforts are needed to increase the population coverage in ivermectin treatment in order to achieve the elimination of the disease in this Region.

Keywords: Onchocerciasis - Ivermectin - CDTI - Elimination - Adamaoua Region - Cameroon.

AUTHOR SUMMARY

Onchocerciasis control is mainly based on mass treatment of populations with an annual dose of ivermectin. At the early implementation stage of onchocerciasis control activities, it was suggested that an onchocerciasis-endemic community using the community-directed treatment with ivermectin (CDTI) approach should become free of the disease after 15 years, a period corresponding to the maximum lifespan of the adult worm in humans. CDTI contributed to eliminate onchocerciasis in few foci in East and West African countries (e.g : Uganda, Mali, Niger, Senegal and Nigeria). In Central Africa, the elimination of this disease remains uncertain after 20 consecutive years of mass treatment in certain foci. The data presented in this study are very promising, indicating that the elimination of onchocerciasis through the CDTI approach may be possible in Adamaoua region of Cameroon. In order to achieve the objective of eliminating onchocerciasis in the near future in this part of the country, efforts of population in invermectin treatment are essential. This will involve active raising awareness among men and women of all eligible age groups of the population in Adamaoua region.

1. INTRODUCTION

Also known as "*River Blindness*"; onchocerciasis is caused by a parasitic worm *Onchocerca volvulus* transmitted from person to person through bites of blackflies belonging to the genus *Simulium.* Over the course of few months to one year, the transmitted L_3 infective stages of the parasite molt twice and develop into sexually mature adult. Female worms live in fibrous nodules of the skin where they remain sessile, while males migrate between the nodules to mate with settled females. Both female and male adult worms live between 2 and 15 years with an average life expectancy of 10 years [**1**, **2**]**.** During this period, patent females release between 700 and 1,500 microfilariae (mf) per day [**1,3**], which predominantly live in the skin for 3 to 5 years causing lesions including intense itching, thickening, skin depigmentation, and loss of its elasticity. Other clinical manifestations such as hanging groin, skin atrophy, papular onchodermatitis and chronic papular onchodermatitis, lichenified onchodermatitis are commonly reported [**4**]**.** The microfilaria (mf) can migrate throughout the intercellular fluid and reach the eyes causing ocular manifestations which can lead to irreversible blindness

[**5**]. Although the physiopathology is not well known, several studies have mentioned links between onchocerciasis, epilepsy and nodding syndrome [**3, 6, 7, 8, 9, 10**].

Onchocerciasis occurs in 35 countries in the world with Sub-Sahara African (SSA) countries carrying over 99% of all cases in the world [**11,12,13,14**]. Historical data from SSA show a bleak picture of this disease in several endemic villages where prevalences of infected individuals sometimes exceeded 80%, and the rates of onchocercal blindness around 10% [**15, 16, 17**]. In regard with the gravity of this severe infectious disease, private, public, national and international health authorities/agencies have decided to reduce its prevalence and transmission, then its elimination in affected countries [**18, 19**]. Since then, the control of the disease has been based on 2 major large-scale strategies including (1) vector control targeting *Simulium* blackflies and (2) parasite control targeting *O. volvulus* in human. These two strategies have been implemented at large scale in SSA to alleviate the burden of the disease in human populations. The first was implemented in West Africa between 1974 - 2002 as a core activity of Onchocerciasis Control Programme (OCP) [**15**]. The second strategy targeting parasite control was proposed with the discovery of a safe and effective microfilaricide, ivermectin known under the brand name Mectizan [**20**]. Since 1987 this drug is freely provided by the pharmaceutical company Merck & Co. [**21**]. The availability of ivermectin in sufficient quantities has enabled to continue the control of onchocerciasis in West Africa and its extension in endemic countries of Central and East Africa under the management of African Programme for Onchocerciasis Control (APOC) [**22, 23**]. The APOC adopted Community-Directed Treatment with Ivermectin (CDTI) as a core strategy with the objective to establish a mechanism for sustained delivery of an annual dose of ivermectin to the entire eligible populations in meso- and hyperendemic communities [**24, 25**]. Based on the lifespan of adult worms in humans, the expectation of eliminating onchocerciasis after 15 consecutive years was a convincing idea for testing vector control in West Africa and implementing CDTI in all endemic countries in SSA [**26**].

However, in countries where vector control or CDTI have been successful, the duration of implementation of each strategy was at least 15 years, corresponding to the maximum lifespan of O. volvulus adult worm in human [**27**]. Evidences of onchocerciasis elimination through CDTI approach in some foci of Latin America and SSA [**28, 29, 30, 31, 32, 33, 34, 35**] have supported the idea of shifting from the control to elimination of the disease [**36**]. Besides, the evidences, it is increasingly recognized that onchocerciasis is no longer a

public health or socio-economic concern after 15 to 20 successive years of CDTI in some foci in West Africa [**37**]. In Central Africa, such a statement remains anecdotal after 20 years of CDTI, without any hope or evidence of eliminating the disease.

The 1998-2002 REMO surveys conducted in Cameroon resulted in the eligibility of this Adamaoua region for ivermectin treatment. The CDTI was launched in 1999 in part of the region (Adamaoua II) and was extended in the whole in 2003. Epidemiological assessments conducted after the CDTI implementation in some endemic zones in Cameroon demonstrated that the transmission of onchocerciasis is still significantly ongoing even in foci where CDTI has been implemented for more than 15 years [**35, 38, 39**]. Those conducted by APOC in 2010-2013 in 7 CDTI projects in Cameroon showed that elimination of onchocerciasis was feasible in the short term in the Adamaoua II project based on the decline in microfilarial prevalences noted in the region [**37**]. Since then, the Adamaoua Region, like all the other regions in Cameroon, has been receiving mass treatments of ivermectin every year. Apart from this 2010-2013 epidemiological survey conducted in 9 villages in part of Adamaoua Region (Adamaoua II), no other survey covering all the region has ever been carried out. This paper reports the situation of onchocerciasis prevalence in the Adamaoua Region after 20 consecutive years of mass treatment of populations with ivermectin.

2. Materials and methods

2.1 Study area

This study was conducted in the Adamaoua Region of Cameroon located between 7°20'N and 13°01'E. The region covers a surface area of about 62,000 km^2, with altitude ranging between 900 m and 1500 m. Geographically, Adamaoua region is situated in the transition zone between the humid equatorial climate of the South and the tropical dry sudano-sahelian climate of the North, influenced by the Adamaoua Plateau [**40**]. The rainy season extends from April to October and annual rainfall is 1,500 mm [**41**]. The dry season occurs between November to March with a mean annual temperature of 20-26°C [**42**]. Most of the watercourses in Cameroon have their source in the Adamaoua Region, so called the "Watershep of Cameroon". The main rivers of the region include, Mayo Deo, Mbéré and Vina rivers which are of a great importance for human activities such as agriculture and livestock breeding [**43, 44**]. These rivers exhibit in their watercourse, rapids, cascades and

waterfalls favorable to the establishment of *Simulium* blackfly populations. The health map divides the region in 9 health districts (HDs) including Ngaoundéré Rural, Ngaoundéré Urbain, Bankim, Tibati, Banyo, Ngaoundal, Tignère, Djohong and Meiganga. Onchocerciasis is known to be endemic in the Adamaoua Region for around 10 decades [**45**]. A total of 29 communities spread across the 9 HDs were selected, with priority given to those visited during the 1998-2002 Rapid Epidemiological Mapping of Onchocerciasis (REMO) conducted in the region.

2.2 Study design

A cross-sectional survey was conducted between August and September 2020. Data were collected from people living in several communities in the HDs of the Adamaoua Region. In these communities, the collection of biological samples was performed in public places i.e in local health facilities or to a lesser extent in schools for communities without health facilities. In each HD, 2 to 4 communities were prospected in the framework of this study. The selection of the surveyed communities was based on (1) the existence for the community a baseline parasitological data on onchocerciasis from the 1998-2002 REMO studies (2) the high density of human population in certain communities and (3) the location of communities near watercourses with potential *Simulium* breeding sites. The participation in this study by community members was voluntary. However, the inclusion criteria were as follows: (1) be at least 5 years old, (2) reside continuously in the community, (3) for adults, sign an informed consent form during the registration and assent form for children when necessary. Because the age of legal majority in Cameroon is 18, a written consent has been signed by a parent or legal guardian for children aged 5 to 17 years. The study participants' itinerary consisted of 3 stations namely registration, skin biopsy collection and general consultation (**Figure 1**). At the registration station, all adult participants provided written informed consent. Personal information of each participant (gender, age, occupation, permanent residence,) was recorded and a barcode number was assigned to each eligible participant to keep track of the data. At the skin biopsy collection station, 2 superficial skin biopsies were collected from each participant at the left and right posterior iliac crests using a sharp and sterilized 2-mm Holthtype corneoscleral punch. To avoid transmission of potential pathogens, one corneoscleral punch was used per participant for the skin biopsy collection. After use, the corneoscleral punches were subjected to a complete sterilization procedure including 2 stages: (1) immersion for 10 to 15 minutes in bleach solutions of

decreasing concentrations (6, 4 and 2 drops in 100 ml of distilled water) and (2) heating at 120°C for 30 minutes in a portable steam sterilization.
Immediately after collection, each skin biopsy was individually placed into a well of a round-bottom 96-well microtiter plate containing approximately 40µL of physiological saline per well. Plates containing skin biopsies were incubated for 24 hours at room temperature to induce microfilaria emergence [**46**]. At the end of this period, they were transferred to the local field laboratory for skin biopsy sample examination. In the laboratory, the physiological saline contained in each well was pipetted and placed on a slide for observation under a 40x magnification in order to search for and count the microfilariae. At the medical consultation station, the participant was subjected to onchocerciasis nodule palpation which consists of searching subcutaneous nodules over the body and the skin manifestations of the disease. The participant was then interviewed about his ivermectin treatment history. The answers provided were noted on an individual sheet that was submitted to the laboratory for archiving result of skin biopsy examination

2.3 Data analysis

Personal information, nodule palpation and skin snip biopsy data for each participant were recorded in a specially prepared spreadsheet. The main indicators of onchocerciasis including microfilaria (mf) and nodule prevalences were expressed as a percentage i.e number of persons positive divided by the number of persons examined × 100. The arithmetic mean of the mf from the two skin biopsies from each participant was calculated and used as a measure of intensity of infection. The Community microfilarial load (CMFL) was calculated as anti-log $\{(\sum\log(x+1))/n\}$ -1, with **x** being the mean of mf/mg of skin and **n** the number of individuals examined [**47**]. In the framework of this study, the methods described by Coffeng and colleagues [**48**] were used to convert nodule prevalences of the 1999-2001 survey (typically in samples of adult males aged ≥ 20 years) into microfilarial prevalences (in the population aged ≥ 5 years).
The chi-square (χ^2) test was used to compare: (1) the microfilaria and nodule prevalences reported in this study with those of 1998-2002 (baseline) and 2010-2013 (APOC evaluation), (2) and the reported and surveyed coverage rates recorded in 2019 in the HDs of Adamaoua Region. A two-tailed p-value lower than 0.05 was considered statistically significant.

3.Results

3.1 Demographic variables

A total of 4,814 participants from 29 communities selected in the 9 HDs of the

Adamaoua region were enrolled in this survey. Men represented 50.4% (N = 2,424) and women 49.6% (N =2,390). Participants were aged between 5 and 108 years old. The distribution of participants according to the study population by age group and gender is shown in **Figure 2**. Participants aged between 5 and 30 years represent more than half of the study population (58.45%).

3.2 Status of *O. volvulus* infection in the surveyed communities and health districts

Of the 29 communities surveyed during this study, 11 (≈ 38%) from 7 HDs were infested by *O. volvulus* microfilariae (**Table 1**). Of these, four were from Meiganga HD, 2 from Ngaoundal HD and 1 from Bankim, Djohong, Ngaoundéré Rural, Ngaoundéré Urbain and Tibati HDs. No infected community was found in the Banyo and Tignere HDs. The mf prevalence among infected communities ranged from 0.50% [0.02 - 3.23] in Mbakaou (Tibati HD) to 4.34% [1.77-9.63] in Nandeke (Meiganga HD). These mf prevalences were significantly lower ($p < 0.05\%$) compared to that recorded in the 1998-2002 baseline survey where prevalences varied from 36.7% in Alhamissa (Tibati HD) to 87.7% in Djamtari (Tignere HD). Of the 29 communities visited in the 2020 follow-up survey, 22 were previously prospected in the 1998-2002 baseline survey. The variation trend in mf prevalences in these 22 communities from the 1998-2002 baseline survey to the 2020 follow-up (after almost 20 years) is shown in **Figure 3.** Of these 22 communities, 13 (59%) were found free of *O. volvulus* infection and 9 (41%) showed mf prevalences below 5% (**Table 1**) in the 2020 study. In general, the intensity of *O. volvulus* infection characterized by the community microfilarial load (CMFL) was very low (< 0.5 mf/ss) in each of the surveyed communities. The CMFLs assessed in the 2020 survey range from 0.006 mf/ss in Wakwa (Ngaoundere Urbain HD) to 0.129 mf/ss in Nandeke (Meiganga HD). When assessed for participants aged more than 20 years, the CMFLs varied from 0 mf/ss in Mbakaou (Tibati HD) to 0.0716 mf/ss in Zouzami (Meiganga HD) (**Table 1**).

At the level of the HD, the mf and nodule prevalences were estimated by grouping together the communities surveyed in the same HD. The prevalences of microfilariae and nodules recorded in the HDs surveyed in 2020 are shown in **table 2**. Within the HDs of the study region, nodules prevalences varied from 0.00 [0.00 - 0.77] in Meiganga HD to 2.36 [1.32 - 4.11] in Tibati HD. The mf prevalences varied from 0.00 [0.00 - 0.80] % in Banyo and Tignere HDs to 3.40 [2.16 - 5.23] in Meiganga HD. When HDs were grouped together, the nodule

and mf prevalences recorded for the whole region were 0.87[0.64 - 1.19] % and 0.77 [0.54 - 1.07] % respectively (**Table 2**). **Figure 3** shows the mf and nodule prevalences reported in these HDs in 1998-2002, 2010-2013 and 2020 in the study region. Overall, the mf and nodule prevalences reported in this 2020 survey were significantly lower ($p < 0.05$) compared to those recorded in the 1998-2002 and 2010-2013 surveys (**Figure 3 A & B**). Compared to the 2010-2013 study, a slight increase in mf prevalence was observed in Meiganga HD in 2020 (**Figure 3B**). At the community level, the baseline 1998-2002 mf prevalences ranging from 51.1% (Ngaoundal) to 77.1% (Ngaoundére Rural) have significantly varied from 0% (Banyo or Meiganga) to 2.1% (Ngaoundal) in the 2020 follow-up survey ($p < 0.05$) (**Figure 4**).

3.3 Trend in ivermectin treatment and *O. volvulus* infection distribution

Of the 4,814 participants interviewed for ivermectin treatment, ≈ 28% (N = 1,351) representing almost one third of the studied population said they had never treated with ivermectin, while ≈ 72% (N = 3,463) declared having received ivermectin treatment at least once in the past 6 years (2014-2019). The proportion of participants who had never been treated with ivermectin in the surveyed communities ranged from 1% (Djamtari, Tignere HD) to 11% (Mbamti, Banyo HD). Among those who were treated, 86% (N = 2,977) 249 received the last treatment in 2019, 9.7% (N = 336) in 2018, 4% (N = 141) in 2017 and the remaining 0.3% in 2016, 2015 and 2014. Of the 37 individuals tested positive for *O. volvulus* infection out of the 4,814 examined during the 2020 survey, 25 were found among those who had been treated with ivermectin at least once, i.e. 25/3463 (0.72%), and 12 among those who had never been treated (non-compliers), i.e. 12/1351 (0.88%). The mf prevalences in treated and untreated individuals are statistically similar ($\chi^2 = 0.3525$, $p = 0.5527$).

The therapeutic coverage rates (TCRs) reported and those surveyed in 2019 in the HDs of Adamaoua Region are shown in **Figure 5**. The TCRs reported by the National Onchocerciasis Control Programme (NOCP) ranged from 78.8% (Tibati HD) to 83.1% (Djohong HD), while those surveyed ranged from 41.3% (Ngaoundere Urbain HD) to 77.5% (Tibati HD). Overall, the TCRs reported by the NOCP were significantly higher than those surveyed, with the exception in Tibati HD, where the reported coverage rate was similar to that surveyed (**Figure 5**).

In this study, adherence rates of population to ivermectin treatment (i.e the percentage of people who took ivermectin among the eligible population in the community or HD) by gender and age group were also investigated (**Figure 6)**. At the regional level, men and women showed comparable adherence rates during the 2019 ivermectin mass distribution campaign i.e 48.1% (N = 1,433) and 51.9% (N = 1,544) respectively ($p > 0.05\%$). This equality in adherence to treatment was also noted at HD level, with the exception in the Ngaoundéré Rural HD where adherence of men was significantly higher than that of women (**Figure 6**). In general, the adherence rate of the studied population in ivermectin treatment at the level of each surveyed HD was below 65% with an exception in Tibati and Tignere HDs where it was above 65% for the two genders, and only for men in Ngaoundere rural HD (**Figure 6**). When HDs were grouped together, the population adherence rate by age group was shown in **Figure 7**. Overall, all age groups showed adherence rates ≤ 65%, with however an exception in the 40-50 age group where the adherence rate was ≈ 70%.

4. Discussion

The objective of this study was to assess the status of onchocerciasis transmission in the Adamaoua Region after two decades of mass treatment with ivermectin. Overall, the nodule and microfilaria prevalences recorded during this 2020 epidemiological study in the surveyed communities and HDs of the region were below 5% with most of them exhibiting zero mf prevalence. Based on the criteria applied for delineating onchocerciasis foci into endemicity levels, all these communities and HDs may be currently classified as sporadic for onchocerciasis [**49**] or hypo-endemic [**50, 51**] for the disease. The CMFLs assessed according to Remme and Colleagues [**52**] showed less than 1 mf/sb indicating low parasitic loads in human population, with however an important proportion of parasites circulating in the youngest population. The parasitic loads assessed in the surveyed communities were significantly low compared to those reported in the same communities during the 1998-2002 REMO data collection conducted in Cameroon [**53, 54**]. This decline in onchocerciasis intensity has previously been reported in Adamaoua II project zone during the 2013 epidemiological assessments conducted in Cameroon as part of the impact assessment of the CDTI strategy in countries of the African Programme for Onchocerciasis Control [**37**]. The low prevalences of the disease recorded in the surveyed communities is attributed to efforts put in place in controlling the disease through an effective involvement of communities in ivermectin mass treatment during the past 20 consecutive years. In the

context of achieving the elimination of onchocerciasis by 2030, data presented in this study are promising compared to the findings recently reported in other regions of Cameroon [**55, 56, 57**]. The slight increase in mf prevalence noted in Meiganga HD during this 2020 epidemiological study compared to that of the 2010-2013 may be attributed to a decline in the population's adherence to ivermectin treatment. This confirms the low adherence (< 65%) of population in ivermectin treatment noted in most of the surveyed HDs and all age groups of the eligible population. Also, the existence in the prospected communities of systematic non-compliers to ivermectin representing ≈ 4 to 11% in each surveyed communities and accounting for half of the total infected individuals identified may favour the observed increase in disease prevalence in some HDs. Indeed, the non-compliers to ivermectin treatment represent microfilaria reservoirs for the human population, and this may induce active transmission in this environment where microfilaria parasites and blackflies co-exist. Indeed, infective stage (L_3) of these microfilariae spread in the community by *Simulium* blackflies during their blood meals from these non-compliers to individuals free of onchocerciasis. In the savannah zones such as the Adamaoua Region, the spread of these parasitic forms is easier, with the passive movement of blackflies that can reach locations situated tens or even hundreds of kilometres away. Regarding parasite transmission, *Simulium damnosum* s.s, *S. sirbanum* and *S. squamosum* are known as major vectors of onchocerciasis in the Adamaoua Region [**58**]. Although the presence of these vectors in the region needs to be confirmed through an updated taxonomic study on *Simulium* blackflies, we might highlight that these blackfly species exhibit the phenomenon of "limitation" known as one of the factors favoring the transmission of the disease [**59, 60**]. The phenomenon is described as the situation where vectors are efficient even at very low parasite densities [**61**]. The low intensity of the disease expressed by CMFLs (< 1mf/ss) and the limited number of infected individuals reported in this study suggest active transmission of the parasite in this region but at low level. Although the decrease in the prevalence of onchocerciasis is noted in the surveyed communities of the region, it should be mentioned that, the local *O. volvulus* parasite population has not yet been reduced to a level below the transmission breakpoint**.** Data collected during 2010-2013 and 2020 studies from Meiganga reflect this situation and suggest that the transmission of infection in this HD as in others may evolve in a piecemeal fashion, indicating that if no special attention is given in delivering ivermectin to population, a recrudescence of the disease may occur rapidly in this region.

Although the entire Adamaoua region is under ivermectin treatment region, the surveyed therapeutic coverage rates in 2019 reveal that 5/9 of the HDs are far below the minimum rate of 65% recommended by the former APOC in the framework of onchocerciasis control as a public health problem. Overall, the surveyed coverage rates recorded are lower than those reported by the NOCP in 2019. Although resulting from oral statements of participants, these surveyed coverage rates may be considered as more informative and reflect the status in ivermectin mass treatment in the Adamaoua Region.

The coverage rates reported and surveyed in each HD of Adamaoua region were statistically comparable only for the Tibati HD, indicating that the CDTI data provided by the local community members and Health system are in agreement with the participants' statements, which according to WHO confers validation of the CDTI strategy in this HD [**62**]. Indeed, in the other HDs, the therapeutic coverage rates reported by NOCP in 2019 would have been for the most part overestimated at different levels of the recording system used in documenting ivermectin treatment. The reasons for overestimating adherence rates of populations to ivermectin treatment include : (1) the justification of the ability to carry out the task, (2) the renewal of the confidence of the hierarchy and (3) the hope of obtaining a consequent financial or material compensation. The low surveyed therapeutic coverage rates reported in 2019 confirms the poor adherence of the eligible population in ivermectin treatment. This observation is a key information to consider for the elimination of the onchocerciasis in Adamaoua Region. Such a situation calls for additional efforts in delivering ivermectin in the region through increased sensitization of local populations. However, combining the ivermectin treatment with complementary strategies such vector control, enhanced CDTI, community-directed treatment (CDT) with drug combinations or new drugs, and test-and-treat (TNT) [**63**], may accelerate the elimination of onchocerciasis in this region.

Data reported in this study are close to the threshold of the elimination previously recommended by the former APOC i.e [**64**] (1) less than 5% prevalence in all surveyed communities and (2) less than 1% in 90% of surveyed communities. All the 29 communities surveyed had mf prevalence below 5% and 70% had mf prevalence below 1%. The current profile of the disease indicates low mf prevalences (< 5%) and CMFLs (< 1mf/ss) across the surveyed communities of the Adamaoua Region. These data are in agreement with WHO guidelines [**65**], confirming that onchocerciasis is no longer a public health problem in this

region. However, to better characterize the profile of the disease in Adamaoua region, it would be advisable to (1) carry out investigations in several other communities not included in this study, (2) increase the size of the study population and (3) and above all to carry out OV16 tests to demonstrate children's exposure to the infection, as recommended by the WHO. Onchocerciasis is endemic in all regions of Cameroon and its elimination should start in one region and spread to others. The data reported in this study suggest that Adamaoua may be the first region where onchocerciasis elimination should start and be effective in the whole country. Indeed, the Adamaoua Region is known as the water tower of Cameroon, where many rivers especially those infested by blackflies, have their source. Starting the elimination of the disease in this region and extending it to others would be a step towards ridding the country of *O. volvulus* infection. The Adamaoua Region is geographically the transition zone between the southern and northern regions of Cameroon i.e., the southern forest and the northern savannah areas. The fight against *Onchocerca volvulus* infection in Adamaoua-North and Adamaoua-South directions could be an effective operational intervention plan to be adopted for the future elimination of onchocerciasis in Cameroon.

5. Conclusion

The results of this study show a drastic decline in the prevalence of onchocerciasis in the Adamaoua Region after 20 consecutive years of CDTI. The mf prevalences recorded indicate that, the disease has reached relatively low levels of endemicity in the surveyed communities suggesting that the interruption of its transmission may be possible in the near future in this part of the country. However, further additional efforts are expected in the population adherence in ivermectin mass treatment to move towards elimination. This goal may be achieved in a very short term between 2025 and 2030 by improving the annual treatment of populations with ivermectin at 85% therapeutic coverage rates. Furthermore, complementary strategies including more in-depth sensitization of community members targeting non-compliers to ivermectin treatment or physical destruction of *Simulium* breeding sites through the community-directed approach (Slash and Clear) would accelerate the elimination of the disease in this part of the country. These data are of great importance to the Ministry of Public Health, in particularly its NOCP, and could serve as a compass for the elimination of onchocerciasis in Cameroon and Central Africa

6. References

1. Karam M, Schulz-Key H, Remme J. Population dynamics of Onchocerca volvulus after 7 to 8 years of vector control in West Africa. Acta Trop. 1987; 44:445–57.

2. Habbema JDF, Plaisier AP, van Oortmarssen GJ, Remme J. Prospective evaluation of onchocerciasis control strategies. Acta Leiden 1990; 59:387–398

3. Gumisiriza N, Mubiru F, Siewe Fodjo JN, Mbonye Kayitale M, Hotterbeekx A, Idro R, Makumbi I et al. Prevalence and incidence of nodding syndrome and other forms of epilepsy in onchocerciasis-endemic areas in northern Uganda after the implementation of onchocerciasis control measures. Infect. Dis. Poverty. 2020; 9, 12.

4. Murdoch ME, Hay RJ, Mackenzie CD, Williams JF, Ghalib HW, Cousens S et al. A clinical classification and grading system of the cutaneous changes in onchocerciasis. Br J Dermatol. 1993 129: 260–269

5. Buck AA. Onchocerciasis: Symptomatology, Pathology, Diagnosis. Geneva, World Health Organization, 1974

6. Druet-Cabanac M, Bertocchi I, Preux PM. Onchocerciasis and epilepsy in the north west region of Central African Republic. Afr J Neurol Sci. 1998 ; 17 :37–8

7. Boussinesq M, Pion SDS, Ngangue D, Kamgno J. Relationship between onchocerciasis and epilepsy: a matched case-control study in the Mbam Valley, Republic of Cameroon. Trans R Soc Trop Med Hyg. 2002 ;96 :537–41.

8. Pion SDS, Kaiser C, Boutros-Toni F, Cournil A, Taylor MM, Meredith SEO, et al. Epilepsy in onchocerciasis endemic areas : systematic review and meta-analysis of population-based surveys. PLoS Negl Trop Dis. 2009 ; 3 : e461

9. Korevaar DA, Visser BJ. Reviewing the evidence on nodding syndrome, a mysterious tropical disorder. Int J Infect Dis. 2013 ;17(3) : e149–52.

10. Kaiser C, Pion SD, Boussinesq M. Case-control studies on the relationship between onchocerciasis and epilepsy: systematic review and meta-analysis. PLoS Negl Trop Dis. 2013 ;7(3) : e2147.

11. World Health Organization: Onchocerciasis and its Control. Report of a WHO Expert Committee on Onchocerciasis Control. Geneva: World Health Organization; 1995:1–104.

12. Crosskey RW, Howard TM. A revised taxonomic and geographical inventory of world blackfl ies (Diptera: Simuliidae), London, The Natural History Museum; 2004.

13. Noma M, Zoure H, Tekle AH, Enyong P, Nwoke BEB, Remme JHF. The geographic distribution of onchocerciasis in the 20 participating countries of the African Programme for Onchocerciasis Control: (1) priority areas for ivermectin treatment. Parasit Vectors 2014 ; 7 :325.

14. Zoure H, Noma M, Tekle AH, Diggle PJ, Giorgi E, Remme JH. The geographic distribution of onchocerciasis in the participating countries of the African Programme for

Onchocerciasis Control: (2) pre-control endemicity levels and estimated number infected. Parasit Vectors, 2014; 7:326

15. Boatin B, Molyneux DH, Hougard JM, Christensen OW, Alley ES, Yameogo et al. Patterns of epidemiology and control of onchocerciasis in west Africa. J Helminthol. 1997 Jun ;71(2) :91-101. doi: 10.1017/s0022149x00015741. PMID : 9192715.

16. Kamgno J, Bouchite B, Baldet T, Folefack G, Godin C, Boussinesq M. Contribution à l'étude de la répartition des filarioses humaines dans la Province de l'Ouest du Cameroun. Bull Soc Pathol Exot. 1997 ; 90(5) :327–30.

17. Hougard JM, Alley ES, Yameogo L, Dadzie KY & Boatin BA () Eliminating onchocerciasis after 14 years of vector control : a proved strategy. Journal of Infectious Diseases 2001 ; 184, 497–503.

18. Eckholm, E. Conquering an ancient scourge ; river blindness. N.Y. Times Mag. 1989; Jan 8, 20–

19. Bump JB, Benton B, Seketeli A, Liese, BH. West Africa: river blindness- success in scaling up and lessons learned. scaling up poverty reduction: A Global Learning Process and Conference Shanghai, 2004; May 25–27,

20. Cump & Omura. Ivermectin, 'Wonder drug' from Japan: the human use perspective. Proc. Jpn. Acad., 2011; Ser. B 87.

21. Thylefors B. The Mectizan Donation Program (MDP). Ann Trop Med Parasitol. 2008;102 Suppl 1:39-44.

22. Amazigo U. The African Programme for Onchocerciasis Control (APOC). Ann Trop Med Parasitol. 2008;102 Suppl 1:19-22.

23. Crump A, Morel CM, Omura S The Onchocerciasis Chronicle: From the Beginning to the End? *Trends in Parasitology*, 2012 ; 28 : 9 10.1016/j.pt.2011.10.001

24. Molyneux and Davies. Onchocerciasis control : moving towards the millennium. *Parasitol Today.* 1997 ; 13 : 418-425

25. World Health Organization. African Programme for Onchocerciasis Control (WHO/APOC)15 years of APOC, African Programme for Onchocerciasis Control. Ouagadougou, Burkina Faso. 2011 ; 1995-2010.

26. Plaisier AP, van Oortmarssen GJ, Remme J, Habbema JDF. The reproductive lifespan of Onchocerca volvulus in West African savanna. Acta Trop. 1991 ; 48, 271–284

27. Lindblade KA, Arana B, Zea-Flores G, Rizzo N, Porter CH, Dominguez A, et al. Elimination of Onchocerca volvulus transmission in the Santa Rosa Focus of Guatemala. Am J Trop Med Hyg. 2007; 77:334–41.

28. Rodríguez-Pérez MA, Fernández-Santos NA, Orozco-Algarra ME, RodríguezAtanacio JA, Domínguez-Vázquez A, Rodríguez-Morales KB, et al. Elimination of onchocerciasis from Mexico. PLoS Negl Trop Dis. 2015 ; 9(7) : e0003922.

29. World Health Organization/Department of Control of Neglected Tropical Diseases. Guidelines for stopping mass drug administration and verifying elimination of human onchocerciasis. Criteria and procedures. 2016; WHO/HTM/NTD/PCT/2016.1.

30. Diawara L, Traore MO, Badji A, Bissan Y, Doumbia K, Goita SF et al. Feasibility of onchocerciasis elimination with ivermectin treatment in endemic foci in Africa: first evidence from studies in mali and senegal. PLoS Negl Trop Dis. 2009; 3(7): e497. 24.

31. Tekle AH, Elhassan E, Isiyaku S, Amazigo UV, Bush S, Noma M et al. Impact of long-term treatment of onchocerciasis with ivermectin in Kaduna State, Nigeria: first evidence of the potential for elimination in the operational area of the African Programme for Onchocerciasis Control. Parasit Vectors. 2012; 5:28.

32. Traore MO, Sarr MD, Badji A, Bissan Y, Diawara L, Doumbia K et al. Proof-ofprinciple of onchocerciasis elimination with ivermectin treatment in endemic foci in Africa: final results of a study in Mali and Senegal. PLoS Negl Trop Dis. 2012; 6(9): e1825.

33. Higazi TB, Zarroug IM, Mohamed HA, Elmubark WA, Deran TC, Aziz N, et al. Interruption of Onchocerca volvulus transmission in the Abu Hamed focus, Sudan. Am J Trop Med Hyg. 2013 Jul ;89(1):51-7. doi: 10.4269/ajtmh.13-0112. Epub 2013 May 20. PMID : 23690554 ; PMCID : PMC3748488.

34. Katabarwa M, Lakwo T, Habomugisha P, Agunyo S, Byamukama E, Oguttu D,et al. Transmission of Onchocerca volvulus by Simulium neavei in Mount Elgon focus of Eastern Uganda has been interrupted. Am J Trop Med Hyg. 2014 Jun ;90(6) :1159-66. doi: 10.4269/ajtmh.13-0501. Epub 2014 Mar 31. PMID : 24686740 ; PMCID : PMC4047747.

35. Lakwo TL, Garms R, Rubaale T, Katabarwa M, Walsh F, Habomugisha P et al. The disappearance of onchocerciasis from the Itwara focus, Western Uganda after elimination of the vector Simulium neavei and 19 years of annual ivermectin treatments. Acta Trop, 2013; 126: 218–221. doi: 10.1016/j.actatropica .02. 016 PMID: 23458325

36. Mackenzie CD, Homeida MM, Hopkins AD and Lawrence JC. Elimination of onchocerciasis from Africa: possible? Trends in Parasitology Jan 2012, Vol. 28, No. 1 doi :10.1016/j.pt.2011.10.003

37. Tekle AH, Zouré HGM, Noma M, et al. Progress towards onchocerciasis elimination in the participating countries of the African program for onchocerciasis control : epidemiological evaluation results. *Infectious Diseases of Poverty*. 2016 ;5 :66

38. Katabarwa MN, Eyamba A, Nwane P, Enyong P, Yaya S, Baldiagaï J et al. Seventeen years of annual distribution of ivermectin has not interrupted onchocerciasis

transmission in North Region, Cameroon. Am J Trop Med Hyg. 2011 Dec; 85(6) :1041-9. doi: 10.4269/ajtmh.2011.11-0333. PMID : 22144441 ; PMCID : PMC3225149.

39. Kamga GR, Dissak-Delon FN, Nana-Djeunga HC, Biholong BD, Ghogomu SM, Souopgui J et al. Important progress towards elimination of onchocerciasis in the West Region of Cameroon. Parasit Vectors. 2017 Aug 3 ;10(1) :373. doi: 10.1186/s13071-017-2301-7. PMID : 28774318 ; PMCID : PMC5543544.

40. Génieux M. Climatologie Du Cameroun, in : Atlas Du Cameroun. ORSTOM, 1958 ; France.

41. Olivry JC. Fleuves et rivières du Cameroun. Monographie hydrologique. ORSTOM, 1986 ; Paris, p. 733.

42. Suchel JB. Les climats du Cameroun. Ph.D Thesis University of Saint-Etienne, 1998; France.

43. Letouzey R. Observation phytogéographique concernant le plateau africain de l'Adamaoua. Adansonia, sect. B, 1969 ; 29 (3) : 321-337.

44. Letouzey R. Notice de la carte phytogéographique du Cameroun à 1/500 000. Vol. IS-S : Domaine sahélien et soudanien. IRA, Cameroun, Institut de la Carte Internationale de la Végétation, 1985 (Toulouse/France), 26 p.

45. Paris Frédéric. Chronique d'une endémie opportuniste. Développement rural et onchocercose au Nord Cameroun (Foyer du Faro). In : Espace, populations, sociétés, 2000-2. Maladies émergentes et reviviscentes. 1944 ; pp. 241-252 ; doi: https://doi.org/10.3406/espos.2000.1944

46. Collins RC, Brandling-Bennett AD, Hollman RB, Campbell CC, Darsie RF. Parasitological diagnosis of onchocerciasis: comparison of incubation media and incubation times for skin snips. Am J Trop Med Hyg. 1980; 29(1):35–41.

47. Hoerauf A, Mand S, Adjei O, Fleischer B, Büttner DW. Depletion of *Wolbachia* endobacteria in *Onchocerca volvulus* by doxycycline and microfilaridermia after ivermectin treatment. *Lancet.* 2001 ; 357 :1415–1416. doi: 10.1016/S0140-6736(00)04581-5.

48. Coffeng LE, Pion SDS, O'Hanlon S, Cousens S, Abiose AO, Fischer PU et al. Onchocerciasis: The Pre-control Association between Prevalence of Palpable Nodules and Skin Microfilariae. PLoS Negl Trop Dis, 2013 ; 7(4): e2168. doi: 10.1371/journal.pntd.0002168

49. McMahon JE, Sowa SIC, Maude GH, Kirkwood BR. Onchocerciasis in Sierra Leone 2: a comparison of forest and savanna villages. Transactions of the Royal Society of Tropical Medicine and Hygiene, 1988 ; 82 (4) 595-600.

50. Prost A, Hervouet JP, Thylefors B. Epidemiologic status of onchocerciasis. Bull World Health Organ. 1979; 57: 655–62

51. Walker M, Stolk WA, Dixon MA, Bottomley C, Diawara L, Traoré MO et al. Modelling the elimination of river blindness using long-term epidemiological and programmatic data from Mali and Senegal. Epidemics. 2017 Mar ;18 :4-15. doi : 10.1016/j.epidem.2017.02.005. PMID : 28279455 ; PMCID : PMC5340858.

52. Remme J, Ba O, Dadzie KY, Karam M. A force-of-infection model for onchocerciasis and its applications in the epidemiological evaluation of the Onchocerciasis Control Programme in the Volta River basin area. Bull World Health Organ. 1986; 64:667–81.

53. Kamgno J, Boussinesq M. Hyperendémicité de la loase dans la plaine Tikar, région de savane arbustive du Cameroun. *Bulletin de la Société de Pathologie Exotique*, 2001, 94 (4), p. 342-346. ISSN 0037-9085.
Identifiant IRD :

54. Noma M, Nwoke BEB, Nutall I, Tambala PA, Enyong P, Namsenmo A. Rapid epidemiological mapping of onchocerciasis (REMO): its application by the African Programme for Onchocerciasis Control (APOC), Annals of Tropical Medicine & Parasitology, 2002; 96:sup1, S29-S39, DOI: 10.1179/000349802125000637

55. Tsapi EM, Togjom FG, Gamago G-A, Pone JW, Teukeng FFD. Prevalence of onchocerciasis after seven years of continuous community-directed treatment with ivermectin in the Ntui health district Centre region Cameroon. Pan Afr Med J. 2020 ;1 :180.

56. Aza'ah RA, Sumo L, Ntonifor NH, Bopda J, Bamou RH, Nana-Djeunga HC. Point prevalence mapping reveals hotspot for onchocerciasis transmission in the Ndikinimeki Health District, Centre Region, Cameroon. Parasit Vectors. 2020 Oct 16 ;13(1) :519. doi: 10.1186/s13071-020-04387-6. PMID : 33066826 ; PMCID : PMC7565768.

57. Forrer A, Wanji S, Obie ED, Nji TM, Hamill L, Ozano K et al. Why onchocerciasis transmission persists after 15 annual ivermectin mass drug administrations in South-West Cameroon. BMJ Glob Health. 2021 Jan ;6(1) : e003248. doi: 10.1136/bmjgh-2020-003248. PMID : 33431378 ; PMCID : PMC7802695.

58. Traore-Lamizana, Monographie sur la cartographie des gîtes larvaires des vecteurs d'onchocercose au Cameroun, p. 1, Carte montrant la distribution des gîtes larvaires de Simulium sp. Document de l'IRD, 1981.

59. Philippon B. Etude de la transmission d'Onchocerca volvulus (Leuckart, 1893) (Nematoda, Onchocercidae) par Sirnuliurn dainriosuin Theobald, 1903 (Diptera, Simuliidae) en Afrique tropicale. Travaux et Documents de l'O.R.S.T.O.M. No. 63. Paris : Office de la Recherche Scientifique et Technique d'Outre-Mer. ORSTOM, France ans; 1977.

60. Demanou M, Enyong P, Pion SD, Basanez MG, Boussinesq M. Experimental studies on the transmission of Onchocerca volvulus by its vector in the sanaga valley

(Cameroon): simulium squamosum B. Intake of microfilariae and their migration to the haemocoel of the vector. Ann Trop Med Parasitol. 2003;97(4):381–402.

61. Amuzu H, Wilson MD, Boakye DA. Studies of Anopheles gambiae sl (Diptera: Culicidae) exhibiting different vectorial capacities in lymphatic filariasis transmission in the Gomoa district, Ghana. Parasit Vectors. 2010 ;3 :85.

62. WHO. 2016. Coverage evaluation surveys for preventive chemotherapy: *field guide for implementation.* World Health Organization, November 2016.

63. Boussinesq M, Fobi G and Kuesel AC, 2017. Alternative treatment strategies to accelerate the elimination of onchocerciasis. Int Health 2018; 10 : i40–i48 doi:10.1093/inthealth/ihx054

64. African Program for Onchocerciasis Control/World Health Organization. Conceptual and operational framework of onchocerciasis elimination with ivermectin treatment. World Health Organization/African Program for Onchocerciasis Control, 2010. WHO/APOC/MG/10.1, JAF 16.6 II.

65. World Health Organization. Report of the external mid-term evaluation of the African Programme for Onchocerciasis Control; JAF 16.8. 2010. http://who.int/apoc/MidtermEvaluation_29Oct2010_final_printed.pdf. Accessed 21 Mar 2018.

Declaration

The data we have presented in this paper may be free of access to the general public and should not be restricted. They should be accessible on the Internet without any particular requirements, as they will help to inform the scientific community about the situation of onchocerciasis in the Adamaoua region. All data are in the manuscript and/or supporting information files

Ethics approval and consent to participate

The study protocol was approved by the Cameroon National Ethics Committee (N◦2020/08/1286/CE/CNERSH/SP), on 21 August 2020. Each study participant aged 18 and above personally affixed both thumbprints to the informed consent form presented to them to confirm their participation in the study. Parental consent was obtained for participants aged 5 to 17 years

COMPETING INTERESTS

The authors declare that they have no competing interests.

FUNDING

This study was funded by kwF, through the Project for Control of Neglected Tropical Diseases in Central Africa (MTN/OCEAC) Internal BMZ N° 2015. 69.227+ 2016. 68797. This funding has been allocated to the NGDO International Eye Foundation, which supports the

Ministry of Public Health in the fight against onchocerciasis in the Adamaoua Region. The funders had no role in study design, data collection, data analysis, data interpretation, writing of the report, or the decision to submit for publication

AUTHORS' CONTRIBUTION

PN, HND and JK designed the study protocol. PN, NZT, AD, GRN, MT, JB, SB, AB, AN, HN, PM, GM collected data on the field. PN, HND and YEN supervised and monitored data collection and management. PN, HND and JK reviewed the results and contributed to their interpretation. PN, FNB and YEN conducted data analysis, PN a prepared the first draft of the manuscript. All authors participated in the interpretation of results and revisions of the manuscript. All authors read and approved the final manuscript.

DATA AVAILABILITY

All data are in the manuscript and/or supporting information files

ACKNOWLEDGMENTS

The authors are grateful to the Regional Health Delegate of Adamaoua Region, all health staff at the levels of the health districts and health areas, the traditional chiefs and their respective populations for their active involvement for data collection.

AUTHORS' INFORMATION

PN, Email: philino07@yahoo.fr, Medical Entomologist, Research Scientist at the High Institute of Scientific and Medical Research (ISM) Yaoundé Cameroon.

HND, Email: nanadjeunga@crfilmt.org, Molecular Parasitologist, at the High Institute of Scientific and Medical Research (ISM) Yaoundé Cameroon

AD, Email: domche_andre@yahoo.fr, Medical Entomologist, Ph.D student at the University of Yaoundé I/Research assistant at the High Institute of Scientific and Medical Research (ISM) Yaoundé Cameroon.

GRN, Email: njitchouang@yahoo.fr, Biochemist, at the High Institute of Scientific and Medical Research (ISM) Yaoundé Cameroon.

JK, Email: kamgno@crfilmt.org, Epidemiologist, Director of the High Institute of Scientific and Medical Research (ISM) Yaoundé Cameroon

YEN: Email: emalio2002@yahoo.fr, Statistician at the High Institute of Scientific and Medical Research (ISM) Yaoundé Cameroon

NZT: Email: narcissenzunetoche@yahoo.fr, Biochemist, Ph.D student at the University of Yaoundé I/Research assistant at the High Institute of Scientific and Medical Research (ISM) Yaoundé Cameroon.

MT: Email: augustaflore@yahoo.fr, Ph.D student at the University of Buea I/Research assistant at the High Institute of Scientific and Medical Research (ISM) Yaoundé Cameroon.

JB: f_bopda@yahoo.fr, laboratory technician at the High Institute of Scientific and Medical Research (ISM) Yaoundé Cameroon.

FNB: Email: fesuhbe@yahoo.co.uk,

SB: Email: mbickmensteve@yahoo.fr; laboratory technician at the High Institute of Scientific and Medical Research (ISM) Yaoundé Cameroon

AB: Email: aubinsantos1988@yahoo.fr; laboratory technician at the High Institute of Scientific and Medical Research (ISM) Yaoundé Cameroon

AN: Email: nkouelle@yahoo.fr; Project Manager at International Eye Foundation

HO: Email: obamahonore@yahoo.fr, Data manager at the National Onchocerciasis Control Program/ Ministry of Public Health

PM: Email: mepafel2001@yahoo.fr, Data manager at the National Onchocerciasis Control Program/ Ministry of Public Health

GNA: Email gb_nko@yahoo.fr, Head of Department of Neglected Tropical Diseases/ Ministry of Public Health

BDB: biholong_di@yahoo.fr; Permanent secretary of National Onchocercisasis Control Program

Table 1 : Mf prevalences in the communities prospected during the 1998-2002 and 2020 epidemiological surveys conducted in Adamaoua Region region.

		Geographic coordinates		Baseline survey (1998-2002)		Follow up survey (2020)				
**Health District**	**Community**	**Latitude**	**Longitude**	**# of persons examined**	**Mf prevalence (1998-2002)**	**# of persons examined**	**Mf preva-lence [95%IC] (2020)**	**p-value**	**CMFL (All)**	**CMFL (+20yrs)**
Bankim	Mgbandji*	5°59'22.5''	11°16'36.5''	30	80.2	87	1.14 [0.06 - 7.13]	< 5%	0.023	0.035
	Koumtchoum*	6°26'81.8''	11°12'14.8''	30	38.9	227	0	< 5%	-	-
	Moinkoing*	6°02'58.3''	11°24'40.2''	32	55.7	100	0	< 5%	-	-
Banyo	Mayo Dinga*	6°25'18.2''	11°34'15.0''	30	60.3	173	0	< 5%	-	-
	Mbamti*	6°41'02.7''	12°04'00.0''	32	59.3	180	0	< 5%	-	-
	Yimbere*	6°27'35.3''	11°34'269''	30	50.9	239	0	< 5%	-	-
Djohong	Gbatoua*	6°50'42.6"	14°42'56.4"	33	65.7	146	0.68 [0.03 - 4.32]	< 5%	0.008	0.013
	Yamba*	7°06'21.0"	15°12'20.8"	30	71.0	129	0	< 5%	-	-
Meiganga	Korekoni*	6°08'09.3"	14°33'48.0"	33	55.4	225	4.00 [1.96 - 7.70]	< 5%	0.127	0.051
	Lokoti	6°22'00.0"	14°20'00.0"	43	-	89	1.12 [0.05 - 6.97]	-	0.022	0.022
	Zouzami*	6°37'08.3"	14°32'20.0"	30	80.3	168	2.97 [1.10 - 7.17]	< 5%	0.049	0.071
	Nandeke*	6°27'09.7"	14°13'47.1"	38	56.3	138	4.34 [1.77 - 9.63]	< 5%	0.129	0.047
Ngaoundal	Boy-Baya	6°25'26.4"	13°23'10.7"	31	-	56	3.57 [0.62 - 13.38]	-	0.048	0.048
	Bagodo*	6°25'26.4"	13°23'10.7"	50	55.6	110	0	< 5%	-	-
	Ngaoundal*	6°28'06.0"	13°16'09.4"	31	46.6	206	2.91 [1.18 - 6.52]	< 5%	0.050	0.050
Ngaoundere Rural	Nganha*	7°26'08.8"	13°55'43.2"	30	80.2	169	0.59 [0.03 - 3.75]	< 5%	0.010	0.010
	Bakari Bata*	6°55'41.9''	14°35'51.7"	33	59.1	213	0	< 5%	-	-
	Berem*	7°33'03.7''	13°55'28.4''	30	86.7	299	0	< 5%	-	-
	Nyassar*	7°31'40.1''	14°01''43.7''	30	82.5	281	0	< 5%	-	-
Ngaoundere Urbain	Wakwa*	7°16'05.8''	13°33'01.6''	30	66.0	95	4.21 [1.35 - 11.03]	< 5%	0.026	0.051
	Gadamabanga	7°21'37.7"	13°35'05.5"	32	-	78	0	-	-	-
	Mballang	7°18'00.8''	13°44'30.2''	30	-	110	0	-	-	-
Tibati	Mbakaou*	6°18'13.9''	12°47'48,0''	52	67.4	197	0.50 [0.02 - 3.23]	< 5%	0.006	0
	Alhamissa*	6°30'01,9''	12°41'58.9''	30	36.7	113	0	< 5%	-	-
	Djombi	6°41'28.8''	12°36'18.4''	33	-	240	0	-	-	-
Tignere	Libong	7°23'21.5''	13°00'06.4''	30	-	171	0	-	-	-
	Mayo-Djarandi	7°22'05.0''	12°38'59.3''	30	-	175	0	-	-	-
	Mayo-Kaloua*	7°05'13.7''	12°28'15.0''	23	74.1	187	0	< 5%	-	-
	Djamtari*	7°42'03.4''	12°15'01.9''	36	87.7	213	0	< 5%	-	-

*Communty prospected in 1998-2002 and 2020 ; **#** : number ; **Mf**: microfilaria; **-**: No data available

664 **Table 2** : Microfilaria and nodule prevalences recorded in the health districts of the Adamaoua Region

Health District	# of persons examined	Prevalence	
		Nodule	Mf
Bankim	414	0.96 [0.31- 2.63]	0.24 [0.01 - 1.55]
Banyo	592	1.18 [0.52 - 2.53]	0.00 [0.00 - 0.80]
Djohong	275	2.18 [0.89 - 4.92]	0.36 [0.02 - 2.33]
Meiganga	620	0.00 [0.00 - 0.77]	3.40 [2.16 - 5.23]
Ngaoundal	372	0.53 [0.09 - 2.14]	2.15 [1.00 - 4.36]
Ngaoundere rural	962	0.10 [0.00 - 0.67]	0.10 [0.00 - 0.67]
Ngaoundere urbain	283	0.71 [0.12 - 2.81]	1.41 [0.45 - 3.82]
Tibati	550	2.36 [1.32 - 4.11]	0.18 [0.01 - 1.17]
Tignere	764	0.94 [0.41 - 2.01]	0.00 [0.00 - 0.64]
**TOTAL**	**4,814**	**0.87 [0.64 - 1.19]**	**0.77 [0.54 - 1.07**]

665 **Mf**: microfilaria

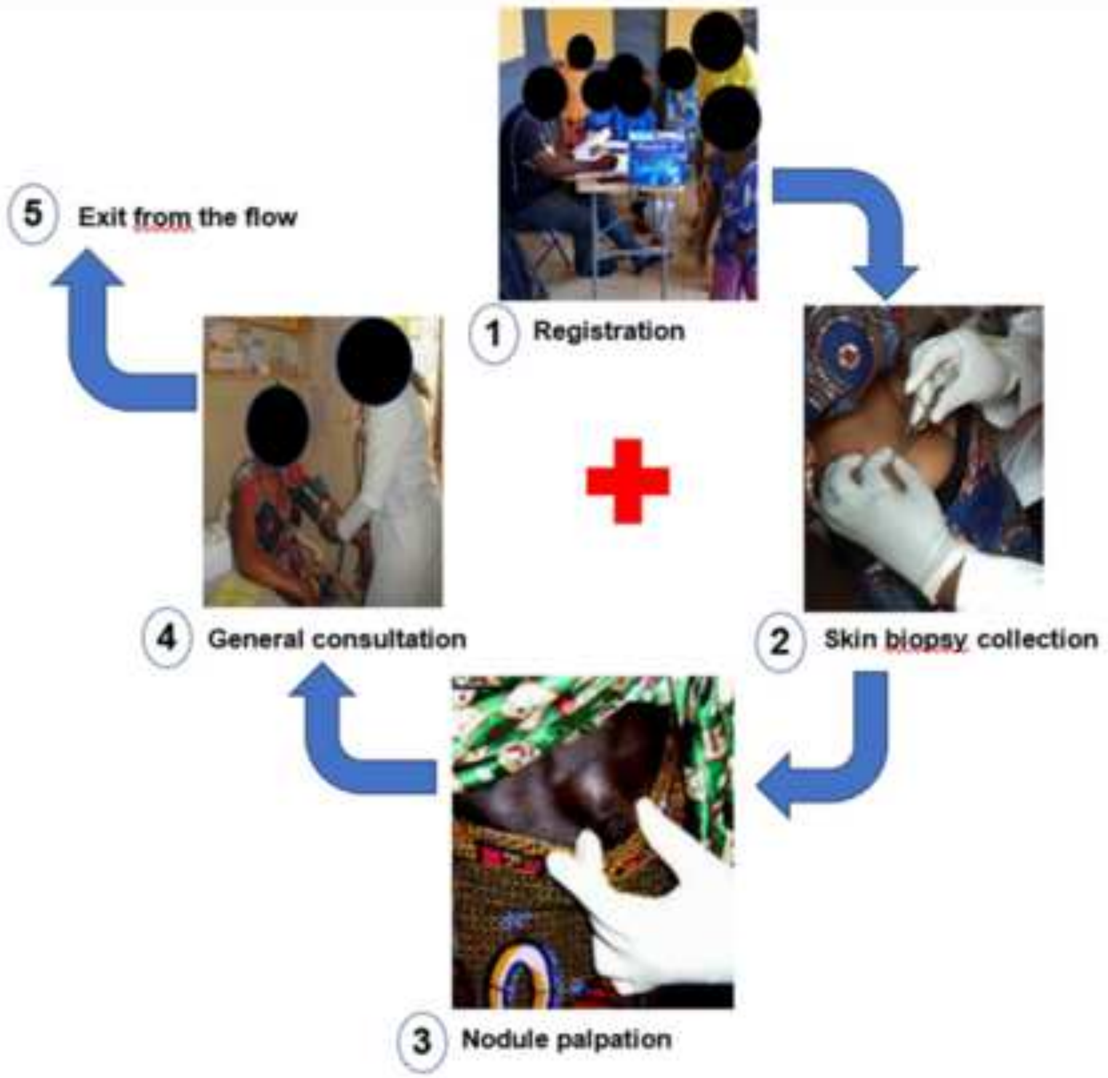

Figure 1. Participant progress flow during data collection procedure

Figure Click here to access/download;Figure;Figure-2 (1).tiff

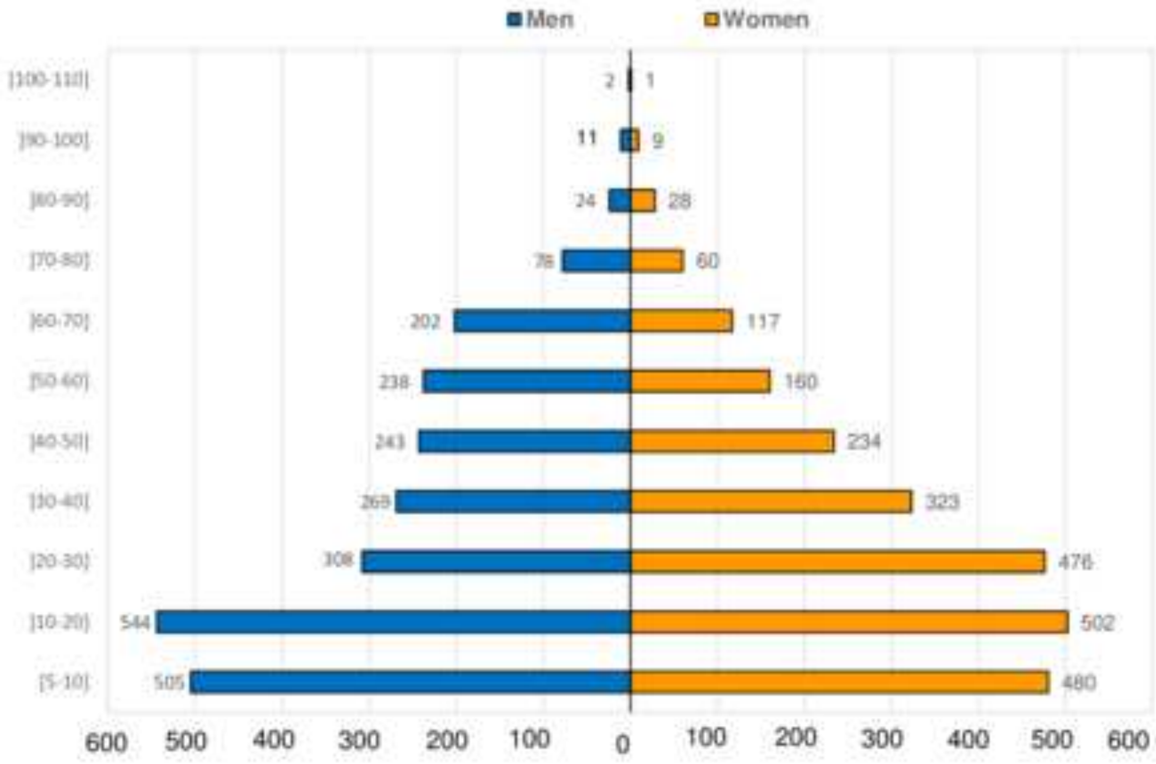

Figure 2 : Distribution of the study population according to gender and age groups

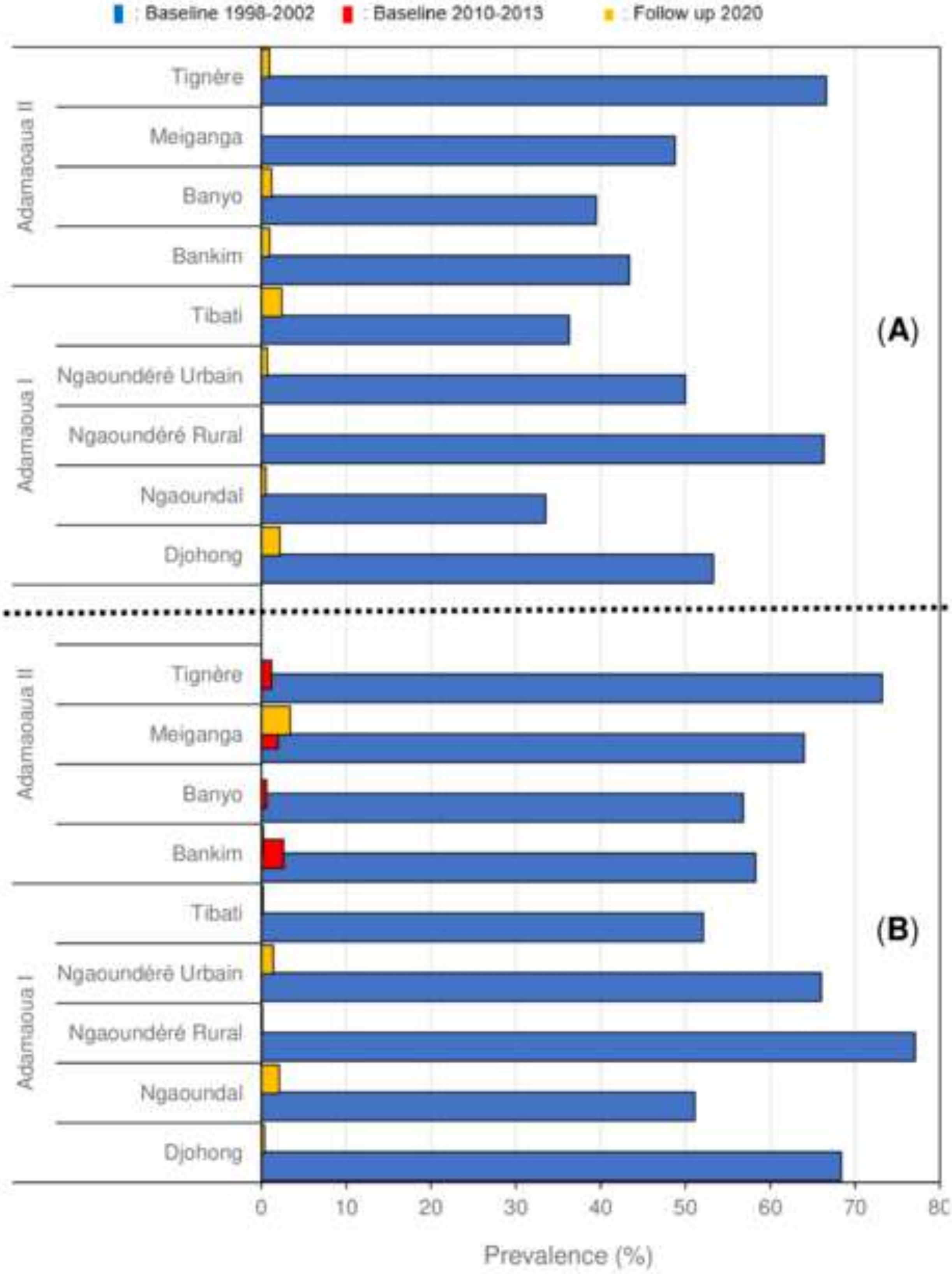

Figure 3 : Nodule (**A**) and microfilaria (**B**) prevalences recorded in surveyed Health Districts

Figure
Click here to access/download;Figure;Figure-4 (1).tiff

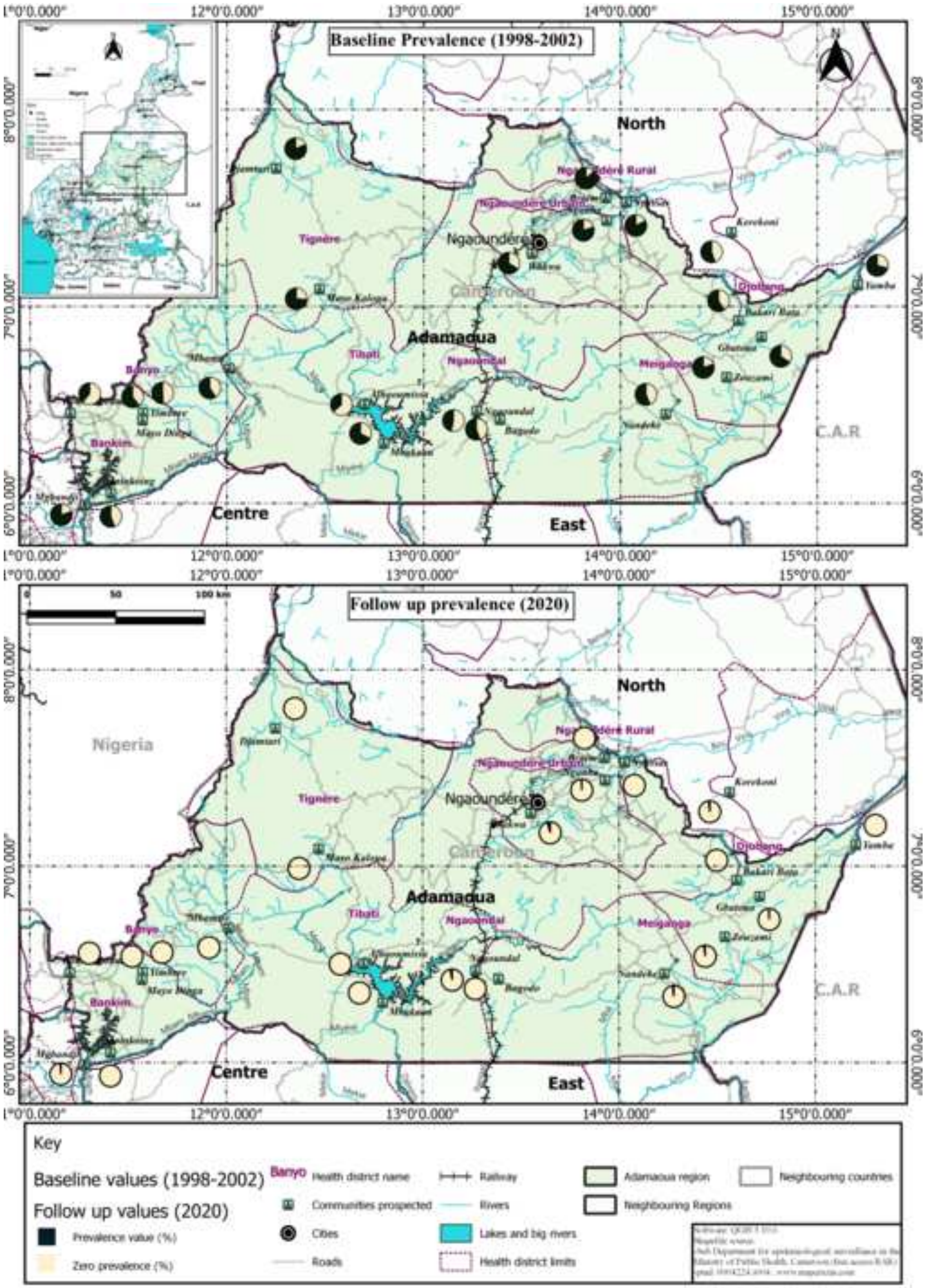

Figure

Click here to access/download;Figure;Figure-5 (1).tiff

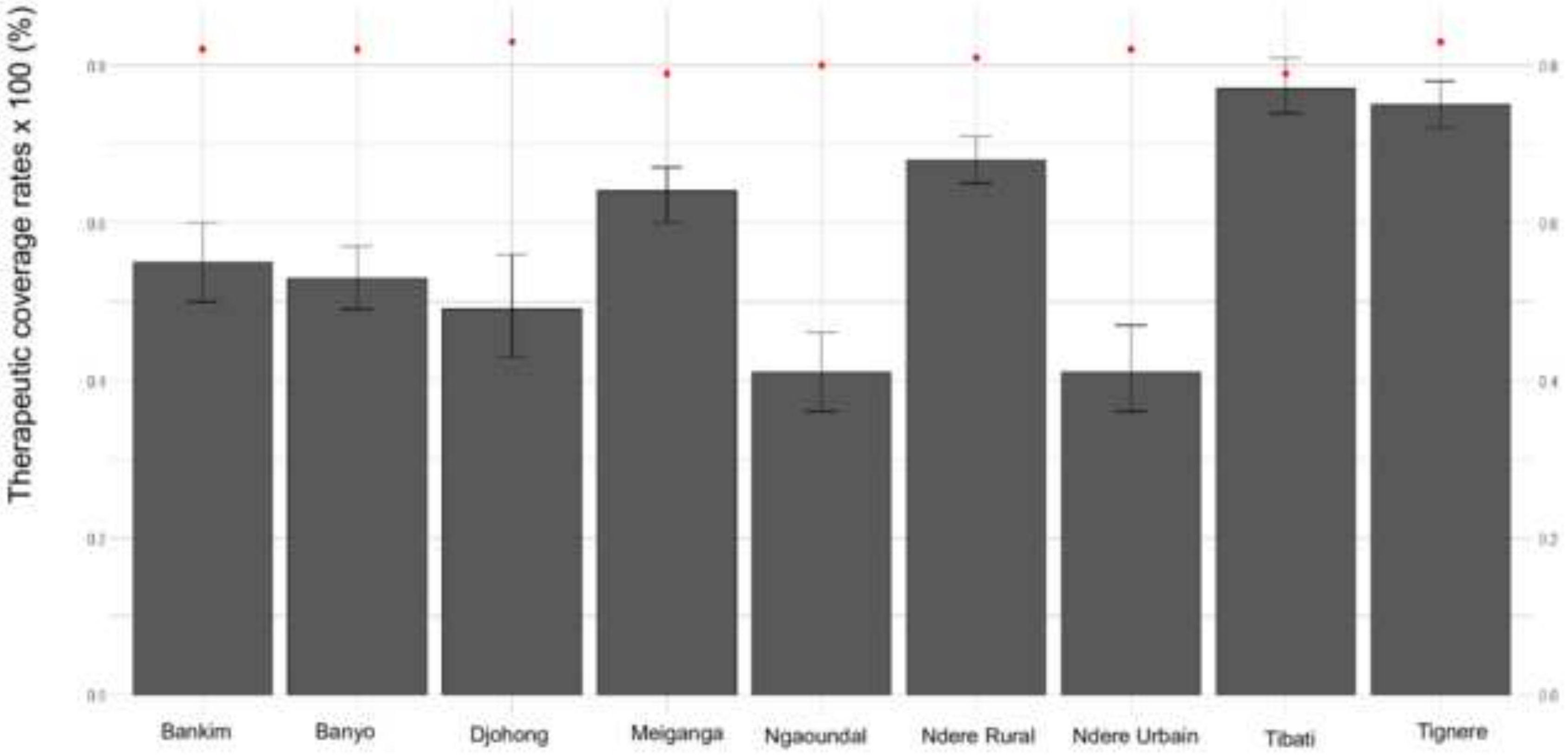

Figure 5 : Therapeutic coverage rates reported (**red dot**) and surveyed (**histrogram bar**) in the health districts of Adamaoua Region in 2019.

Ndere : Ngaoundere

 Click here to access/download;Figure;Figure-6 (1).tiff

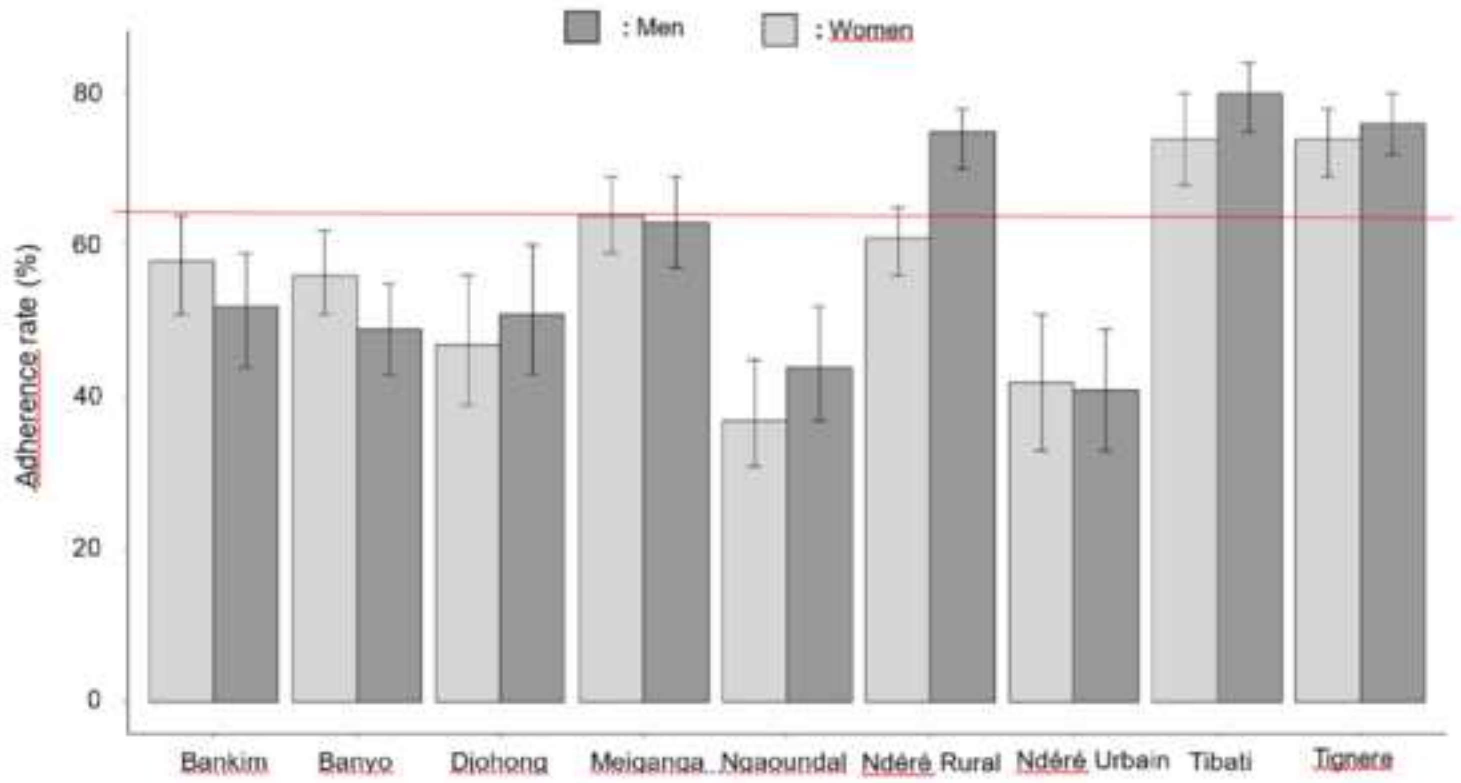

Figure 6 : Population adherence rates by gender to ivermectin treatment during the 2019 mass distribution in the 9 health districts of Adamaoua Region
Ndéré : Ngaoundéré

 Click here to access/download;Figure;Figure-7(1).tiff

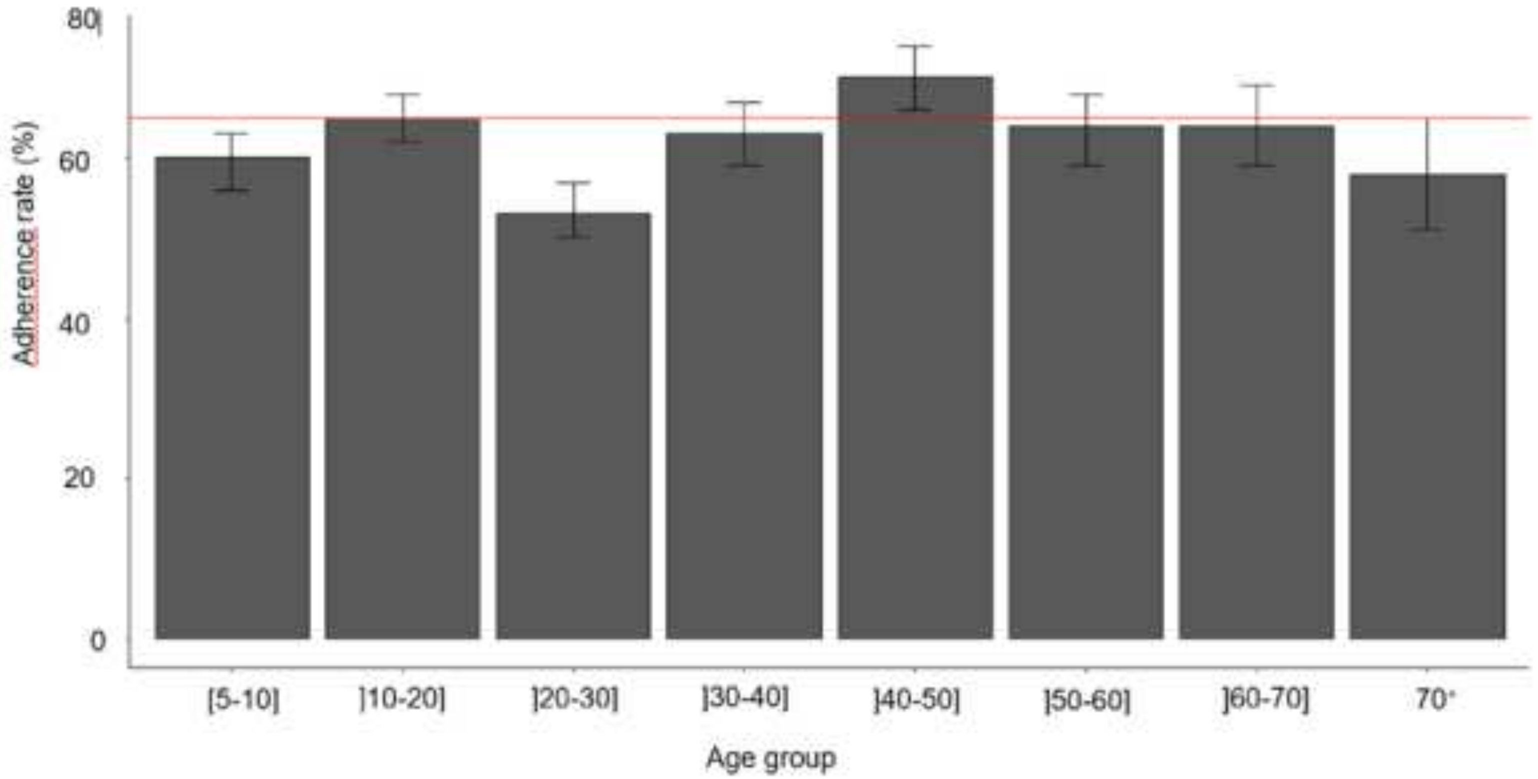

Figure 7 : Population adherence rate according to age groups during the 2018 ivermectin mass treatment in the 9 health districts of the Adamaoua Region

Answers to questions of Reviewer N°1

We would like to thank the reviewers who have examined the scientific content of this work, with the aim of improving it and publishing it in your journal. We present here the responses to Revewer N°1's suggestions and comments in the order in which they appeared in the pdf manuscript he shared with us.

Abstract

Introduction

We have deleted the first 2 sentences of the introduction as requested and replaced them with the following ones « Significant progress has been made in onchocerciasis control through mass distribution of ivermectin among affected human populations, fostering optimism for disease elimination. However, despite these considerable advances, the elimination of the disease remains a major challenge in many African foci»

Material and methods

We replaced the ANOVA and Welch's unequal variances t-test not used for the analyses with the chi-square (χ2) statistic test.

Results

We have taken into account the suggestions made in the "Results" section.

Author summary

We have revised this section according to the recommendations of the reviewer

1. Introduction

Line 79 : We have revised this sentence as follows : Onchocerciasis occurs in 35 countries in the world with Sub-Sahara African (SSA) countries carrying over 99% of all cases in the world
Lines 84- 86: The sentence has been revised as follows: In regard with the gravity of this severe infectious disease, private, public, national and international health authorities/agencies have decided to reduce its prevalence and transmission, then its elimination in affected countries
Line 86 : We deleted thusfare in the following sentence

Lines 94-97 : The sentence was changed as follows : The availability of ivermectin in sufficient quantities has enabled to continue the control of onchocerciasis in West Africa and its extension in endemic countries of Central and East Africa under the management of African Programme for Onchocerciasis Control (APOC) [**22, 23**].
Lines 103-106: The sentence was changed as follows: However, in countries where vector control or CDTI have been successful, the duration of implementation of each strategy was at least 15 years, corresponding to the maximum lifespan of O. volvulus adult worm in human

Lines 112-116 : The sentence was changed as follows : The 1998-2002 REMO surveys conducted in Cameroon resulted in the eligibility of this Adamaoua region for Ivermectin

treatment. The CDTI was launched in 1999 in part of the region (Adamaoua II) and was extended in the whole in 2003

Lines 119-121 : We have added the history of treatment and epidemiological investigations to the text. The sentence was changed as follows "Apart from this 2010-2013 epidemiological survey conducted in 9 villages in part of Adamaoua Region (Adamaoua II), no other survey covering all the region has ever been carried out

2. Material and methods

Lines 124-125: The sentence was revised as follows: This study was conducted in the Adamaoua Region of Cameroon located between 7°20'N 13°01'E

Lines 124-125: The rainy season extends from April to October and annual rainfall is 1,500 mm [**41**]. The dry season occurs between November to March with a mean annual temperature of 20-26°C.

Line 133: We replaced "Water Tower" by "WaterShep"

Line 143: Include general comments for the manuscript

Lines 154-155: The sentence was revised as follows: However, the inclusion criteria were as follows: (1) be at least 5 years old, (2) reside continuously in the community, (3) for adults, sign an informed consent form during the registration and assent form for children when necessary

Lines 156-159: The sentence was revised as follows: The study participants' itinerary consisted of 3 stations namely registration, skin biopsy collection and general consultation (**Figure 1**).
Lines 160-161: We deleted the sentence as suggested

Lines 160-161: We replaced "function" by "occupation"

Lines 175-178: The sentence was revised as follows: In the laboratory, the physiological saline contained in each well was pipetted and placed on a slide for observation under a 40x magnification in order to search for and count the microfilariae

Lines 182-184: The sentence was revised as follows: The answers provided were noted on an individual sheet that was submitted to the laboratory for archiving result of skin biopsy examination

Line 186: Please we kept "Personal information" instead of demographic information

Line 187 : We replaced "Excel file" by "spreadsheet"

Line 197 : We have maintained the Chi-square (χ^2) test in the manuscript as a method of statistical analysis.

3. Results

Line 202 : We have replaced “data” par “variable”

Lines 205-206 : We deleted “with a sex ratio of 1 :1” and “medium” in brackets

Line 210-211 : We have revised the sentence as follows : Of the 29 communities surveyed during this study, 11 (≈ 38%) from 7 HDs were infested by *O. volvulus* microfilariae (**Table 1**)

Lines 211- 212: The sentence was revised as follows: Of these, four were from Meiganga HD, 2 from Ngaoundal HD and 1 from Bankim, Djohong, Ngaoundéré Rural, Ngaoundéré Urbain and Tibati HDs

Line 216 : The statistical method (Chi-square (χ^2) test) used has been specified in the text and we added a column of p-values in the table 1

Lines 225-228 : This part of the results has been taken into account in the “discussion section”.

Lines 230-231 : The sentence has been revised as follows : The prevalences of microfilariae and nodules recorded in the HDs surveyed in 2020 are shown in table 2.

Lines 233-241 : **Thank you very much for the observation**. We revised all this paragraph as follows : **Figure 3** shows the mf and nodule prevalences reported in these HDs in 1998-2002, 2010-2013 and 2020 in the study region. Overall, the mf and nodule prevalences reported in this 2020 survey were significantly lower ($p < 0.05$) compared to those recorded in the 1998-2002 and 2010-2013 surveys (**Figure 3 A & B**). Compared to the 2010-2013 study, a slight increase in mf prevalence was observed in Meiganga HD in 2020 (**Figure 3B**). At the community level, the baseline 1998-2002 mf prevalences ranging from 51.1% (Ngaoundal) to 77.1% (Ngaoundére Rural) have significantly varied from 0% (Banyo or Meiganga) to 2.1% (Ngaoundal) in the 2020 follow-up survey ($p < 0.05$) (**Figure 4**).

Lines 244-252 : **Trend in ivermectin treatment and *O. volvulus* infection distribution**
We have revised this paragraph according to the reviewer's suggestions. The paragraph now reads as follows : Of the 4,814 participants interviewed for ivermectin treatment, ≈ 28% (N = 1,351) representing almost one third of the studied population said they had never treated with ivermectin, while ≈ 72% (N = 3,463) declared having received ivermectin treatment at least once in the past 6 years (2014-2019). The proportion of participants who had never been treated with ivermectin in the surveyed communities ranged from 1% (Djamtari, Tignere HD) to 11% (Mbamti, Banyo HD). Among those who were treated, 86% (N = 2,977) 249 received the last treatment in 2019, 9.7% (N = 336) in 2018, 4% (N = 141) in 2017 and the remaining 0.3% in 2016, 2015 and 2014. Of the 37 individuals tested positive for *O. volvulus* infection out of the 4,814 examined during the 2020 survey, 25 were found among those who had been treated with ivermectin at least once, i.e. 25/3463 (0.72%), and 12 among those who had never been treated (non-compliers), i.e. 12/1351 (0.88%). The mf prevalences in treated and untreated individuals are statistically similar ($\chi^2 = 0.3525$, $p = 0.5527$).
Line 253-254 : We have systematically delete this sentence from the paragraph

Line 264 : We have retained the term "adherence", which is understood here as participation.

Line 266 : We have replaced “comparable” with “similar”

Lines 268-275 : We have put this paragraph at the end of the text and took account the reviewer's suggestions. The revised paragraph is as follows : In general, the adherence rate of the studied population in ivermectin treatment at the level of each surveyed HD was below 65% with an exception in Tibati and Tignere HDs where it was above 65% for the two genders, and only for men in Ngaoundere rural HD (**Figure 6**). When HDs were grouped together, the population adherence rate by age group was shown in **Figure 7**

4. Discussion

Line 284-288 : The sentences have been revised as follows : The CMFLs assessed according to Remme and Colleagues [**52**] showed less than 1 mf/sb indicating low parasitic loads in human population, with however an important proportion of parasites circulating in the youngest population. The parasitic loads assessed in the surveyed communities were significantly low compared to those reported in the same communities during the 1998-2002 REMO data collection conducted in Cameroon [**53, 54**]
Line 294: We talked about the introduction of ivermectin treatment in the region on lines 114 and 115.

Lines 302-305 : We have revised this part of the text, reinforcing the discussion as suggested by the reviewer. The revised paragraph is as follows : Also, the existence in the prospected communities of systematic non-compliers to ivermectin representing ≈ 4 to 11% in each surveyed communities and accounting for half of the total infected individuals identified may favour the observed increase in disease prevalence in some HDs

Lines 306-307 : We described these results at the end of the section entitled **Trend in ivermectin treatment and O. volvulus distribution**. The added text is as follows :

Of the 37 individuals tested positive for *O. volvulus* infection out of the 4,814 examined during this 2020 survey, 25 were found among those who had been treated with ivermectin at least once, i.e. 25/3463 (0.72%), and 12 among those who had never been treated (non-compliers), i.e. 12/1351 (0.88%). The mf prevalences in treated and untreated individuals are statistically similar ($\chi^2 = 0.3525$, $p = 0.5527$).

Line 308 : We revised the sentence by replacing "forms" by " infective stage (L_3)"

Lines 319-321 : The sentence was revised according to the suggestion of the reviewer. The revised sentence is as follows : The low intensity of the disease expressed by CMFLs (< 1mf/ss) and the limited number of infected individuals reported in this study suggest active transmission of the parasite in this region but at low level

Line 329 : The sentence was revised as follows : Although the entire Adamaoaua region is under ivermectin treatment region

Lines 336-339 : This sentence was revised following the obsrvation of the reviewer. The revised sentence is as follows : The coverage rates reported and surveyed in each HD of Adamaoua region were statistically comparable only for the Tibati HD, indicating that the CDTI data provided by the local community members and Health system are in agreement

with the participants' statements, which according to WHO confers validation of the CDTI strategy in this HD

Line 356 : In this sentence we replaced "suggests" by "indicates" and we have revised the prevalence and CMFL values in brackets. The revised sentence is : The current profile of the disease indicates low mf prevalences (< 5%) and CMFLs (< 1mf/ss) across the surveyed communities of the Adamaoua Region

Click here to access/download
Revised Article with Changes Highlighted
Manuscript_07_2024_Article Revised with Highlighted changes.docx
