# Supplementary material for: Status of human onchocerciasis transmission in the Adamaoua region of Cameroon after 20 years of ivermectin mass distribution
Source: PLoS Negl Trop Dis. 2025 Mar 4;19(3):e0011511. doi: 10.1371/journal.pntd.0011511 (PMC11925462; doi:10.1371/journal.pntd.0011511)
Supplement: Attachment — Submitted filename: Answers to questions of the Reviewers.docx [file pntd.0011511.s008.docx]

**Answers to questions of Reviewer N°1**

**Reviewer #1**:

The objectives are clearly articular; the study design is appropriate. However, sampling and methodology and limitations at different point are not clearly presented limitations. Also, statistically analysis to make the conclusion appear inadequate

**Answer**

We have provided details on the collection of biological samples used in this study and have indicated the criteria used to select certain communities for data collection during this study. We have also added to the text the inclusion criteria for participants enrolled in this study

**Reviewer #1**: Results need to been well articular towards objectives. As mentioned earlier, statistical analysis could be done better

**Answer**

Our objective was to assess the impact of mass treatments on the evolution of onchocerciasis in the Adamaoua Region. To do this, we assessed the prevalence of nodules and microfilariae at the community and health districts levels. Then we examined the population's adherence to ivermectin treatment accoding to gender and age groups.

We made statistical analyses of data more consistent regarding the adherence of population to ivermectin teatment

**Reviewer #1**: Conclusion appear a bit premature without indebt consideration of sampling, analysis and diseases epidemiology. Limitation of sampling, analysis and disease epidemiology need to be considered in analysis, discussion and conclusion

**Answer**

Yes, we agree that this conclusion is premature since we have not surveyed all the communities and all the eligible individuals in the framewoork of this study. We have used careful language in the text so as not to confirm that we are in a position to eliminate onchocerciasis in this region.

**Reviewer #1**: minor revision

**Answer**

We have made a number of changes to the spelling of certain words, the comprehension of certain part of the text and revised the titles of tables and figures. We would like to thank you for all these observations

**We would like to thank you for your pertinent observations and suggestions to improve the scientific quality of this paper. We remain available to follow your recommendations that may allow the publication of this paper in your journal. Our sincere thanks**

**Answers to questions of Reviewer N°2**

**Reviewer #2**: The objectives of the study are clearly articulated, with a clear testable hypothesis

- the study design is not appropriate to address the stated objectives.e.g the study is impact of control strategy on onchercerciasis transmission and not on elimination as the title suggests.

- the population is clearly described and very appropriate for the hypothesis being tested

- the sample size is quite adequate to address the hypothesis being tested

- correct statistical methods were adopted for data analysis and support for conclusion

- ethical issues concerning the use of corneo- scleral punch in oncherciasis diagnosis was not addressed.

**Answer**

We have modified the title of this paper in line with your comments and suggestions. We have discussed the ethical considerations associated with the transmission of pathogens by corneo-scleral punches used during the collection of skin biopsies.

**Reviewer #2**: The analysis presented were in tandem with analysis plan. The authors did not present the pre.control data of the study communities to enable for proper analysis of the control strategy through CDTI this is another drawback.

The results were clearly but not completely presented

- the figures, tables and images are not sufficient to represent the quantity collected in the study.

**Answer**

Thank you for this observation. We presented in Table 1 the baseline data on mf prevalences that were obtained from the transformation of nodule prevalences according to the formula of Coffeng et al., 2013. We then compared these baseline data with those obtained in this study to monitor the impact of ivermectin mass treatments on the disease transmission. We have added other indications (year of data collection) to columns 6 and 8 of Table 1 to make it easier for understanding.

**Reviewer #2**: The conclusion are fairly supported by the data presented

- the limitations of the study are stated in the manuscript.e.g not using ov16 Elisa to test children in the quest for elimination

- the authors stated how the results of this can be utilized for policy formulation in the control and elimination of oncherciasis

- the public health relevance of the study is adequately stated

**Answer**

Thank you very much for this comment. It has enabled us to express caution in our conclusion and to discuss the results further by mentioning their limitations and envisage the elimination of onchocerciasis in this part of the country.

**Reviewer #2**: The topic should be changed to read ' status of human oncherciasis transmission in the Adamaoua region of Cameroon after 20 years of ivermectin control ' this is due to the fact that the study is assessing impact of ivermectin control and not assessing elimination

**Answer**

Thank you very much, we've changed the title of the paper to reflect your comments.

**We would like to thank you for your pertinent observations and suggestions to improve the scientific quality of this paper. We remain available to follow your recommendations that may allow the publication of this paper in your journal. Our sincere thanks**
