# Supplementary material for: Status of human onchocerciasis transmission in the Adamaoua region of Cameroon after 20 years of ivermectin mass distribution
Source: PLoS Negl Trop Dis. 2025 Mar 4;19(3):e0011511. doi: 10.1371/journal.pntd.0011511 (PMC11925462; doi:10.1371/journal.pntd.0011511)
Supplement: Attachment — Submitted filename: Answers to comments of reviewers.docx [file pntd.0011511.s012.docx]

**Answers to questions of Reviewer N°1**

We would like to thank the reviewers who have examined the scientific content of this work, with the aim of improving it and publishing it in your journal. We present here the responses to Revewer N°1's suggestions and comments in the order in which they appeared in the pdf manuscript he shared with us.

**Abstract**

**Introduction**

We have deleted the first 2 sentences of the introduction as requested and replaced them with the following ones « Significant progress has been made in onchocerciasis control through mass distribution of ivermectin among affected human populations, fostering optimism for disease elimination. However, despite these considerable advances, the elimination of the disease remains a major challenge in many African foci»

**Material and methods**

We replaced the ANOVA and Welch's unequal variances t-test not used for the analyses with the chi-square (χ2) statistic test.

**Results**

We have taken into account the suggestions made in the "Results" section.

**Author summary**

We have revised this section according to the recommendations of the reviewer

**1. Introduction**

Line 79 : We have revised this sentence as follows : Onchocerciasis occurs in 35 countries in the world with Sub-Sahara African (SSA) countries carrying over 99% of all cases in the world

Lines 84- 86: The sentence has been revised as follows: In regard with the gravity of this severe infectious disease, private, public, national and international health authorities/agencies have decided to reduce its prevalence and transmission, then its elimination in affected countries

Line 86 : We deleted thusfare in the following sentence

Lines 94-97 : The sentence was changed as follows : The availability of ivermectin in sufficient quantities has enabled to continue the control of onchocerciasis in West Africa and its extension in endemic countries of Central and East Africa under the management of African Programme for Onchocerciasis Control (APOC) [**22, 23**].

Lines 103-106: The sentence was changed as follows: However, in countries where vector control or CDTI have been successful, the duration of implementation of each strategy was at least 15 years, corresponding to the maximum lifespan of O. volvulus adult worm in human

Lines 112-116 : The sentence was changed as follows : The 1998-2002 REMO surveys conducted in Cameroon resulted in the eligibility of this Adamaoua region for Ivermectin treatment. The CDTI was launched in 1999 in part of the region (Adamaoua II) and was extended in the whole in 2003

Lines 119-121 :  We have added the history of treatment and epidemiological investigations to the text. The sentence was changed as follows “Apart from this 2010-2013 epidemiological survey conducted in 9 villages in part of Adamaoua Region (Adamaoua II), no other survey covering all the region has ever been carried out

**2. Material and methods**

Lines 124-125: The sentence was revised as follows: This study was conducted in the Adamaoua Region of Cameroon located between 7°20’N 13°01’E

Lines 124-125: The rainy season extends from April to October and annual rainfall is 1,500 mm [**41**]. The dry season occurs between November to March with a mean annual temperature of 20-26°C.

Line 133: We replaced “Water Tower” by “WaterShep”

Line 143: Include general comments for the manuscript

Lines 154-155: The sentence was revised as follows: However, the inclusion criteria were as follows: (1) be at least 5 years old, (2) reside continuously in the community, (3) for adults, sign an informed consent form during the registration and assent form for children when necessary

Lines 156-159: The sentence was revised as follows: The study participants' itinerary consisted of 3 stations namely registration, skin biopsy collection and general consultation (**Figure 1**).

Lines 160-161: We deleted the sentence as suggested

Lines 160-161: We replaced “function” by “occupation”

Lines 175-178: The sentence was revised as follows: In the laboratory, the physiological saline contained in each well was pipetted and placed on a slide for observation under a 40x magnification in order to search for and count the microfilariae

### Lines 182-184: The sentence was revised as follows: The answers provided were noted on an individual sheet that was submitted to the laboratory for archiving result of skin biopsy examination

Line 186: Please we kept “Personal information” instead of demographic information

Line 187 : We replaced “Excel file” by “spreadsheet”

Line 197 : We have maintained the Chi-square (χ^2^) test in the manuscript as a method of statistical analysis.

**3. Results**

Line 202 : We have replaced “data” par “variable”

Lines 205-206 : We deleted “with a sex ratio of 1 :1” and “medium” in brackets

Line 210-211 : We have revised the sentence as follows : Of the 29 communities surveyed during this study, 11 (≈ 38%) from 7 HDs were infested by *O. volvulus* microfilariae (**Table 1**)

Lines 211- 212: The sentence was revised as follows: Of these, four were from Meiganga HD, 2 from Ngaoundal HD and 1 from Bankim, Djohong, Ngaoundéré Rural, Ngaoundéré Urbain and Tibati HDs

Line 216 : The statistical method (Chi-square (χ^2^) test) used has been specified in the text and we added a column of p-values in the table 1

Lines 225-228 : This part of the results has been taken into account in the “discussion section”.

Lines 230-231 : The sentence has been revised as follows :  The prevalences of microfilariae and nodules recorded in the HDs surveyed in 2020 are shown in table 2.

Lines 233-241 : **Thank you very much for the observation**. We revised all this paragraph as follows : **Figure 3** shows the mf and nodule prevalences reported in these HDs in 1998-2002, 2010-2013 and 2020 in the study region. Overall, the mf and nodule prevalences reported in this 2020 survey were significantly lower (p < 0.05) compared to those recorded in the 1998-2002 and 2010-2013 surveys (**Figure 3 A & B**). Compared to the 2010-2013 study, a slight increase in mf prevalence was observed in Meiganga HD in 2020 (**Figure 3B**). At the community level, the baseline 1998-2002 mf prevalences ranging from 51.1% (Ngaoundal) to 77.1% (Ngaoundére Rural) have significantly varied from 0% (Banyo or Meiganga) to 2.1% (Ngaoundal) in the 2020 follow-up survey (p < 0.05) (**Figure 4**).

Lines 244-252 : **Trend in ivermectin treatment and *O. volvulus* infection distribution**

We have revised this paragraph according to the reviewer's suggestions. The paragraph now reads as follows : Of the 4,814 participants interviewed for ivermectin treatment, ≈ 28% (N = 1,351) representing almost one third of the studied population said they had never treated with ivermectin, while ≈ 72% (N = 3,463) declared having received ivermectin treatment at least once in the past 6 years (2014-2019). The proportion of participants who had never been treated with ivermectin in the surveyed communities ranged from 1% (Djamtari, Tignere HD) to 11% (Mbamti, Banyo HD). Among those who were treated, 86% (N = 2,977) 249 received the last treatment in 2019, 9.7% (N = 336) in 2018, 4% (N = 141) in 2017 and the remaining 0.3% in 2016, 2015 and 2014. Of the 37 individuals tested positive for *O. volvulus* infection out of the 4,814 examined during the 2020 survey, 25 were found among those who had been treated with ivermectin at least once, i.e. 25/3463 (0.72%), and 12 among those who had never been treated (non-compliers), i.e. 12/1351 (0.88%). The mf prevalences in treated and untreated individuals are statistically similar (χ^2^ = 0.3525, p = 0.5527).

Line 253-254 : We have systematically delete this sentence from the paragraph

Line 264 : We have retained the term "adherence", which is understood here as participation.

Line 266 : We have replaced “comparable” with “similar”

Lines 268-275 : We have put this paragraph at the end of the text and took account the reviewer's suggestions. The revised paragraph is as follows : In general, the adherence rate of the studied population in ivermectin treatment at the level of each surveyed HD was below 65% with an exception in Tibati and Tignere HDs where it was above 65% for the two genders, and only for men in Ngaoundere rural HD (**Figure 6**). When HDs were grouped together, the population adherence rate by age group was shown in **Figure 7**

**4. Discussion**

Line 284-288 : The sentences have been revised as follows : The CMFLs assessed according to Remme and Colleagues [**52**] showed less than 1 mf/sb indicating low parasitic loads in human population, with however an important proportion of parasites circulating in the youngest population. The parasitic loads assessed in the surveyed communities were significantly low compared to those reported in the same communities during the 1998-2002 REMO data collection conducted in Cameroon [**53, 54**]

Line 294: We talked about the introduction of ivermectin treatment in the region on lines 114 and 115.

Lines 302-305 : We have revised this part of the text, reinforcing the discussion as suggested by the reviewer. The revised paragraph is as follows : Also, the existence in the prospected communities of systematic non-compliers to ivermectin representing ≈ 4 to 11% in each surveyed communities and accounting for half of the total infected individuals identified may favour the observed increase in disease prevalence in some HDs

Lines 306-307 : We described these results at the end of the section entitled **Trend in ivermectin treatment and O. volvulus distribution**. The added text is as follows :

Of the 37 individuals tested positive for *O. volvulus* infection out of the 4,814 examined during this 2020 survey, 25 were found among those who had been treated with ivermectin at least once, i.e. 25/3463 (0.72%), and 12 among those who had never been treated (non-compliers), i.e. 12/1351 (0.88%). The mf prevalences in treated and untreated individuals are statistically similar (χ^2^ = 0.3525, p = 0.5527).

Line 308 : We revised the sentence by replacing "forms" by " infective stage (L_3_)"

Lines 319-321 : The sentence was revised according to the suggestion of the reviewer. The revised sentence is as follows : The low intensity of the disease expressed by CMFLs (< 1mf/ss) and the limited number of infected individuals reported in this study suggest active transmission of the parasite in this region but at low level

Line 329 : The sentence was revised as follows : Although the entire Adamaoaua region is under ivermectin treatment region

Lines 336-339 : This sentence was revised following the obsrvation of the reviewer. The revised sentence is as follows : The coverage rates reported and surveyed in each HD of Adamaoua region were statistically comparable only for the Tibati HD, indicating that the CDTI data provided by the local community members and Health system are in agreement with the participants' statements, which according to WHO confers validation of the CDTI strategy in this HD

Line 356 : In this sentence we replaced "suggests" by "indicates" and we have revised the prevalence and CMFL values in brackets. The revised sentence is : The current profile of the disease indicates low mf prevalences (< 5%) and CMFLs (< 1mf/ss) across the surveyed communities of the Adamaoua Region
